# Next-generation of BBQ analogues that selectively target breast cancer

**DOI:** 10.3389/fchem.2024.1396105

**Published:** 2024-06-21

**Authors:** Jennifer R. Baker, Jayne Gilbert, Nicholas S. O’Brien, Cecilia C. Russell, Adam McCluskey, Jennette A. Sakoff

**Affiliations:** ^1^ Chemistry, School of Environmental and Life Sciences, The University of Newcastle, Callaghan, NSW, Australia; ^2^ Experimental Therapeutics Group, Department of Medical Oncology, Calvary Mater Newcastle Hospital, Waratah, NSW, Australia

**Keywords:** breast cancer, AhR, CYP1, SULT1A1, molecular modelling

## Abstract

We previously reported on the interaction of 10-chloro-7*H*-benzo[*de*]benzo[4,5]imidazo[2,1-*a*]isoquinolin-7-one (10-Cl-BBQ) with the Aryl hydrocarbon Receptor (AhR) and selective growth inhibition in breast cancer cell lines. We now report on a library of BBQ analogues with substituents on the phenyl and naphthyl rings for biological screening. Herein, we show that absence of the phenyl Cl of 10-Cl-BBQ to produce the simple BBQ molecule substantially enhanced the growth inhibitory effect with GI_50_ values of 0.001–2.1 μM in select breast cancer cell lines MCF-7, T47D, ZR-75-1, SKBR3, MDA-MB-468, BT20, BT474 cells, while having modest effects of 2.1–7 μM in other cell lines including HT29, U87, SJ-G2, A2780, DU145, BE2-C, MIA, MDA-MB-231 or normal breast cells, MCF10A (3.2 μM). The most potent growth inhibitory effect of BBQ was observed in the triple negative cell line, MDA-MB-468 with a GI_50_ value of 0.001 μM, presenting a 3,200-fold greater response than in the normal MCF10A breast cells. Additions of Cl, CH_3_, CN to the phenyl ring and ring expansion from benzoimidazole to dihydroquinazoline hindered the growth inhibitory potency of the BBQ analogues by blocking potential sites of CYP1 oxidative metabolism, while addition of Cl or NO_2_ to the naphthyl rings restored potency. In a cell-based reporter assay all analogues induced 1.2 to 10-fold AhR transcription activation. Gene expression analysis confirmed the induction of CYP1 oxygenases by BBQ. The CYP1 inhibitor α-naphthoflavone, and the SULT1A1 inhibitor quercetin significantly reduced the growth inhibitory effect of BBQ, confirming the importance of both phase I and II metabolic activation for growth inhibition. Conventional molecular modelling/docking revealed no significant differences between the binding poses of the most and least active analogues. More detailed DFT analysis at the DSD-PBEP86/Def-TZVPP level of theory could not identify significant geometric or electronic changes which would account for this varied AhR activation. Generation of Fukui functions at the same level of theory showed that CYP1 metabolism will primarily occur at the phenyl head group of the analogues, and substituents within this ring lead to lower cytotoxicity.

## 1 Introduction

With more than 18 million cases of cancer worldwide, female breast cancer is the most commonly diagnosed cancer with an estimated 2.3 million new cases in 2020 (11.7%) (Sung et al., 2021). Established risk factors for breast cancer include family history, obesity, estrogen exposure, as well as the inheritance of mutated versions of DNA repair genes such as BRCA1 and BRCA2, however, other factors such as smoking and exposure to environmental chemicals also contribute (Gray et al., 2017; Łukasiewicz et al., 2021; Chong et al., 2023). These chemicals include: i) halogenated aryl hydrocarbons (HAH) such as polychlorinated biphenyls (PCB), dichlorodiphenyltrichloroethane (DDT), dichlorodiphenyldichloroethylene (DDE), and 2,3,7,8-tetrachlorodibenzo-*p*-dioxin (TCDD); ii) polyaromatic hydrocarbons (PAH) such as benzo[*a*]pyrene (BAP), and dimethylbenz(*a*)anthracene (DMBA); iii) phthalates such as benzyl butyl phthalate (BBP); and iv) bisphenol compounds such as bisphenol A (BPA), bisphenol S (BPS) and tetrabromobisphenol A (TBBPA) all of which are fat soluble ligands for the Aryl hydrocarbon Receptor (AhR) (Wang et al., 2016; Gray et al., 2017; Chong et al., 2023; Shan et al., 2023). Their carcinogenic effect is linked to their metabolic activation to reactive intermediates and DNA damage (Greiner et al., 1980; Nebert et al., 2004). An example of this process is the widely used DMBA-induced surrogate model of breast cancer tumorigenesis in animal studies via AhR activation and metabolic bio-activation (Russo and Russo, 1996; Trombino et al., 2000). Moreover, once the tumour is established various endogenous and exogenous ligands of AhR continue to support tumour growth and modulation of the tumour microenvironment (Casey et al., 2015; Baker et al., 2020).

The AhR is a ligand dependent transcription factor and a member of the basic helix-loop-helix/Per-ARNT-SIM transcription factor family. The AhR localises in the cell cytosol and is complexed with two molecules of heat shock protein 90 (Hsp90), co-chaperone p23 and the Aryl hydrocarbon interacting protein (AIP). After ligand binding, the receptor translocates to the nucleus and heterodimerises with the nuclear transporter, ARNT (AhR nuclear transporter). This dimer modulates the expression of targets by binding to xenobiotic response elements (XRE) and co-regulators (Chong et al., 2023). A key target is the expression of xenobiotic metabolic enzymes, such as the P450 drug metabolising cytochromes (1A1, 1A2, 1B1), that facilitate the hydroxylation of aromatic substrates (Chong et al., 2023). These phase I enzymes work in concert with phase II enzymes including sulfotransferases (SULT1A1), with the collective function of detoxifying the substrate to a highly soluble form that is readily excreted (Bugano et al., 2008). However, hydroxylation and subsequent sulfation of specific aromatic molecules can also produce highly reactive intermediates that bind DNA and induce cell death (Meng et al., 2005; 2006; Rothman et al., 2015). These reactive intermediates are produced when the sulphate moiety departs, rendering the compounds strong electrophiles (Meng et al., 2006; Rothman et al., 2015). The potency and selectivity of these compounds in breast cancers is determined by their chemical composition as well as the overexpression of AhR and SULT1A1 proteins (Huang et al., 2014; Gilbert et al., 2017; Baker et al., 2020; Gilbert et al., 2020; Safe and Zhang, 2022), with the latter presenting as a potential clinical biomarker for targeted therapy (Huang et al., 2014).

**FIGURE 1:**
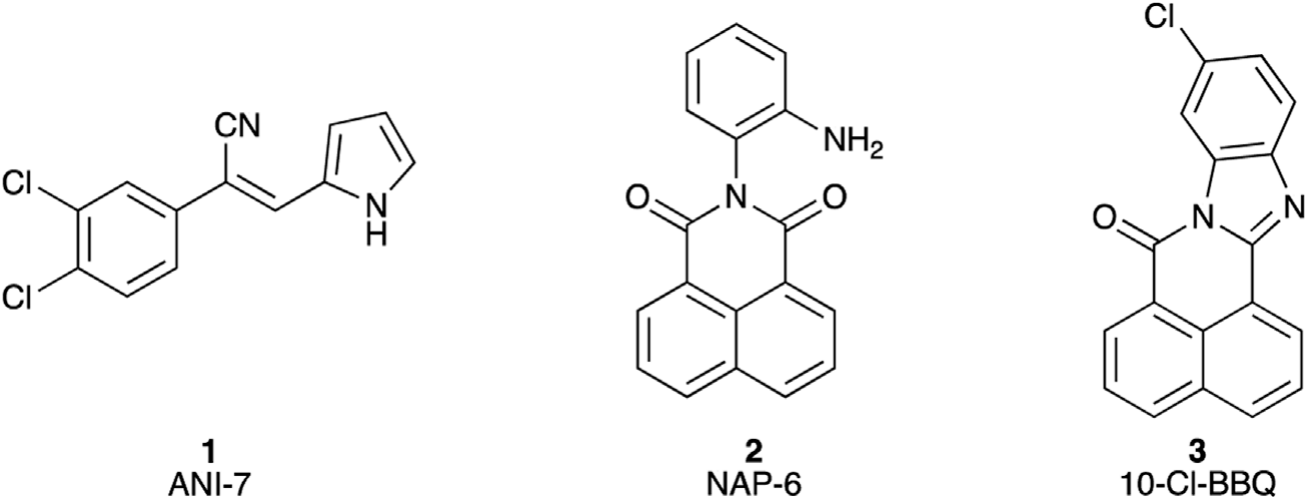
Chemical structures of (**1**) (*Z*)-2-(3,4-dichlorophenyl)-3-(1*H*-pyrrol-2-yl)acrylonitrile (ANI-7); (**2**) 2-(2-aminophenyl)-1*H*-benzo[de]isoquinoline-1,3(2*H*)-dione (NAP-6), (**3**) 10-chloro-7*H*-benzo[*de*]benzo[4,5]imidazo[2,1-*a*]isoquinolin-7-one (10-Cl-BBQ).

We have explored the link between the AhR and breast cancer in the development of therapeutic small molecules (Gilbert et al., 2017; Baker et al., 2018; Gilbert et al., 2020; Baker et al., 2021a; Baker et al., 2021b). Our efforts have generated the halogenated aryl hydrocarbon ANI-7 (**1**; (*Z*)-2-(3,4-dichlorophenyl)-3-(1*H*-pyrrol-2-yl)acrylonitrile) and the polyaromatic hydrocarbon NAP-6 (**2**; (*Z*)-2-(2-aminophenyl)-1*H*-benzo[*de*]isoquinoline-1,3(2*H*)-dione) (Figure 1) as two molecules displaying more than 500-fold selective targeting of certain breast cancer cell lines compared with normal breast cells or other tumour types via activation of the AhR pathway (Gilbert et al., 2017; Gilbert et al., 2020). We have also demonstrated that 10-Cl-BBQ (**3**; 10-chloro-7*H*-benzo[*de*]benzo[4,5]imidazo[2,1-*a*]isoquinolin-7-one) (Figure 1), a known AhR ligand, is up to 150-fold selective in targeting certain breast cancer cell lines compared with normal cells or other tumour types (Baker et al., 2021a; Elson et al., 2023). Leveraging this data, we sought to further explore the breast cancer selectivity and activation of the AhR/CYP1/SULT1A1 axis of a library of substituted BBQ analogues, in cell line models of the disease.

## 2 Results

### 2.1 BBQ analogues

**SCHEME 1:**
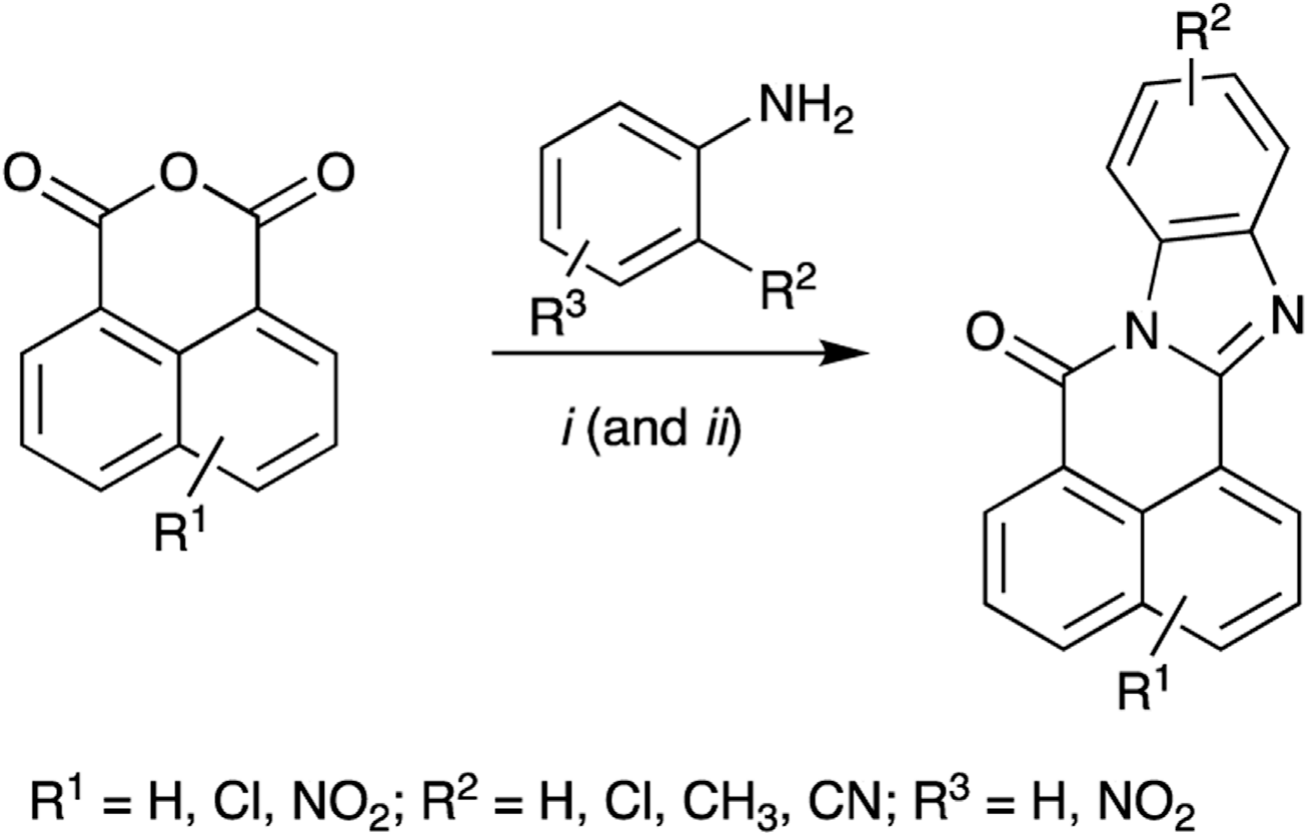
Reagents and Conditions: (i) AcOH reflux; (ii) R^2^ = NO_2_; Fe(powder), AcOH, reflux.

**FIGURE 2:**
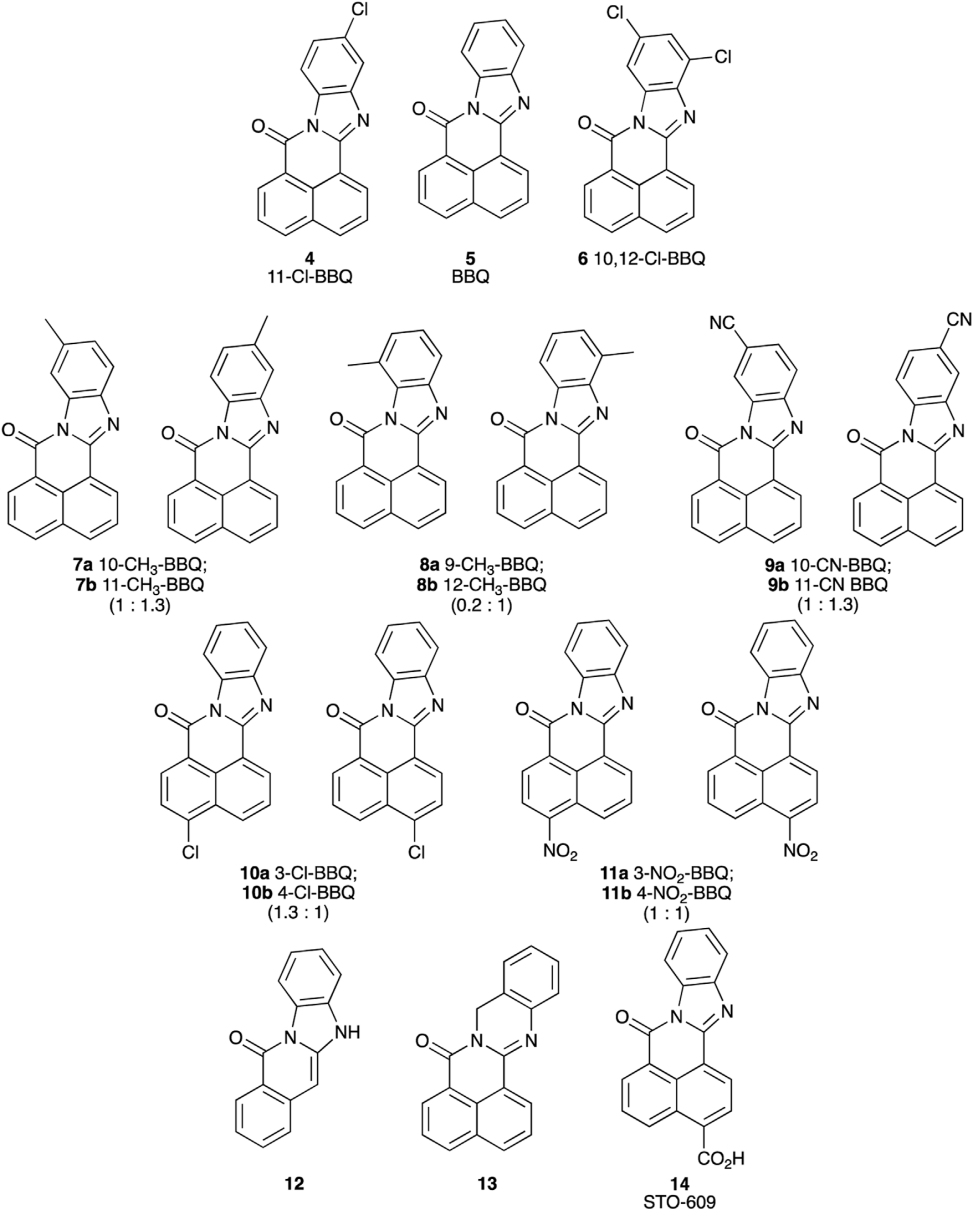
Structure of BBQ analogues. The structurally similar known AhR ligand, STO-609 (**14**, commercial analogue), has been included for comparison. Values in parentheses represent the ^1^H NMR calculated relative isomer ratios.

Each BBQ analogue was accessed by condensation of the required anhydride and diamine under acetic acid reflux conditions (Scheme 1). Reaction workup (see experimental) afforded the desired BBQ analogues (Figure 2). With analogues **6** - **11**, in keeping with prior reports of analogues of this nature, a mixture of regioisomers was obtained (with the exception of analogue **6**: due to steric constraints, only the 10,12-dichloro analogue formed). ^1^H, ^13^C NMR and UPLC-MS analysis was consistent with the presence of a regioisomeric mixture in all cases identified (see Figure 2 for detail).

### 2.2 Growth inhibition

Each BBQ analogue, **2**-**14** was screened for their growth inhibition ability (GI_50_) in a broad panel of cancer cell lines and a non-cancer breast cell line (MCF10A), using the MTT growth inhibition assay (Table 1) (Baker et al., 2021b). The cancer panel comprised HT29 (colorectal), U87 (brain), SJ-G2 (brain), A2780 (ovary), Du145 (prostate), BE2-C (neuronal), MIA (pancreas), H460 (lung, ER+), A431 (vulva, ER+ (estrogen receptor positive)), and a wide selection of breast cancer cell lines with varying receptor status including MCF-7 (ER + luminal A), T47D (ER/PR+ (progesterone receptor positive)luminal A), ZR-75-1 (ER/PR + luminal A), BT-474 (ER/PR/HER2+ luminal B), SKBR-3 (HER2+), MDA-MB-468, BT20, and MDA-MB-231 (basal, triple negative (TN) for ER, PR, and HER2).

**TABLE 1:** The MTT growth inhibition assay (72 h, GI_50_, μM) was used to determine the potency of the BBQ analogues in a broad panel of cell lines. NAP-6 (**2**) and STO-609 (**14**) were included for comparison (Gilbert et al., 2020). The cancer selectivity was determined by comparing the GI_50_ for each cancer cell line with that of the normal breast cell line (MCF10A), with four levels of shading used to highlight the fold difference; 2–10 , 10–100 , 100–1000 , and greater than 1,000‐fold .

	HT29	U87	SJ-G2	A2780	Du145	BE2-C	MIA	H460	A431	MCF-7	T47D	ZR-75-1	SKBR3	MDA-MB-468	BT20	BT474	MDA-MB-231	MCF10A
Compound	Colon	Brain (GBM)	Brain (GBM)	Ovary	Prostate	Neuron	Pancreas	Lung (ER)	Skin (ER)	Breast (ER)	Breast (ER/PR)	Breast (ER/PR)	Breast (HER2)	Breast (TN)	Breast (TN)	Breast (ER/PR/HER2)	Breast (TN)	Breast (Normal)
**2** (Gilbert et al., 2020)	32 ± 2	>50	>50	21 ± 4	>50	>50	>50	15 ± 7.5	0.25 ± 0.12	0.70 ± 0.12	0.18 ± 0.02	0.12 ± 0.03	0.22 ± 0.02	0.10 ± 0.02	14 ± 1.5	0.43 ± 0.07	35 ± 3.0	31 ± 1.5
**3**	21 ± 3.3	18 ± 3.8	33 ± 8.3	6.2 ± 0.46	22 ± 3.1	17 ± 4.3	>50	7.2 ± 1.16	12 ± 1.8	7.9 ± 1.14	11 ± 1.4	0.48 ± 0.24	0.30 ± 0.12	0.56 ± 0.23	33 ± 4	>50	17 ± 6.1	3.9 ± 0.21
**4**	18 ± 3.2	18 ± 1.8	8.8 ± 1.1	6.0 ± 0.32	12 ± 0.33	10.0 ± 1.7	31 ± 3.3	8.2 ± 1.92	10.0 ± 2.0	5.3 ± 0.39	8.6 ± 2.2	0.81 ± 0.43	0.26 ± 0.07	0.88 ± 0.17	28 ± 3	>50	9.0 ± 1.0	4.1 ± 0.20
**5**	2.1 ± 0.30	7.0 ± 0.033	4.6 ± 0.23	2.2 ± 0.21	4.1 ± 0.03	5.1 ± 0.74	5.2 ± 0.73	0.61 ± 0.13	0.050 ± 0.013	2.1 ± 0.44	0.20 ± 0.073	0.026 ± 0.015	0.032 ± 0.014	0.001 ± 0.0004	0.41 ± 0.39	1.5 ± 1.3	5.5 ± 1.8	3.2 ± 0.32
**7**	5.0 ± 0.07	17 ± 2.7	>50	5.2 ± 0.63	37 ± 7.8	4.9 ± 0.09	8.1 ± 0.97	45 ± 8.0	4.8 ± 0.7	3.6 ± 0.40	3.1 ± 0.17	0.19 ± 0.045	1.2 ± 0.93	0.35 ± 0.13	5.2 ± 2.0	>50	25 ± 7.1	4.7 ± 0.12
**8**	3.7 ± 0.69	38 ± 2.5	24 ± 8.4	11 ± 0.58	38 ± 6.9	15 ± 10	20 ± 10	27 ± 6.7	0.56 ± 0.14	2.8 ± 0.61	0.80 ± 0.11	0.59 ± 0.38	0.27 ± 0.027	0.056 ± 0.024	1.4 ± 0.80	4.6 ± 2.7	8.2 ± 3.3	2.0 ± 1.3
**9**	44 ± 6.3	43 ± 4.0	26 ± 3.8	7.0 ± 0.64	45 ± 1.7	>50	41 ± 9.5	41 ± 5.9	1.10 ± 0.10	25 ± 8.3	4.2 ± 0.73	0.089 ± 0.040	0.27 ± 0.018	0.074 ± 0.021	9.3 ± 8.4	>50	38 ± 6.0	7.8 ± 1.62
**10**	2.9 ± 0.18	37 ± 5.8	4.8 ± 0.44	4.6 ± 0.25	>50	7.0 ± 0.23	9.8 ± 3.1	5.7 ± 1.48	0.15 ± 0.036	0.14 ± 0.045	0.18 ± 0.019	0.072 ± 0.030	0.061 ± 0.024	0.0056 ± 0.0017	0.27 ± 0.12	0.56 ± 0.42	29 ± 10.3	4.2 ± 0.38
**11**	0.30 ± 0.11	0.55 ± 0.11	0.61 ± 0.14	0.010 ± 0.003	0.32 ± 0.049	0.74 ± 0.13	1.4 ± 0.45	0.00019 ± 0.0001	0.088 ± 0.031	0.0065 ± 0.0038	0.013 ± 0.0045	0.0027 ± 0.0022	0.036 ± 0.006	0.0015 ± 0.0008	0.0084 ± 0.004	0.15 ± 0.053	2.1 ± 0.29	0.0031 ± 0.002
**12**	0.21 ± 0.056	>50	43 ± 10.3	3 ± 2.10	>50	0.24 ± 0.079	0.62 ± 0.16	>50	0.48 ± 0.10	0.12 ± 0.05	0.164 ± 0.083	0.17 ± 0.023	0.20 ± 0.071	0.141 ± 0.074	0.16 ± 0.048	>50	>50	0.54 ± 0.07
**13**	>50	>50	56 ± 3.7	>50	>50	42 ± 1.5	38 ± 1.0	>50	43 ± 4.4	32 ± 1.9	40 ± 3.3	35 ± 1.0	47 ± 2.7	36 ± 3.8	>50	>50	>50	38 ± 1.2
**14**	-	25 ± 1.7	13 ± 1.2	12 ± 0.91	21 ± 2.0	6.9 ± 1.6	20 ± 0.67	20 ± 0.33	28 ± 5.1	>50	16 ± 0.58	24 ± 3.3	21 ± 0.67	6.13 ± 3.05	50 ± 0.0	>50	50 ± 0.0	15 ± 0.58

Our previously identified breast cancer selective molecule **2** (NAP-6) (Gilbert et al., 2020) was inactive in U87, SJ-G2, DU145, BE2C, MIA, and MCF10A cells (defined here as GI_50_ > 50 μM), more active in MDA-MB-231 A2780, HT29, H460 and BT20 (GI_50_ 35–14 μM) and sub-micromolar potent in A431, MCF-7, T47D, ZR-75-1, SKBR3, MDA-MB-468, BT474 (GI_50_ 0.1–0.7 μM). With A431, and the breast cancer cell lines T47D, ZR-75-1, SKBR3, MDA-MB-468 showing a 100 to 300-fold increase in growth inhibition potency compared with the MCF10A cells.

Dichloro analogue **6** was insoluble in the compound testing media, and thus not screened. The remaining BBQ analogues produced GI_50_ values that varied between inactive (GI_50_ > 50 μM) (**3**, MIA, BT474; **4**, BT474; 7, SJ-G2, BT474; **9**, BE2-C, BT474; **10**, DU145; **12**, U87, Du145, H460, BT474, MDA-MB-231; **13**, HT29, U87, A2780, Du145, H460, BT20, BT474, MDA-MB-231; **14**, MCF-7, BT474) to low nanomolar (**5**, MDA-MB-468; **10**, MDA-MB-468; **11**, MCF-7, ZR-75-1, MDA-MB-468, BT20, and MCF-10A) and picomolar (**11**, H460).

The introduction of a phenyl disposed Cl-moiety with **3** and **4**, yielded analogues with a similar growth inhibition profile across the cell line panel examined, with good inhibition in the breast cancer cell lines ZR-75-1, SKBR3 and MDA-MB-468 cells (GI_50_’s 0.26–0.88 μM) and moderate to no growth inhibition in all other cell lines (GI_50_’s 3.9 to >50 μM). The greatest difference between the growth inhibition in these breast cancer cell lines and that of the normal breast cells was 16-fold (c.f. **4**, GI_50_ 0.26 μM SKBR3 vs. 4.1 μM MCF10A cells). These data suggest potentially two roles for the Cl-moiety. Firstly, as a potential metabolic blocking group inhibiting CYP activation of a particular oxidation site (c.f. **3** and **4**), and secondly as an electron withdrawing moiety, activating sites adjacent (or distal sites dependent on system conjugation) to the Cl-substituent (Zhang and Tang, 2018; Fang et al., 2019). While di-Cl additions produced an insoluble compound **6**, the chlorine free analogue **5**, represents the core BBQ scaffold. Analogue **5** induced strong growth inhibition in H460, A431 (GI_50_ 0.61 and 0.05 μM), and the breast cancer cell lines T47D, ZR-75-1, SKBR3, MDA-MB-468, and BT20 cells (GI_50_ 0.001–0.41 μM), with only moderate inhibition in all other cell populations (GI_50_ 1.5–7 μM). The growth inhibition in these cell lines compared with normal cells was **5** to 3200-fold (c.f. GI_50_ 0.001 μM MDA-MD-468 vs. 3.2 μM MCF10A cells), representing a significant enhancement in potency and selectivity. Methyl (CH_3_) or cyano (CN) additions to the phenyl ring produced **7** (10-methyl and 11-methyl), **8** (9-methyl and 12-methyl) and **9** (10-cyano and 11-cyano) which resulted in a potency reduction compared with **5**, supporting the concept that blocking substituents on the phenyl ring potentially reduces oxidation by CYP at these and adjacent sites.

Repositioning of the Cl-moiety to the naphthyl rings with **10**, with no substituent on the phenyl moiety, saw a re-introduction of potency across all cell lines (GI_50_ 0.0056 μM MDA-MB-468 to 9.8 mM in MIA cells). Indeed, **10** induced growth inhibition in skin A431 (GI_50_ 0.15 μM), and the breast cancer cell lines MCF-7, T47D, ZR-75-1, SKBR-3, MDA-MB-468, BT20, BT474 (GI_50_ 0.0056–0.56 μM) with a selectivity relative to the normal cell line of 7.5 to 750-fold (c.f. GI_50_ 0.0056 μM MDA-MD-468 vs. 4.2 μM in MCF10A cells), showing a clear re-establishment of selectivity and potency in a manner similar to **5**. The addition of a NO_2_ moiety to the naphthyl ring produced **11** (3-NO_2_ and 4-NO_2_), which induced a significant increase in growth inhibition across all cell lines (GI_50_ 0.00019 μM H460 to 2.1 μM MDA-MB-231), with the highest activity noted in H460 cells (0.00019 μM), breast cancer cell lines MCF-7 (GI_50_ 0.0065 μM), ZR-75-1 (GI_50_ 0.0027 μM), MDA-MB-468 (GI_50_ 0.0015 μM), and BT20 (GI_50_ 0.0084 μM), and normal breast cells MCF10A (GI_50_ 0.0031 μM). The profile of growth inhibition suggests that **11** is mediating its effects via a similar pathway to the other BBQ analogues, but with an additional broad off-target(s), culminating in a highly potent broad spectrum compound. The removal of one ring from the naphthyl system with **12** had a profound, detrimental impact on both overall potency and selectivity, consistent with the need for an extended conjugated system, as present in the BBQ polyaromatic scaffold (Baker et al., 2021a). This conjugation requirement is reinforced by the further potency and selectivity reduction evident with the methylene spaced **13**. The commercially available **14**, with a naphthyl disposed carboxylate displayed little potency and selectivity, presumably a combination of the, relatively, poor electron withdrawing nature and the anticipated lower cell permeability of the carboxylate moiety (Ballatore et al., 2013).

### 2.3 Activation of AhR-CYP1-SULT1A1 pathway

**FIGURE 3:**
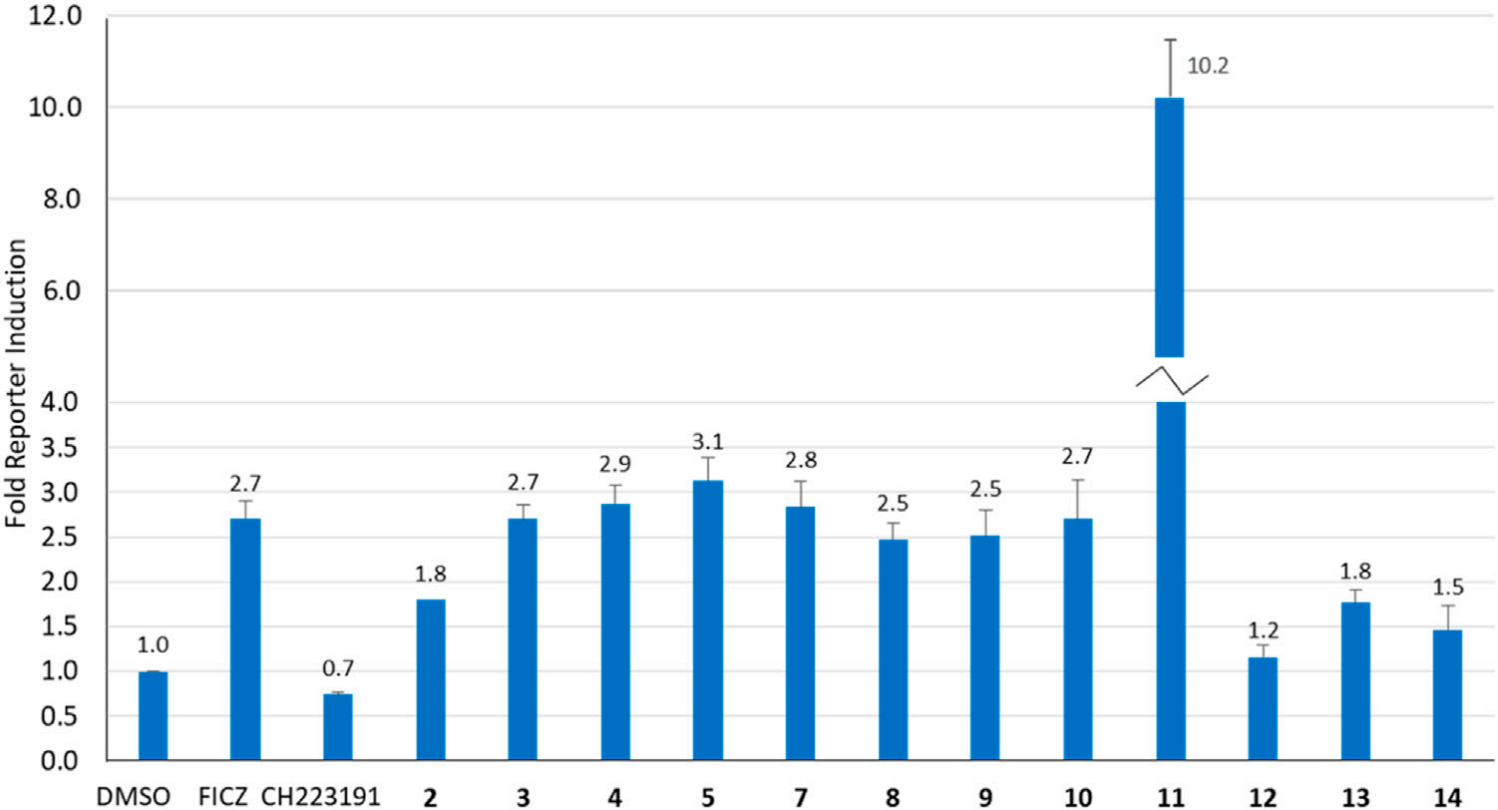
Analysis of AhR activation (fold increase) in response to BBQ analogues (1 µM) using a transcription reporter assay (HT29-Lucia™ AhR cells) after 24 h. FICZ (AhR ligand 0.5 μM) and CH223191 (AhR inhibitor 2.5 μM) were included as positive and negative controls, respectively.

Our previous studies have shown that the activity of NAP-6 (**2**) and 10-Cl-BBQ (**3**) required the activation of the AhR pathway and the induction of CYP1 family of phase I metabolising enzymes (Gilbert et al., 2020; Baker et al., 2021a). Therefore, we sought to determine the activation of AhR using a stable AhR reporter assay in HT29-Lucia™ AhR cells, in the presence of each BBQ analogue (Figure 3). Activation of the AhR was indicated by enhanced luminescence caused by the translocation of the AhR to the nucleus of the cell and interaction with the xenobiotic response elements imbedded in the luciferase gene. The analysis showed that all BBQ analogues induced AhR translocation and binding to the xenobiotic response element of the target luciferase gene, but at varying levels. Indeed, analogues **3**-**5** and **7**-**10** induced a response comparable to that of the natural AhR ligand FICZ (6-formylindolo[3,2-*b*]carbazole) (2.7-fold activation of AhR), which was slightly stronger than the signal noted with NAP-6 analogue **2** (1.8-fold). Consistent with the enhanced cytotoxicity observed, analogue **11** induced the greatest level of AhR activation (10.2-fold), while **12**, **13**, and **14** (1.2, 1.8 and 1.5-fold), induced a response similar to that of **2** (1.8-fold).

While induction of AhR activity is required to stimulate CYP-induced metabolic activation of **1**, **2** and **3** (Gilbert et al., 2017; Gilbert et al., 2020), the magnitude of AhR induction by the BBQ analogues did not directly correlate with the potency of growth inhibition or to tumour type selectivity. For example, compounds **3**, **4**, and **5** induced a similar effect on AhR induction (2.7, 2.9, 3.1-fold), however, compound **5** was substantially more potent at growth inhibition (GI_50_ 0.001 µM, MDA-MB-468 cells), than **3** (GI_50_ 0.56 μM, MDA-MB-468 cells) and **4** (GI_50_ 0.88 μM, MDA-MB-468 cells). This suggests that additional factors are at play, such as the limited ability of **3**, and **4** to undergo CYP induced metabolic conversion caused by the Cl-blocking moieties, rather than a lack of AhR stimulation. An exception to the lack of correlation between AhR activity and growth inhibition was analogue **11** which induced the greatest growth inhibition and the greatest induction of AhR activity.

### 2.4 Induction of CYP1 expression

**FIGURE 4:**
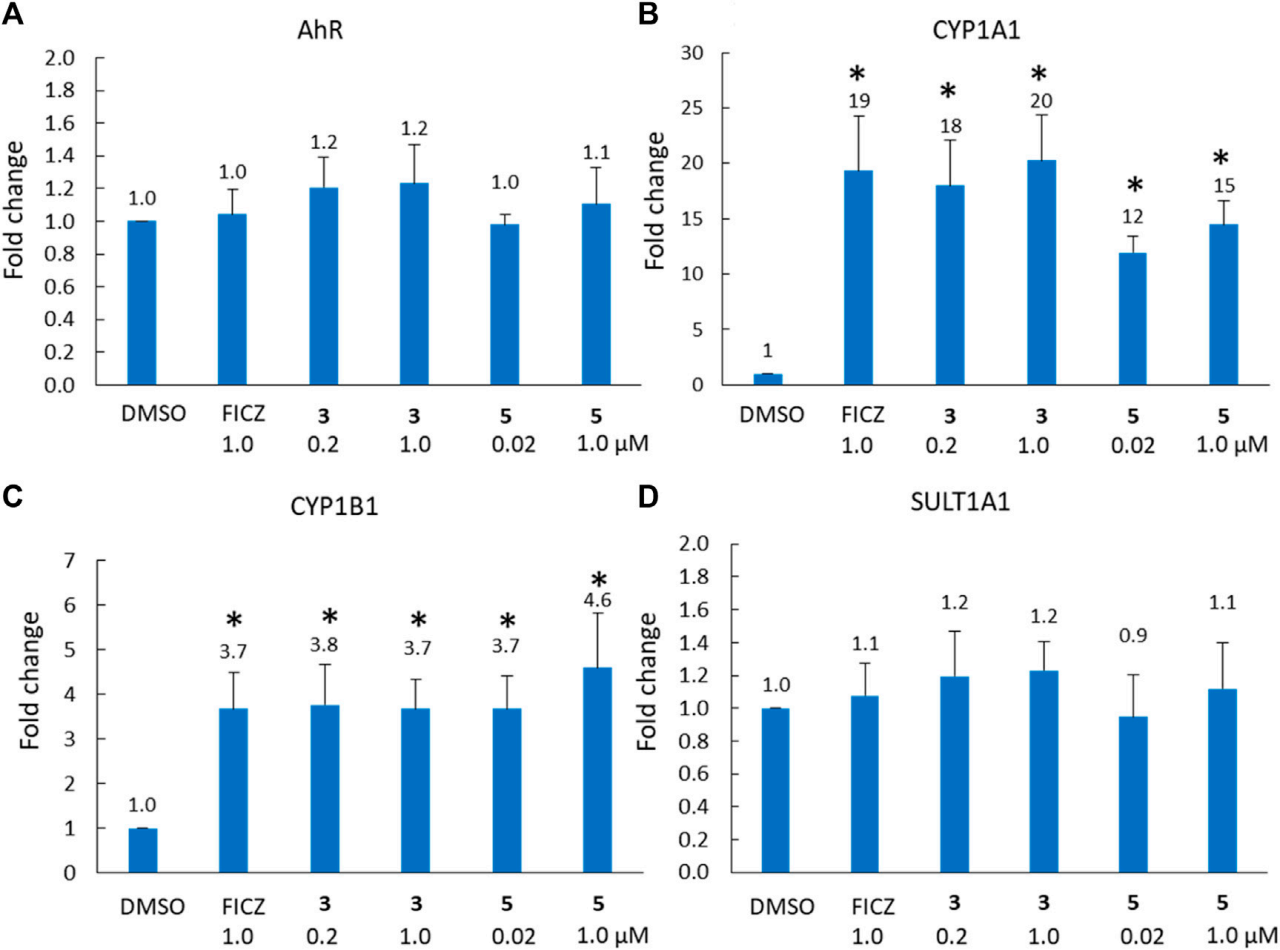
Analysis of **(A)** AhR, **(B)** CYP1A1, **(C)** CYP1B1 and **(D)** SULT1A1 gene expression (fold-increase in mRNA compared with DMSO control) in MDA-MB-468 cells after 6 h exposure to compound **3** (0.2 μM = GI_50_, 1 μM) and FICZ (1 μM) and compound **5** (0.02 μM = GI_50_, 1 μM). * = *p* < 0.01 difference from DMSO control, using a paired T-test with a two-tailed distribution.

To further explore the role of the AhR pathway in the biological response of our BBQ analogues we chose to investigate the expression of downstream genes in the most sensitive cell line MDA-MB-468 cells, in response to compounds **3** and **5**. These analogues were specifically chosen because they were synthesised without the production of regioisomers, their structural differences are small while their biological differences are large, c.f. **3** possesses a phenyl Cl and presented with minimal growth inhibition, while **5** lacks a phenyl substituent and presented with strong growth inhibition. Thus, we sought to examine the effect of **3** and **5**, on AhR, CYP1A1, CYP1B1 and SULT1A1 expression in MDA-MB-468 cells, at 1 μM and the GI_50_ value (0.2 and 0.02 μM, respectively) (Figure 4). Not surprisingly, the expression of AhR was not significantly altered following treatment with either **3** or **5** (Figure 4A); however, CYP1A1 and CYP1B1 expression was significantly increased (*p* < 0.01) within 6 h of treatment (Figures 4B,C). Indeed, the expression of CYP1A1 increased by 18 and 20-fold for analogue **3**, which was comparable to that of the endogenous AhR ligand, FICZ (19-fold). Analogue **5** also induced CYP1A1 expression, and the response at the GI_50_ value of 0.02 μM (12-fold) was comparable to that at 1 μM (15-fold), suggesting that the CYP1A1 activity may be rate limiting, in that substantially higher concentrations do not induce a commensurate increase in expression. Both analogues also significantly (*p* < 0.01) induced CYP1B1 expression (3.7-fold for **3**; 4.6-fold for **5** at 1 μM) to a comparable level to that of FICZ (3.7-fold, 1 μM). Neither analogue altered the expression of SULT1A1 (Figure 4D). Collectively, the gene expression analysis following treatment with **3** and **5** was equivalent, suggesting that the differences in growth inhibition induced by these compounds was not caused by an inability to induce the AhR pathway, but rather the ability of the analogues to be bio-activated.

### 2.5 Activation of CYP1 and SULT1A1 functionality

**FIGURE 5:**
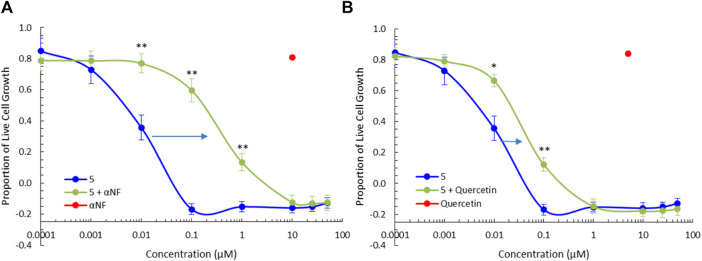
CYP1 and SULT1A1 inhibition ameliorates the effect of **5**. Growth inhibition (72 h, MTT assay) of **5** in the presence of the **(A)** CYP1 family inhibitor, α-naphthoflavone (αNF, 10 μM) and **(B)** SULT1A1 inhibitor, quercetin (5 μM), in MDA-MB-468 breast cancer cells. Significant differences between growth inhibition of compound **5** with (green) and without inhibitors (blue) is shown at the *p* < 0.01 ** and *p* < 0.05 * level, using a paired T-test with a two-tailed distribution. The effect of αNF and quercetin at the single concentration of 10µM and 5 µM respectively is shown in red as a single value.

To further clarify the role of phase I and phase II metabolic activation of the BBQ compounds herein, we examined the effect of CYP1 and SULT1A1 inhibition on the growth inhibitory effect of compound **5** in MDA-MB-468 cells (Figure 5) and MCF10A cells (Supplementary Material). The co-administration of the CYP1 inhibitor α-naphthoflavone (Khojasteh et al., 2011) (10 μM) with **5**, significantly reduced the growth inhibition response as indicated by the shift in the growth inhibition curve to the right, from a GI_50_ of 0.005 μM–0.18 µM (36-fold reduction) (Figure 5A). Similarly, the co-administration of the SULT1A1 inhibitor quercetin (Pacifici, 2004) (5 μM) with **5**, also significantly reduced the growth inhibitory effect of **5**, from GI_50_ of 0.005 μM–0.023 μM (4.6-fold reduction) (Figure 5B). A similar effect was noted for **3** but at a reduced level (Supplementary Material). Neither **3**, **5**, αNF, or quercetin affected growth in normal MCF10A cells (Supplementary Material). This analysis confirms that both CYP1 and SULT1A1, activity is needed to induce the growth inhibitory effect of the BBQ analogue.

We, and others have noted that the activation of the AhR pathway by ligands such as **1**-**7** results in specific cytotoxicity towards breast cancer cell lines. This was noted in the cases of **1**-**6**, but as with the level of AhR expression, 3-NO_2_ **11** was an outlier. This BBQ analogue displayed high potency across most cell lines examined, while retaining a preference for ER-positive cell lines. This resulted in excellent levels of MTT cytotoxicity with GI_50_ values: 0.00019 (H460), 0.0065 (MCF-7), 0.0013 (T47D), 0.0027 (ZR-75-1), 0.0360 (SKBR3), 0.0015 (MDAMB468) and 0.0084 (BT20) μM. Given the significant AhR amplification (Figure 2), logically this enhanced potency originates from the AhR/CYP1/SULT1A1 axis, and the magnitude of the effect (with no differences in the predicted binding poses) is most likely due to electronic effects.

### 2.6 Computational approaches–Prediction of AhR activation

**FIGURE 6:**
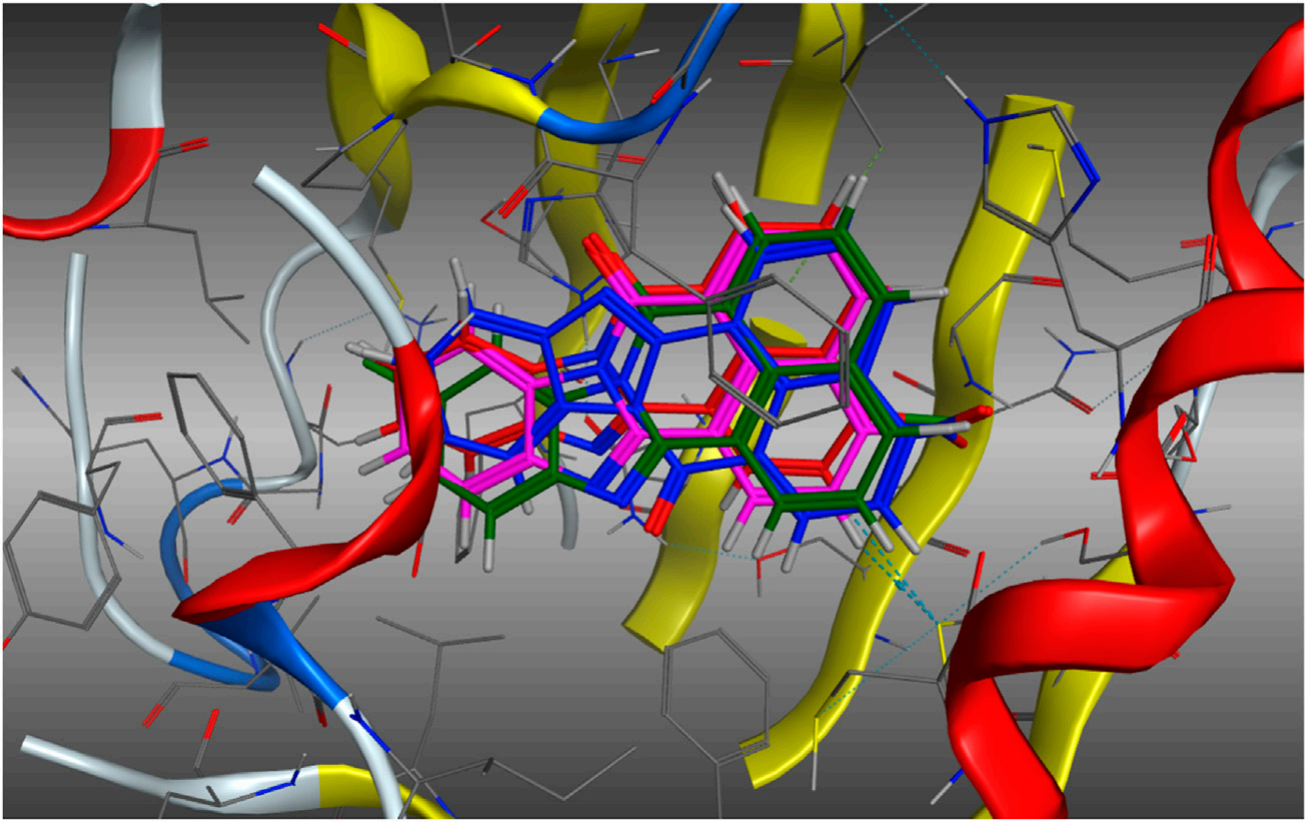
Lowest energy (top scoring) poses of compound **5** (E = −6.8478 kJ.mol^−1^, **Blue**), **3** (E = −6.8610 kJ.mol^−1^, **Green**), **10b** (E = −7.0198 kJ.mol^−1^, **Red**), and **11b** (E = −7.5122 kJ.mol^−1^, **Magenta**). Analogues do not exhibit altered binding modalities with orientation within the pocket persistent in each analogue.

The possibility that the enhanced AhR upregulation noted with **11** (relative to the other analogues screened) is due to enhanced binding to the AhR, was investigated in a molecular docking study using our in-house AhR homology model. Our AhR model is based on the published PAS-B domain proteins, PDB 4F3L, 3RTY and 2KDK (Baker et al., 2018; Baker et al., 2021a). AhR upregulation may be a function of increased engagement of **11** with the active site. As previously described (Baker et al., 2018; Baker et al., 2021a), analogues **3**, **5**, **10b**, and **11b** were docked within the AhR active site using the Molecular Operating Environment (MOE) software suite (Chemical Computing Group, Montreal, Canada). On docking of each analogue, 500-poses were generated, and their respective docking energies minimised with the top-10 (based on predicted binding energies) examined in greater detail. The outcome of this examination is shown in Figure 6.

Examination of the stacked pose snapshot of the top binding poses of all screened analogues (Figure 6) revealed limited deviation in docked binding poses, binding energy, and binding location of the analogues (**3**, **5**, **10b** and **11b**) examined. There were no observable changes in analogue binding. The introduction of a C-10 disposed halogen had no effect on binding energies (**3**, −6.8610 kJ.mol^-1^ vs. **5**, -6.8478 kJ.mol^-1^). C-4 derivatisation resulted in a slight increase in binding efficiency (**10b**, −7.0198 kJ.mol^-1^ and **11b**, −7.5122 kJ.mol^-1^), however no further interactions were observed toward the -Cl or -NO_2_ moieties. This suggests that it is highly unlikely that the observed increase to AhR upregulation is due to increased or altered binding to the AhR.

**FIGURE 7:**
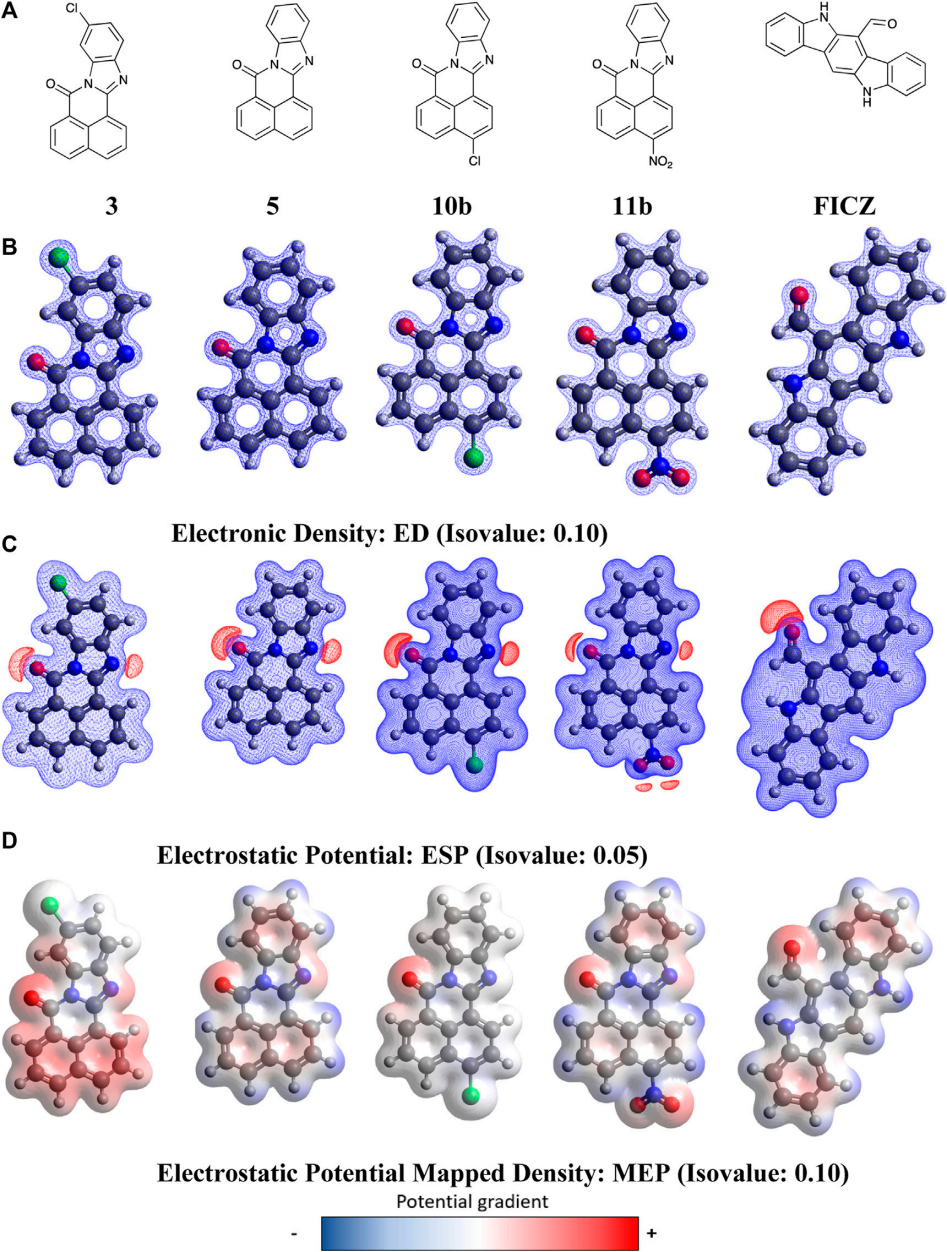
DFT optimised geometry and electronic effects plots for **(A)** BBQ analogues **3**, **5**, **10b**, **11b** and **FIZC**; **(B)** electronic distribution for compounds in ‘A’ displayed at a 0.1 isovalue; **(C)** electrostatic potential maps for compounds in ‘**A**’ displayed at 0.05 isovalue; **(D)** electronic potential mapped density for compounds in ‘**A**’ displayed at 0.05 isovalue. Isovalue choice ( $\frac{e^{-}}{{Å}^{3}}$ )was guided by literature and is kept consistent between analogues. Volumetric surfaces are coloured on a gradient with regions of negative potential (repulsion with respect to a positive charge) shown in blue and regions of positive potential (attraction with respect to a positive charge) shown in red.

With molecular modelling revealing no key analogue-AhR active site docking differences that explained the increased AhR activation by **11**, we conducted higher level (DFT) calculations to explore the possible impact of compound geometry and electronic effects. The endogenous AHR ligand, FICZ (6-formylindolo(3,2-*b*)carbazole) was also examined to highlight key regions that are conserved between species. DFT calculations were performed using the dispersion-corrected (D3BJ), spin-component scaled double hybrid functional DSD-PBEPB86 developed by Kozuch and Martin (Grimme et al., 2010; 2011; Goerigk and Grimme, 2011; Goerigk et al., 2017) in conjunction with the valence triple-zeta with additional polarisation and diffuse functions basis set def2-TZVPP of Ahlrichs (Weigend and Ahlrichs, 2005; Hellweg et al., 2007; Jensen, 2013). Functional choice was guided by literature, showing improved accuracy over other common hybrid functionals such as B3LYP or B2PLYP (Kozuch and Martin, 2011; Karton and Spackman, 2021). Geometry optimisation calculations were performed in ORCA version 5.1 (Neese, 2012; 2018) and relevant plots constructed in Avogadro version 1.20 (Hanwell et al., 2012; Avogadro, 2022). These studies commenced with the calculation and visualisation of electrostatic potential (ESP), electron density (ED), and electrostatic potential mapped density (MEP) plots for **3**, **5**, **10**, **11**, and FICZ (Figure 7, Electronic Supplementary Material).

Analysis of the data presented in Figure 7 reveals that the BBQ analogues (**3**, **5**, **10b** and **11b**) share essentially identical geometries and electronic distributions governed by the parent BBQ scaffold (**5**). Modification of parent **5**, with the C-4 substituted **10b** and **11b** is accompanied by a decrease to positive potential in the naphthyl region of these analogues, as indicated by a colour mapping shift (red to blue) (Figure 7). Conversely, the C-10 dispose chloro-**3** results in activation of this naphthyl region towards π−π type interactions (blue to red) (Figure 7). The van der Waal radii remains similar in all analogues with a slight increase observed in the NO_2_ containing analogue **11b**. All analogues, including FICZ, show remarkably similar regions of positive potential as indicated by the electrostatic potential map (ESP) map, localised around the carbonyl and imine (surface shown in red) (Figure 7). Our initial docking studies suggested that the imide carbonyls were not required for activity (contrary to what we have shown in related studies) (Baker et al., 2021a), however their inclusion aligns well to the endogenous ligand FICZ. While detailing the nature of BBQ analogues geometry and electronic distribution, this analysis is not consistent with the observed activation of the AhR by **11**. As the interplay between AhR, CYP and SULT1A1 is crucial for activity, we hypothesised that the **11**-mediated AhR activity may be a function not of **11**, but of a CYP activated **11**-metabolite.

### 2.7 Use of Fukui Functions to predict CYP1A1 mediated metabolites

The cytotoxicity of BBQ analogues is dependent on CYP1A1 and subsequent SULT1A1 metabolism (Figures 4, 5) to form the sulfonic ester warhead. CYP1A1 is capable of nucleophilic, electrophilic and single electron transfer (SET) oxidation initiating a metabolic process designed to solubilise and ultimately excrete ligands translocated by the AHR. The CYP1A1 mediated oxidation of FICZ is well studied (Wei et al., 1998; Wincent et al., 2009; Wincent et al., 2012), with monosubstituted 2-, and 8- OH analogues as the primary metabolites. Sequential oxidation produces the di-substituted 2-/8-, 2-/10- and 4-/8- diOH metabolites. However, the exact CYP1A1-FICZ hydroxylation mechanism remains elusive (Meunier et al., 2004; Guengerich, 2018). To examine the potential sites of CYP1A1 oxidation, via DFT calculations, we looked to calculate Fukui functions (indices) for these BBQ analogues (Proft et al., 2002; Pucci and Angilella, 2022). Specifically, using condensed Fukui Functions, permits calculation of the change in electron density when an electron is removed (mimicking electrophilic attack at that atom), added (mimicking nucleophilic attack at that atom) to a molecule or if a site is likely to undergo a SET reaction. These are the known reactions of CYP1A1, and related cytochrome P450 enzymes (Beck, 2005). This allows determination of the pseudo-probability of attack at a specific atom within a molecule (Electronic Supplementary Material).

Commencing with FICZ, Fukui Functions were calculated to predict the potential CYP1A1 metabolite(s). In analysing the Fukui Function data output, transformations that are unlikely to occur (nucleophilic attack towards the carbonyl oxygen or nitrogen atom, or addition to a quaternary carbon or loss of aromaticity) were considered non-viable options for metabolite generation. Additional consideration was given to the size and shape of the CYP1A1 catalytic site (Electronic Supplementary Material), it is likely that FICZ and other analogues are too large to pass through with their largest vDW radii parallel to the metabolic site which invalidates them as reactive sites. Hirshfeld population analysis was utilised for generation of Fukui indices (for complete numerical Fukui indices, see Electronic Supplementary Material) due to it its lower reliance on basis set choice (North et al., 2023).

**FIGURE 8:**
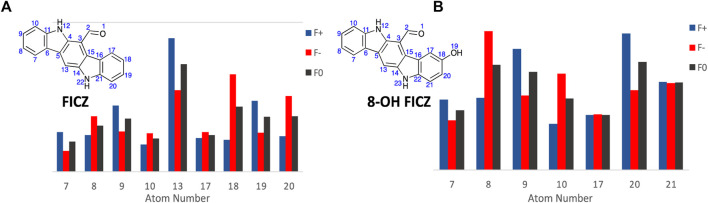
Condensed Fukui Function data generated in ORCA (DSD-PBEP86/def2-TZVPP), with the output graphed Hirshfeld population probabilities, and the chemical structures of, **(A)**. FICZ; and **(B)**. 8-OH FIZC are shown. Colour legend for the numerical results (Hirshfeld population) for the *f* ^*+*^ function showing regions likely to undergo nucleophilic attack (**blue**), *f* ^*-*^ function showing regions more probable to undergo electrophilic attack (**red**), and the *f* ^*0*^ function showing regions more probable to undergo SET reactions (**black**).

The primary CYP1A1 metabolites of FICZ are known to be the corresponding 2- and 8-OH analogues (atoms 9 and 18, A_9_ and A_18_ respectively). However initial analysis of the Fukui Function probability plots, Figure 8, highlights atom 13 (A_13_) with the largest values for both *f* ^*-*^ and *f* ^*0*^, indicating a propensity to undergo electrophilic or SET attack at this atom. However, the size and binding characteristics of the CYP1A1 active precludes this possibility. Within the remaining FICZ atoms, A_18_ displays the next largest *f* ^−^ (0.06529) and *f* ^*0*^ (0.04334) function values (Figure 8A), and thus a high probability of electrophilic or SET attack at this atom. This corresponds to the 8-OH FICZ metabolite, mechanistically suggesting its generation from FICZ by CYP1A1 occurs via electrophilic or SET attack. With a known second CYP1A1 mediated oxidation of FICZ (and thus most likely 8-OH FICZ), we generated Fukui Functions arising from the potential second pass oxidation of 8-OH FIZC (Figure 8B). From this, A_8_ has the highest probability of participating in electrophilic or SET attack. This, again, corresponds well with the known second generation FICZ metabolism products, the 2,8-di-OH FICZ analogues (Wincent et al., 2009). Within the limitations of the Fukui Functions, i.e., an ability to predict the likelihood of an atom to accept or donate an electron. This is not the ability to predict that the reaction will occur (Pucci and Angilella, 2022; Zaklika et al., 2022).

**FIGURE 9:**
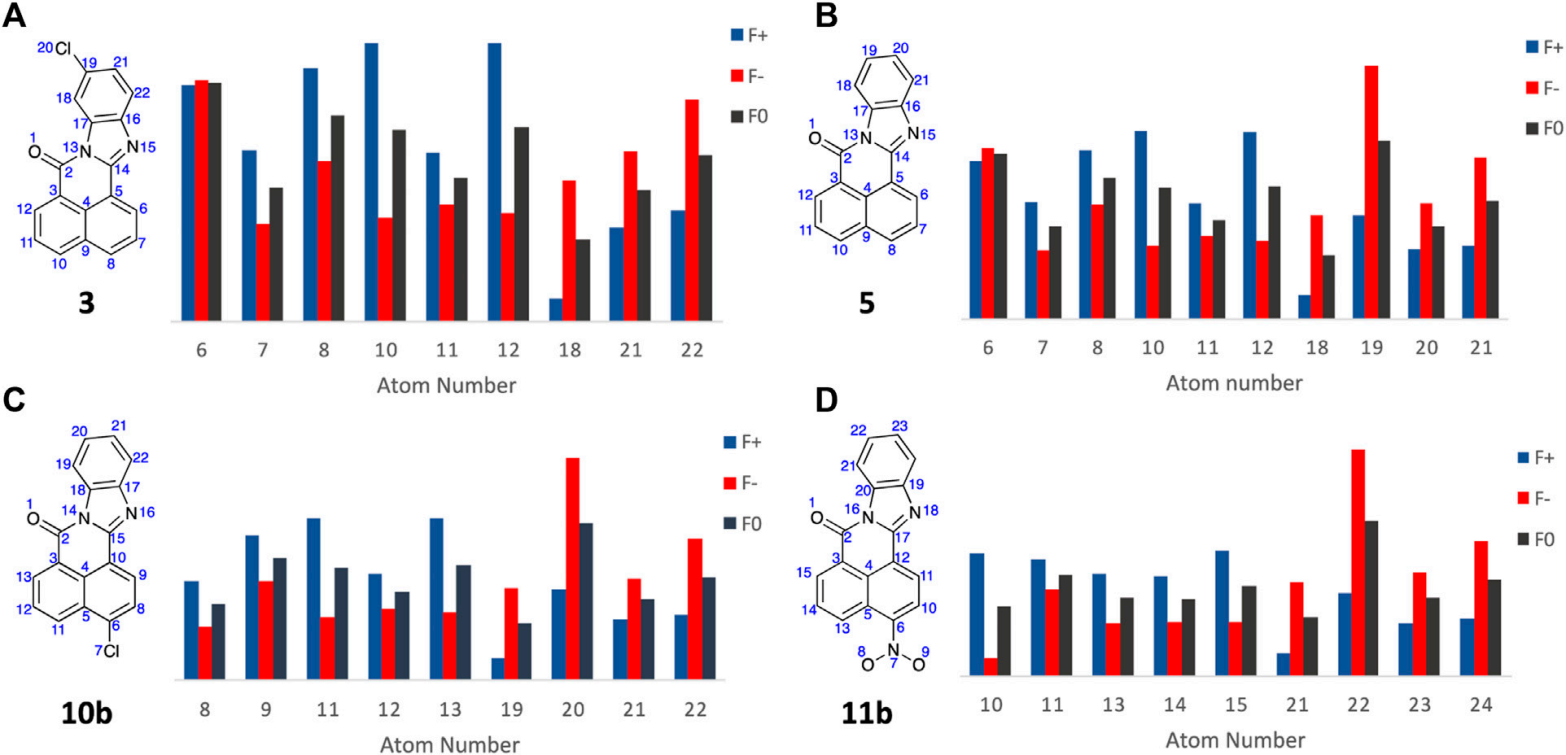
Condensed Fukui Function data generated in ORCA (DSD-PBEP86/def2-TZVPP), with the output graphed Hirshfeld population probabilities and displaying the chemical structures of the BBQ analogues examined, for **(A) 3**; **(B) 5**; **(C) 10b**; and **(D) 11b**. Colour legend for the numerical results (Hirshfeld population) for the *f* ^*+*^ function showing regions likely to undergo nucleophilic attack (**blue**), *f* ^*-*^ function showing regions more probable to undergo electrophilic attack (**red**), and the *f* ^*0*^ function showing regions more probable to undergo SET reactions (**black**).

With the Fukui Functions predicting the known metabolites of FICZ, we recapitulated this study with BBQ analogues **3**, **5**, **10b** and **11b** (Figure 9) in an effort to correlate Fukui Function identified metabolites trends with the observed cytotoxicity (Table 1).

Analysis of the Fukui Function output (full details of the Fukui Functions of **3**, **5**, **10b** and **11b** first and second pass metabolites are supplied in the Electronic Supplementary Material) suggests that with analogue **3**, A_6_ is most likely to undergo electrophilic and SET attack to produce the 4-OH analogue; while **5**, **10b** and **11b** all show high *f* ^*-*^ and *f* ^*0*^ values which predicts the production of the corresponding C10-OH analogues (A_19_, A_20_, and A_22_ respectively, Figure 9)). This is consistent with the observed cytotoxicity of these analogues, with **5**, **10** and **11** > 10 fold more potent than head group substituted analogue, c.f. **3**, **4**, **6**, **7**, **8**, and **9** across the cell lines examined (Table 1). The sub nanomolar activity of **11b** is likely a function of the *in situ* formation of the corresponding nitrenium ion, through a nitroso intermediate (Uetrecht et al., 1997; Sidorenko et al., 2014; Ergüç et al., 2023).

## 3 Discussion

The finding that certain compounds (ANI-7 (**1**), NAP-6 (**2**), aminoflavone) to induce growth inhibition in a select panel of breast cancer cell lines and other ER positive cell lines (i.e., A431 skin and H460 lung), while having minimal to no effect in cell lines derived from other tumour types has previously been presented by us and others. This includes overcoming the resistance of MDA-MB-231 cells to such treatment (Meng et al., 2005; Bradshaw et al., 2008; Zhang et al., 2009; Callero and Loaiza-Pérez, 2011; Brinkman et al., 2014; Fukasawa et al., 2015; Gilbert et al., 2017; 2020; Baker et al., 2018). Subsequent evaluation has identified the bio-activation of these compounds via AhR induced CYP1 expression, leading to phase I metabolic conversion followed by SULT1A1 phase II activation, culminating in DNA damage and cell death (Glatt, 1997). The unique ability of such compounds relies on a cascade of chemical and biological events, i.e., each compound needs to (i) have chemical features to support AhR ligand binding and CYP expression, (ii) be a good substrate for CYP1 enzymatic activity, (iii) produce a product following CYP1 action that is a good substrate for SULT1A1 enzymatic activity, and (iv) finally convert to an unstable electrophile that supports DNA binding and cell death. While a plethora of exogenous and endogenous compounds can activate the AhR (Chong et al., 2023), their chemical structure dictates the cascade of events, i.e., dioxin (2,3,7,8-tetrachlorodibenzo-*p*-dioxin) activates AhR and CYP1 activity but is not a substrate for CYP1 activity, and fails to undergo metabolic conversion (Inui et al., 2014). With the right chemical composition breast cancers become vulnerable to treatment because they not only have a hyper-active AhR pathway (Vacher et al., 2018), as a likely legacy of exposure to environmental carcinogens, but they also have inherently high SULT1A1 activity (Mercer et al., 2009; Huang et al., 2014), due to its role in the metabolism of estrogens and environmental compounds (Mercer et al., 2009; Jiang et al., 2010). Indeed, SULT1A1 expression has been proposed to be a biomarker for this treatment approach (Huang et al., 2014).

Herein we present a small chemical library of analogues based upon the chemical structure of 10-chloro-7*H*-benzo[*de*]benzo[4,5]imidazo[2,1-*a*]isoquinolin-7-one (**3**, 10-Cl BBQ), a compound previously identified as activating the AhR pathway and presenting with selective growth inhibition in cells of breast cancer origin (Baker et al., 2021a; Elson et al., 2023). The library included analogues with modifications to the phenyl ring (**4–9**), imidazole ring (**13**), and modifications to the naphthyl rings (**10**-**14**). Collectively, analogues that contained substituents on the phenyl ring (**4** and **6**-**9**) including our lead (**3**) or alterations to the imidazole (**13**) ring presented with low to moderate growth inhibition (Table 1), suggesting that phenyl ring substituents hinder their biological action. This was further exemplified by analogue **5**, the simplest BBQ compound that lacked a phenyl ring substituent, but which induced strong growth inhibition (GI_50_ 0.001 μM) and 3200-fold selectivity in MDA-MB-468 cells compared with normal breast cells, also previously reported (Elson et al., 2023). In contrast, analogues containing moieties in the naphthyl rings maintained (**10**) or enhanced growth inhibition potency (**11**). The latter, however, produced a response that showed very strong activity in the breast cell lines but also strong activity in other cell types, suggesting an additional mode-of-action to that observed for the other analogues. The mechanism of which is under further investigation.

Additional biological analysis confirmed that while analogues **3** and **5** differed in their growth inhibition potency, they both induce AhR activity in a reporter assay (Figure 3) and significantly induce downstream CYP1A1 and CYP1B1 gene expression (Figure 4). Supporting the proposal that the reduced activity of **3** compared with **5** was caused by a hindrance in the subsequent bio-activation rather than the inability to activate the AhR and its downstream target genes. Further biological analysis showed that the growth inhibition of **5** in MDA-MB-468 cells was dependent on CYP1 and SULT1A1 activity (Figure 5), as inhibitors of these enzymes substantially reduced its growth inhibitory action, again confirming the need to undergo bio-activation. Although, the metabolic products of our library were not identified experimentally in this study, their presence was strongly supported by calculation of the likely metabolites, consistent previous studies with aminoflavone and its analogues were shown to be dependent upon hydroxylation of its phenyl ring by CYPs, followed by sulfation of the same hydroxyl moiety by SULT1A1 (Meng et al., 2006). Indeed, the cell lines most sensitive to the BBQ analogues overexpress SULT1A1 (Gilbert et al., 2017), mimicking the response to aminoflavone (Meng et al., 2006; Gilbert et al., 2017), ANI-7 (**1**) (Gilbert et al., 2017) and NAP-6 (**2**) (Gilbert et al., 2020).

Traditional molecular modelling and DFT (DSD-PBEP86/TZVPP level of theory) approaches failed to reveal any significant geometrical, electronic or binding differences that explained the observed differences in cytotoxicity with **11** and **5**. Extending our computational approach to an understanding of CYP1A1 mediated metabolite generation, based on the activation cascade required for AhR ligands to display a cytotoxic outcome, we explored Fukui Functions. This computational approach successfully predicted the known first and second pass metabolites of FIZC (Figure 8). It was subsequently applied to BBQ analogues, where we noted that analogues with excellent cytotoxicity were liable to undergo hydroxylation at the C-10 position (**5**, **10**, **11**) (Figure 9). With the related 10- and 11- chloro BBQ analogues, **3** and **4**, hydroxylation is more likely to occur at the C-4 position, which translated to lower levels of cytotoxicity (Table 1). This outcome suggests that for high levels of cytotoxicity, that BBQ analogues should lack a C-10 disposed substituent.

Collectively, this body of work builds upon our understanding of the AhR pathway in breast cancer and the chemical structures required to develop novel agents that undergo bio-activation to a cancer killing molecule in a select population of cells, while having little to no effect in other cell types including normal breast cells. This phenomenon is unique and resides in the role of the AhR pathway in the initiation and progression of breast cancer (Glatt, 1997; Nebert et al., 2004; Mercer et al., 2009; Inui et al., 2014; Vacher et al., 2018). The metabolism of environmental toxins by the AhR lends credence to the initiation of breast cancer by a fat-soluble xenobiotic element that was metabolized to a DNA-damaging compound. The ongoing hyper-activation of the AhR in breast cancers and its ability to control many oncogenic pathways builds upon the role of AhR in progression of this disease. The hyper-expression of SULT1A1 in breast cancer due to its role in the metabolism of environmental toxins and estrogens, provides the perfect conditions for the development of unique breast cancer targeting molecules. Moreover, the application of Fukui Functions represents a time and cost-effective way of examining potential CYP metabolism outcomes, which in this case goes some way to explain the excellent activity of these BBQ analogues, and the high activity of **11**.

## 4 Experimental

### 4.1 General Chemistry methods

All reactions were performed using standard laboratory equipment and glassware. Reagents and solvents were purchased from Sigma Aldrich, or AK Scientific and used as received. Organic solvents were of reagent quality and used as received. Melting points were recorded in open capillaries on a Büchi Melting Point M-565. Where available, literature values are provided and appropriately referenced. Electrospray ionization (ESI) mass spectra and HPLC spectra were recorded on an Agilent Technologies 1,260 Infinity UPLC system with a 6120 Quadrupole LC/MS in ESI positive (ESI^+^) and negative (ESI^−^) modes. Zorbax SB-C_18_ Rapid Resolution HT 2.1 × 50 mm 1.8-Micron column, using a ratio of 1:4 10% HPLC-grade acetonitrile (ACN)/milli-Q H_2_O and HPLC-grade ACN (both with 0.1% formic acid) as carrier solvents. Thin-layer chromatography (TLC) was performed on Merck silica gel 60 F254 pre-coated aluminium plates with a thickness of 0.2 mm and retention factors (r_f_) determined where required. Column chromatography was performed under ‘flash’ conditions on Merck silica gel 60 (230–400 mesh).

Nuclear magnetic resonance (NMR) spectroscopy was performed on a Brüker Avance III 400 MHz spectrometer, where proton NMR (^1^H NMR) spectra and carbon NMR (^13^C NMR) spectra were acquired at 400 and 100 MHz respectively, or a Brüker Avance III 600 MHz spectrometer, where ^1^H NMR spectra and ^13^C NMR spectra were acquired at 600 and 150 MHz respectively. All spectra were recorded in deuterated dimethyl sulfoxide (DMSO-*d*_*6*_); or deuterated trifluoroacetic acid (TFA-*d*) obtained from Cambridge Isotope Laboratories Inc. Chemical shifts (δ) were measured in parts per million (ppm) and referenced against the internal standard and solvent peaks. Coupling constants (*J*) were measured in Hertz (Hz). NMR assignments were determined through the interpretation of one- and two-dimensional spectra. Multiplicities are denoted as singlet (s), doublet (d), doublet of doublets (dd), and multiplet (m). Peaks are listed in decreasing chemical shift in the following format: chemical shift (multiplicity, coupling constant, integration). Infrared spectroscopy (IR) were recorded on a Perkin Elmer Spectrum 2 FT-IR spectrometer.

### 4.2 Chemical synthesis

#### 4.2.1 10-Chloro-7H-benzo[de]benzo[4,5]imidazo[2,1-a]isoquinolin-7-one (3)

1,8-Naphthalic anhydride (1.0 eq., 198 mg, 1 mmol) was combined with 4-chloro-2-nitroaniline (1.5 eq., 259 mg, 1.5 mmol), iron powder (7.5 eq., 420 mg, 7.5 mmol) and glacial acetic acid (15 mL) and heated to reflux (120 °C). Left to heat for 18 h. The reaction was cooled to ambient temperature, was diluted with ethyl acetate (30 mL) and neutralised slowly with sat. NaHCO_3_ to ∼ pH 7. The organic mixture was then washed with water (2 × 30 mL), dried over magnesium sulfate (MgSO_4_) and concentrated under reduced pressure. The resulting mustard yellow solid was recrystallised from dimethylformamide (DMF) and filtered under vacuum. Left to dry under vacuum overnight, and the desired product was afforded as a bright yellow solid (133 mg, 64%). The resultant material was purified by column chromatography (0%–10% methanol (MeOH):dichloromethane (DCM)) to afford both the 10-Cl (r_f_: 0.74, 10% MeOH in DCM) and 11-Cl (r_f_: 0.76, 10% MeOH in DCM) isomers in separate fractions. The desired 10-Cl product was afforded as a bright yellow solid (36.6 mg, 27%), m.p.: 228°C–231 °C. ^1^H NMR (600 MHz, DMSO-*d*_*6*_) δ 8.73 (d, *J* = 7.2 Hz, 1H), 8.69 (dd, *J* = 7.2, 0.8 Hz, 1H), 8.54 (d, *J* = 7.8 Hz, 1H), 8.39 (t, *J* = 8.0 Hz, 2H), 7.97–7.88 (m, 3H), 7.51 (dd, *J* = 8.5, 2.0 Hz, 1H); ^13^C NMR (150 MHz, DMSO-*d*_*6*_) δ 160.2, 150.7, 144.6, 135.9, 132.8, 132.0, 131.6, 130.5, 129.7, 127.6, 127.4, 127.2, 126.6, 125.0, 122.6, 119.9, 119.4, 116.4. IR (neat): υ_max_ (cm^-1^) = 3,054 (arom. C-H), 1,695 (C=O), 771 (C-Cl); LRMS: (ESI^+^) m/z: 305 (C_18_H_10_^35^ClN_2_O) [M + H, 100%], 307 (C_18_H_10_^37^ClN_2_O) [M + H, 35%]. HPLC: Peak retention time: 1.272 min.

#### 4.2.2 11-Chloro-7H-benzo[de]benzo[4,5]imidazo[2,1-a]isoquinolin-7-one (4)

Purified by column chromatography with compound **5**. The desired 11-Cl product was afforded as a bright yellow solid (29.8 mg, 23%), m.p.: 234°C–236 °C. ^1^H NMR (600 MHz, DMSO-*d*_*6*_) δ 8.76 (dd, *J* = 7.2, 1.0 Hz, 1H), 8.71 (dd, *J* = 7.2, 1.0 Hz, 1H), 8.55 (d, *J* = 7.6 Hz, 1H), 8.415 (d, *J* = 7.8 Hz, 1H), 8.407 (d, *J* = 8.4 Hz, 1H), 7.99–7.90 (m, 3H), 7.52 (dd, *J* = 8.6, 2.0 Hz, 1H); ^13^C NMR (150 MHz, DMSO-*d*_*6*_) δ 160.2, 150.7, 144.6, 135.9, 132.8, 132.0, 131.6, 130.5, 129.8, 127.6, 127.4, 127.2, 126.6, 125.0, 122.6, 119.9, 119.4, 116.4. IR (neat): υ_max_ (cm^-1^) = 3,055 (arom. C-H), 1,695 (C=O), 774 (C-Cl); LRMS: (ESI^+^) m/z: 305 (C_18_H_10_^35^ClN_2_O) [M + H, 100%], 307 (C_18_H_10_^37^ClN_2_O) [M + H, 35%]. HPLC: Peak retention time: 1.304 min.

#### 4.2.3 7H-Benzo[de]benzo[4,5]imidazo[2,1-a]isoquinolin-7-one (5)

1,8-Naphthalic anhydride (1.0 eq., 198 mg, 1.0 mmol) was combined with 1,2-phenylenediamine (1.5 eq., 169 μL, 1.5 mmol) and glacial acetic acid (15 mL) and heated to reflux for 4 h. The reaction was cooled to ambient temperature, and a yellow/orange solid was isolated via vacuum filtration. The resulting solid was recrystallised from DMF, and the desired product was afforded as a yellow solid (193 mg, 70%), m.p.: 205°C–207 °C (lit. 204°C–209°C, Mamada et al., 2011). ^1^H NMR (600 MHz, DMSO-*d*_*6*_) δ 8.73 (d, *J* = 7.2 Hz, 1H), 8.68 (d, *J* = 7.2 Hz, 1H), 8.51 (d, *J* = 8.2 Hz, 1H), 8.44–8.41 (m, 1H), 8.37 (d, *J* = 8.2 Hz, 1H), 7.94–7.86 (m, 3H), 7.51–7.48 (m, 2H); ^13^C NMR (150 MHz, DMSO-*d*_*6*_) δ 160.3, 149.1, 143.4, 135.6, 132.2, 131.9, 131.5, 131.3, 127.5, 127.2, 126.7, 126.5, 125.5, 125.1, 122.8, 120.2, 119.8, 115.3; IR (neat): υ_max_ (cm-1) = 3,387 (arom. C-H), 3,066 (arom. C-H), 1,690 (C=O); LRMS: (ESI^+^) m/z: 271 (C_18_H_11_N_2_O) [M + H, 100%].

#### 4.2.4 10,12-Dichloro-7H-benzo[de]benzo[4,5]imidazo[2,1-a]isoquinolin-7-one (6)

1,8-Naphthalic anhydride (1.0 eq., 156 mg, 0.8 mmol) was combined with 2,4-dichloro-6-nitroaniline (1.5 eq., 263 mg, 1.2 mmol), iron powder (7.5 eq., 330 mg, 5.9 mmol) and glacial acetic acid (10 mL) and heated to reflux (120 °C). Left to heat for 18 h, then cooled to ambient temperature. The reaction was diluted with ethyl acetate (30 mL) and neutralised slowly with sat. NaHCO_3_ to ∼ pH 7. The organic mixture was then washed with water (2 × 30 mL), dried over MgSO_4_ and concentrated under reduced pressure. The resulting yellow solid was purified by column chromatography (0%–10% MeOH:DCM) to afford the desired product as a yellow solid (69 mg, 25%), m.p.: 292°C–294 °C. ^1^H NMR (400 MHz, TFA-*d*) δ 9.13 (d, *J* = 7.4 Hz, 1H), 9.10 (d, *J* = 7.4 Hz, 1H), 8.80 (d, *J* = 1.7 Hz, 1H), 8.63 (d, *J* = 5.4 Hz, 1H), 8.61 (d, *J* = 5.4 Hz, 1H), 8.08 (t, *J* = 6.9 Hz, 1H), (t, *J* = 6.9 Hz, 1H), 7.79 (d, *J* = 1.7 Hz, 1H); ^13^C NMR (100 MHz, TFA-*d*) δ 161.8, 151.9, 142.3, 141.9, 139.1, 138.9, 135.2, 135.1, 132.6, 132.4, 131.4, 130.7, 130.0, 129.6, 123.1, 122.7, 118.6, 114.1; IR (neat): υ_max_ (cm^-1^) = 3,106 (arom. C-H), 3,091 (arom. C-H), 1700 (C=O); LRMS: (ESI^+^) m/z: 339 (C_18_H_9_^35^Cl_2_N_2_O) [M + H, 100%]; 341 (C_18_H_9_^35^Cl^37^ClN_2_O) [M + H, 70%]; HPLC: Peak retention time: 2.677 min.

#### 4.2.5 10-Methyl-7H-benzo[de]benzo[4,5]imidazo[2,1-a]isoquinolin-7-one (7a) and 11-methyl-7H-benzo[de]benzo[4,5]imidazo[2,1-a]isoquinolin-7-one (7b)

1,8-Naphthalic anhydride (1.0 eq., 198 mg, 1 mmol) was combined with 3,4-diaminotoluene (1.5 eq., 196 mg, 1.5 mmol) and glacial acetic acid (15 mL) and heated to reflux for 18 h, then cooled to ambient temperature. The reaction was diluted with ethyl acetate (30 mL) and neutralised slowly with sat. NaHCO_3_ to ∼ pH 7. The organic mixture was then washed with water (2 × 30 mL), dried over MgSO_4_ and concentrated under reduced pressure. The resulting mustard yellow solid was recrystallised from DMF and filtered under vacuum. Left to dry under vacuum overnight, and the desired product was afforded as a mustard yellow solid (182 mg, 64%), m.p.: 192°C–194 °C. ^1^H NMR (600 MHz, DMSO-*d*_*6*_) δ 8.73 (dd, *J* = 7.2, 0.9 Hz, 1.3H, **7b**), 8.71 (dd, *J* = 7.3, 0.9 Hz, 1H, **7a**), 8.69 (t, *J* = 1.1 Hz, 1H, **7a**), 8.68 (t, *J* = 1.1 Hz, 1.3H, **7b**), 8.53–8.52 (m, 2.3H, **7a**, **7b**), 8.38–8.36 (m, 2.3H, **7a**, **7b**), 8.29 (d, *J* = 8.2 Hz, 1.2H, **7b**), 8.25 (s, 1H, **7a**), 7.96–7.93 (m, 2.3H, **7a**, **7b**), 7.92–7.89 (m, 2.3H, **7a**, **7b**), 7.74 (d, *J* = 8.2 Hz, 1H, **7a**), 7.66 (s, 1.3H, **7b**), 7.31 (dd, *J* = 3.5, 1.0 Hz, 1.3H, **7b**), 7.30 (dd, *J* = 3.5, 1.0 Hz, 1H, **7a**), 2.53 (s, 3H, **7a**), 2.48 (s, 3.9H, **7b**); ^13^C NMR (150 MHz, DMSO-*d*_*6*_) δ 160.3 (**7b**), 160.1 (**7a**), 149.2 (**7b**), 148.7 (**7a**), 143.8 (**7b**), 141.5 (**7a**), 135.58 (**7b**), 135.57 (**7a**), 135.0 (**7b**), 134.9 (**7a**), 132.1 (2 overlapping signals, **7a**, **7b**), 131.98 (7b), 131.96 (2 overlapping signals, 7a, 7b), 131.7 (7a), 131.24 (7b), 131.19 (**7a**) 129.5 (2 overlapping signals, **7a**, **7b**), 127.6 (2 overlapping signals, **7a**, **7b**), 127.24 (**7b**), 127.23 (**7a**), 126.8 (**7a**), 126.7 (**7b**), 126.5 (2 overlapping signals, 7a, 7b), 126.4 (**7a**), 126.3 (**7b**), 122.84 (**7a**), 122.81 (**7b**), 120.3 (**7a**), 120.2 (**7b**), 119.7 (**7b**), 119.3 (**7a**), 21.5 (**7a**), 21.3 (**7b**); Ratio **7a**: **7b** = 1 : 1.3. IR (neat): υ_max_ (cm^-1^) = 3,066 (arom. C-H), 2,197 (arom. C-H), 1,692 (C=O); LRMS: (ESI^+^) m/z: 285 (C_19_H_12_N_2_O) [M + H, 100%], HPLC: Peak retention time: 2.724 (**7a**, **7b**) min.

#### 4.2.6 9-Methyl-7H-benzo[de]benzo[4,5]imidazo[2,1-a]isoquinolin-7-one (8a) and 12-methyl-7H-benzo[de]benzo[4,5]imidazo[2,1-a]isoquinolin-7-one (8b)

1,8-Naphthalic anhydride (1.0 eq., 234 mg, 1.2 mmol) was combined with 2-methyl-6-nitroaniline (1.5 eq., 270 mg, 1.8 mmol), iron powder (7.5 eq., 494 mg, 8.9 mmol) and glacial acetic acid (15 mL) and heated to reflux for 18 h, then cooled to ambient temperature. The reaction was diluted with ethyl acetate (30 mL) and neutralised slowly with sat. NaHCO_3_ to ∼ pH 7. The organic mixture was then washed with water (2 × 30 mL), dried over MgSO_4_ and concentrated under reduced pressure. The resulting mustard yellow solid was recrystallised from DMF and filtered under vacuum. Left to dry under vacuum overnight, and the desired product was afforded as a bright yellow solid (130 mg, 39%), m.p.: 252°C–254 °C. ^1^H NMR (400 MHz, TFA-*d*) δ 9.09 (2 overlapping doublets, *J* = 7.2 Hz, 1.2H, **8a**, **8b**), 9.06–9.04 (m, 1.2H, **8a**, 8b), 8.92–8.90 (m, 0.2H, **8a**), 8.68–8.66 (m, 1H, **8b**), 8.61–8.58 (m, 2.4H, 2H-**8a**, 2H-**8b**), 8.09–8.01 (m, 2.4H, 2H-**8a**, 2H-**8b**), 7.73–7.69 (m, 1.2H, **8a**, **8b**), 7.62–7.60 (m, 1.2H, **8a**, **8b**), 3.04 (s, 0.5H, **8a**), 2.74 (s, 3H, **8b**); ^13^C NMR (100 MHz, TFA-*d*) δ 162.4 (**8b**), 149.8 (**8b**), 141.2 (**8b**), 140.9 (**8b**), 140.3, 140.2, 138.3 (**8b**), 138.1, 137.73, 134.70 (**8b**), 133.7 (**8b**), 132.8, 132.6 (**8b**), 131.82, 131.76 (**8b**), 131.4 (**8b**), 130.9 (**8b**), 130.8 (**8b**), 130.1 (**8b**), 130.0, 129.0 (**8b**), 127.4 (**8b**), 122.6 (**8b**), 116.8 (**8b**), 114.1 (**8b**), 24.6, 17.1 (**8b**) (not all minor isomer peaks observed); Ratio **8a**: **8b** = 0.2 : 1. IR (neat): υ_max_ (cm^-1^) = 3,054 (arom. C-H), 2,977 (arom. C-H), 1701 (C=O), 1,699 (C=O), 779 (C-Cl), 773 (C-Cl); LRMS: (ESI^+^) m/z: 285 (C_18_H_10_^35^ClN_2_O) [M + H, 100%]. HPLC: Peak retention times: 3.013 (**8a**, 15%), 3.339 (**8b**, 85%) min.

#### 4.2.7 7-Oxo-7H-benzo[de]benzo[4,5]imidazo[2,1-a]isoquinoline-10-carbonitrile (9a) and 7-oxo-7H-benzo[de]benzo[4,5]imidazo[2,1-a]isoquinoline-11-carbonitrile (9b)

1,8-Naphthalic anhydride (1.0 eq., 198 mg, 1.0 mmol) was combined with 3,4-diaminobenzonitrile (1.5 eq., 206 mg, 1.5 mmol) and glacial acetic acid (15 mL) and heated to reflux for 4 h. The reaction was cooled to ambient temperature, and a yellow/orange solid was isolated via vacuum filtration. The resulting olive green solid was recrystallised from DMF, and the desired product was afforded as a yellow solid (169 mg, 56%), m.p.: 251°C–253 °C. ^1^H NMR (600 MHz, DMSO-*d*_*6*_) δ 8.78–8.75 (m, 2.6H), 8.73–8.70 (m, 3.2H), 8.58–8.53 (m, 2.8H), 8.45–8.40 (m, 2.7H), 8.02 (d, *J* = 8.3 Hz, 1.3H), 7.98–7.87 (m, 6.2H); ^13^C NMR (150 MHz, DMSO-*d*_*6*_) δ (160.3, 160.2), (152.3, 151.4), (146.5, 143.2), (136.14, 136.05), 134.6, (133.4, 133.1), (131.9, 131.3), (131.85, 131.73), (129.2, 128.5), (127.9, 127.6), (127.7, 127.52), (127.46, 127.39), 127.71, 124.2, (122.5, 122.4), 120.9, (119.6, 119.4), 119.28, (119.25, 119.23), 118.6, (116.4, 114.7), (107.7, 106.5); Ratio **9a**: **9b** = 1 : 1.3. IR (neat): υ_max_ (cm^-1^) = 3,102 (arom. C-H), 2,221 (CN), 1,699 (C=O); LRMS: (ESI^+^) m/z: 296 (C_19_H_9_N_3_O) [M + H, 100%].

#### 4.2.8 3-Chloro-7H-benzo[de]benzo[4,5]imidazo[2,1-a]isoquinolin-7-one (10a) and 4-chloro-7H-benzo[de]benzo[4,5]imidazo[2,1-a]isoquinolin-7-one (10b)

4-Chloro-1,8-naphthalic anhydride (1.0 eq., 244 mg, 1.0 mmol) was combined with 2-aminobenzylamine (1.5 eq., 171 mg, 1.5 mmol) and glacial acetic acid (15 mL) and heated to reflux (120 °C). Left to heat for 4 h. The reaction was cooled to ambient temperature, diluted with water (10 mL), and a bright yellow solid was isolated via vacuum filtration. The resulting solid was recrystallised from DMF, and the desired product was afforded as a bright yellow solid (224 mg, 73%), m.p.: 222–225, 237°C–239 °C. ^1^H NMR (400 MHz, TFA-*d*) δ 9.12 (d, *J* = 7.5 Hz, 1H, **10b**), 9.06–8.98 (m, 3.3H, 10a, 2H-**10b**), 8.95 (d, *J* = 8.1 Hz, 1.3H, **10a**), 8.86 (d, *J* = 8.1 Hz, 1H, **10b**), 8.82–8.75 (m, 2.3H, **10a**, **10b**), 8.11 (m, 4.6H, 2H-10a, 2H-**10b**), 7.89 (dd, *J* = 8.3, 5.6 Hz, 2.3H, **10a**, **10b**), 7.79 (dd, *J* = 5.8, 3.2 Hz, 4.3H, 2H-**10a**, 2H-**10b**); ^13^C NMR (100 MHz, TFA-*d*) δ 161.7, 161.5, 149.6, 149.4, 147.5, 138.8, 137.9, 137.4, 137.3, 134.2, 133.3, 132.7, 132.6, 132.4, 132.3, 131.9, 131.8, 131.51, 131.47 (2 overlapping signals), 131.39, 131.0, 130.9, 130.81, 130.75, 130.0, 123.2, 121.4, 119.34, 119.31, 118.5, 117.4, 116.1, 116.0, 114.6, 112.9; Ratio **10a**: **10b** = 1.3 : 1. IR (neat): υ_max_ (cm^-1^) = 3,060 (arom. C-H), 1,696 (C=O), 743 (C-Cl); LRMS: (ESI^+^) m/z: 305 (C_18_H_10_^35^ClN_2_O) [M + H, 70%], 307 (C_18_H_10_^37^ClN_2_O) [M + H, 25%]. HPLC: Peak retention times: 4.273 (**10b**, 30%), 4.700 (**10a**, 70%) min.

#### 4.2.9 3-Nitro-7H-benzo[de]benzo[4,5]imidazo[2,1-a]isoquinolin-7-one (11a) and 4-nitro-7H-benzo[de]benzo[4,5]imidazo[2,1-a]isoquinolin-7-one (11b)

4-Nitro-1,8-naphthalic anhydride (1.0 eq., 249 mg, 1.0 mmol) was combined with 2-aminobenzylamine (1.5 eq., 691 mg, 1.5 mmol) and glacial acetic acid (15 mL) and heated to reflux (120 °C). Left to heat for 4 h. The reaction was cooled to ambient temperature, diluted with water (10 mL), and a bright yellow solid was isolated via vacuum filtration. The resulting solid was recrystallised from DMF, and the desired product was afforded as a bright yellow solid (224 mg, 71%), m.p.: 258–259, 265°C–267 °C. ^1^H NMR (400 MHz, TFA-*d*) δ 9.16–9.01 (m, 6H, 3H-11a, 3H-**11b**), 8.81–8.76 (m, 2H, **11a**, **11b**), 8.58–8.56 (m, 2H, 11a, 11b), 8.22–8.16 (m, 2H, 11a, 11b), 7.96–7.87 (m, 2H, **11a**, **11b**), 7.87–7.80 (m, 4H, 2H-11a, 2H-**11b**); ^1^3C NMR (100 MHz, TFA-*d*) δ 161.0, 160.1, 154.1, 153.8, 149.7, 148.0, 138.8, 136.5, 135.3, 135.2, 134.4, 133.3, 132.68, 132.66, 132.5, 132.4, 132.3, 132.19, 132.16, 132.0, 131.2, 131.0, 130.2, 130.0, 127.12, 127.06, 126.8, 126.64, 126.60, 123.5, 119.4, 119.3, 119.1, 116.6, 116.4, 115.3; Ratio **11a**: **11b** = 1 : 1. IR (neat): υ_max_ (cm^-1^) = 3,102 (arom. C-H), 3,066 (arom. C-H), 1704 (C=O), 1,699 (C=O), 1,520 (NO_2_), 1,519 (NO_2_) 1,327 (NO_2_), 1,323 (NO_2_); LRMS: (ESI^+^) m/z: 315 (C_18_H_9_N_3_O_3_) [M, 25%]. HPLC: Peak retention times: 2.502, 2.714 min.

#### 4.2.10 Benzo[4,5]imidazo[1,2-b]isoquinolin-11(5H)-one (12)

Homophthalic anhydride (1.0 eq., 324 mg, 2 mmol) was combined with 1,2-phenylenediamine (1.5 eq., 324 mg, 3 mmol) and glacial acetic acid (15 mL) and heated to reflux for 14 h. The reaction was cooled to ambient temperature, diluted with water (10 mL), and filtered under vacuum. The resulting olive green solid was taken up in diethyl ether (15 mL), sonicated, and filtered under vacuum. The desired product was afforded as an olive green solid (241 mg, 49%), m.p.: decomp >250 °C. ^1^H NMR (600 MHz, DMSO-*d*_*6*_) δ 11.32 (s, 1H), 8.74 (d, *J* = 8.0 Hz, 1H), 8.48 (d, *J* = 8.0 Hz, 1H), 7.50 (t, *J* = 7.5 Hz, 1H), 7.38 (t, J = 7.5 Hz, 1H), 7.31 (t, *J* = 7.5 Hz, 1H), 7.25 (t, *J* = 7.5 Hz, 1H), 7.16–7.14 (m, 2H); ^13^C NMR (150 MHz, DMSO-*d*_*6*_) δ 159.3, 141.4, 138.9, 133.4, 132.1, 128.6, 127.4, 126.0, 122.4, 121.5, 120.1, 118.5, 115.8, 109.2, 82.1; IR (neat): υ_max_ (cm^-1^) = 3,089 (N-H), 1,652 (C=O); LRMS: (ESI^+^) m/z: 235 (C_15_H_11_N_2_O) [M + H, 100%]; (ESI^−^) m/z: 233 (C_15_H_9_N_2_O) [M-H, 100%].

#### 4.2.11 7H,9H-benzo[4,5]isoquinolino[1,2-b]quinazolin-7-one (13)

1,8-Naphthalic anhydride (1.0 eq., 297 mg, 1.5 mmol) was combined with 2-aminobenzylamine (1.5 eq., 275 mg, 2.25 mmol) and glacial acetic acid (15 mL) and heated to reflux for 14 h. The reaction was cooled to ambient temperature, diluted with water (10 mL), and the solvent reduced by half under a flow of compressed air. A yellow/orange solid was isolated via vacuum filtration. The resulting solid was recrystallised from DMF, and the desired product was afforded as a pale yellow solid (253 mg, 59%), m.p.: 256°C–258 °C. ^1^H NMR (600 MHz, DMSO-*d*_*6*_) δ 8.51 (t, *J* = 7.6 Hz, 4H), 8.14 (t, *J* = 5.3 Hz, 1H), 7.90 (t, *J* = 7.5 Hz, 2H), 7.45–7.43 (m, 3H), 7.35 (d, *J* = 7.5 Hz, 1H), 4.14 (d, *J* = 5.8 Hz, 2H); ^13^C NMR (150 MHz, DMSO-*d*_*6*_) δ 168.5, 163.6, 137.2, 134.8, 134.4 (2C), 131.5, 130.6 (2C), 129.7, 129.2, 128.5, 128.1, 128.0, 127.1 (2C), 122.8, 38.7, 22.2; IR (neat): υ_max_ (cm^-1^) = 3,244 (arom. C-H), 3,066 (arom. C-H), 1,665 (C=O); LRMS: (ESI^+^) m/z: 345 (C_22_H_21_N_2_O_2_) [M + IsoProp + H, 100%]; 367 (C_17_H_19_N_4_O) [M+2(CH_3_CN))+H, 40%].

### 4.3 Molecular modelling

The structures of all ligands to be docked were constructed in MOE and their conformations energy-minimized using Molecular Mechanics in conjunction with the AMBER force field. Docking was performed using MOE’s default settings, using the triangle matcher method in combination with the London δG scoring function for the initial placement of the ligand, followed by a refinement using induced fit methods and the GBVI/WSA scoring function. Compounds were docked 500 times, and the top 15 poses subjected to energy minimisation after refinement. Analysis and visualization of the docking output, such as identification of hydrogen bonds, steric clashes, hydrophobic interactions, or π−π-interactions were performed in MOE. Protein crystal structures were protonated, and energy minimised prior to docking.

#### 4.3.1 Computational DFT methods

Molecules were built in Avogadro (v.1.2.0) and subjected to first pass, force-field, geometric optimisation. Geometric optimisations of the ground state molecules were performed in ORCA (v.5.1.0) at the DSD-PBEPB86/def2-TZVPP level of theory. The same level of theory was used to produce molecular electrostatic potential (MEP) and electrostatic potential (ESP) maps. Outputs were then imported into Avogadro and visualised at relevant a relevant and consistent isovalue. Hirshfeld charge densities were then calculated for the cationic and ionic species by introducing or removing an electron from the ground state optimised structure allowing for the calculation of Fukui functions for each of the assessed molecules. No conformational searches were conducted prior to DFT calculation due to the low number of available conformers of all screened molecules.

### 4.4 Cell culture and stock solutions

Stock solutions were prepared as follows and stored as 20 mM DMSO solutions at −20 °C. All cell lines were cultured in a humidified atmosphere 5% CO_2_ at 37 °C. The cancer cell lines were maintained in Dulbecco’s modified Eagle’s medium (DMEM) (Trace Biosciences, Australia) supplemented with 10% foetal bovine serum (FBS), 10 mM sodium bicarbonate, penicillin (100 IU/mL), streptomycin (100 μg/mL), and glutamine (4 mM). The non-cancer MCF10A cell line was cultured in DMEM:F12 (1:1) cell culture media, 5% heat inactivated horse serum, supplemented with penicillin (50 IU/mL), streptomycin (50 μg/mL), 20 mM Hepes, L-glutamine (2 mM), epidermal growth factor (20 ng/mL), hydrocortisone (500 ng/mL), cholera toxin (100 ng/mL), and insulin (10 μg/mL). The AhR reporter cell line HT29-Lucia™ (Invivogen, United States) was maintained in DMEM supplemented with 10% heat inactivated FBS, L-glutamine (2 mM), penicillin (100 IU/mL), streptomycin (100 μg/mL), 100 μg/mL Normocin™, and 100 μg/mL of the selective antibiotic Zeocin™. All cell lines were purchased from the American Type Culture Collection (ATCC), except for A2780 and A431 which were from the European Collection of Authenticated Cell Cultures (ECACC), and SJ-G2 cell line which was from Dr Mary Danks, St Jude Children’s Research hospital, Memphis, TN.

#### 4.4.1 *In vitro* growth inhibition assay

Cells in logarithmic growth were transferred to 96-well plates. Cytotoxicity was determined by plating cells in duplicate in 100 μL medium at a density of 2,500–4,000 cells/well in 96 well plates. On day 0, (24 h after plating) when the cells were in logarithmic growth 100 μL medium with or without the test agent was added to each well. After 72 h drug exposure growth inhibitory effects were evaluated using the MTT (3-[4,5-dimethylthiazol-2-yl]-2,5-diphenyltetrazolium bromide) assay with absorbance read at 540 nm. An eight-point dose response curve was produced, using MS Excel software. From these dose-response curves, the GI_50_ value was calculated, representing the drug concentration at which cell growth is 50% inhibited based on the difference between the optical density values on day 0 and those at the end of drug exposure (Bradshaw et al., 2008; Zhang et al., 2009). Each data point was conducted in duplicate and the mean ± S.E.M. calculated from four to five replicates (n = 4 or 5), which were performed on separate occasions and separate cell line passages. Where shown, significant test results were determined using T-test analysis at *p* < 0.01 and *p* < 0.05 with a two tail distribution.

#### 4.4.2 AhR reporter assay

The activity of the AhR signalling pathway was measured using the stably transfected AhR cell line, HT29-Lucia™ (Invivogen, United States). This cell line stably expresses the secreted Lucia luciferase reporter gene under the control of a minimal promoter coupled with the human CYP1A1 gene entire regulatory sequence, containing six DREs. The Lucia luciferase reporter protein is readily measurable in the cell culture supernatant using QUANTI-Luc™. For the determination of AhR activity, 4 × 10^4^ cells in 180 μL was plated into each well of a 96 well micro-titre plate in the absence of antibiotics and allowed to culture for 24 h prior to the addition of test compounds in a volume of 20 μL. At the indicated time, 20 μL of cell supernatant was transferred to a white luminometer plate and 50 µL of QUANTI-Luc was added immediately prior to reading the luminescence using a GloMax Explorer Luminescence Plate Reader. The promoter activity was conducted in duplicate and the values expressed as fold-change relative to DMSO (0.1%) treated cells. The AhR ligand, FICZ (6-formylindolo[3,2-*b*]carbazole) and AhR inhibitor CH223191 were included for study as positive and negative controls, respectively. The reporter cell line was monitored during the analysis (via MTT assay) to ensure that the concentration of compound used did not compromise viability. Results are presented as the mean ± standard error of the mean (SEM) of analysis conducted in duplicate and repeated on three separate occasions.

#### 4.4.3 Gene expression analysis for AhR, CYP1A1, CYP1B1 and SULT1A1

Gene expression was examined in MDA-MB-468 cells following 6 h treatment. Total RNA was extracted using the RNeasy Mini Kit (Qiagen) according to the manufacturer’s instructions. One µg of RNA was reverse transcribed using the QuanitTect Reverse Transcription Kit (Qiagen) according to the manufacturer’s instructions. Rotor-Gene SYBR Green PCR Kit (Qiagen) was used to perform qPCR on a Rotor-Gene 3,000 Thermo-Cycler Instrument using β2-microglobulin as a housekeeping gene (Qiagen). The primer sequences were purchased from Qiagen as follows: AhR (QT02422938), CYP1A1 (QT00012341), CYP1B1 (QT00209496), SULT1A1 (QT01665489), and β2M (QT00088935). HotStar Taq activation (95 °C for 5 min) was followed by 40 cycles of denaturation (95 °C for 5 s), and annealing/extension (60 °C for 10 s). The comparative C_t_ value method was used for data analysis. Results are presented as the mean ± standard error of the mean (SEM) of analysis conducted in duplicate and repeated on two separate occasions. Significant test results were determined using T-test analysis at *p* < 0.01 with a two tail distribution.

## Data Availability

The original contributions presented in the study are included in the article/Supplementary Material, further inquiries can be directed to the corresponding author.
