# Supplementary material for: Next-generation of BBQ analogues that selectively target breast cancer
Source: Front Chem. 2024 Jun 21;12:1396105. doi: 10.3389/fchem.2024.1396105 (PMC11224556; doi:10.3389/fchem.2024.1396105)
Supplement: Supplementary file 1 [file DataSheet1.docx]

Next generation of BBQ analogues that selectively target breast cancer

Jennifer R. Baker,^1†^ Jayne Gilbert,^2†^ Nicholas S O’Brien,^1†^ Cecilia C. Russell,^1^ Adam McCluskey,^1^ and Jennette A. Sakoff ^1,2*^

^1^ Chemistry, School of Environmental & Life Sciences, The University of Newcastle, University Drive, Callaghan NSW 2308, Australia.

^2^ Experimental Therapeutics Group, Department of Medical Oncology, Calvary Mater Newcastle Hospital, Edith Street, Waratah NSW 2298, Australia. E-mail: Jennette.sakoff@newcastle.edu.au.

Supplementary Material

# CYP1 and SULT1A1 functionality

A B

#
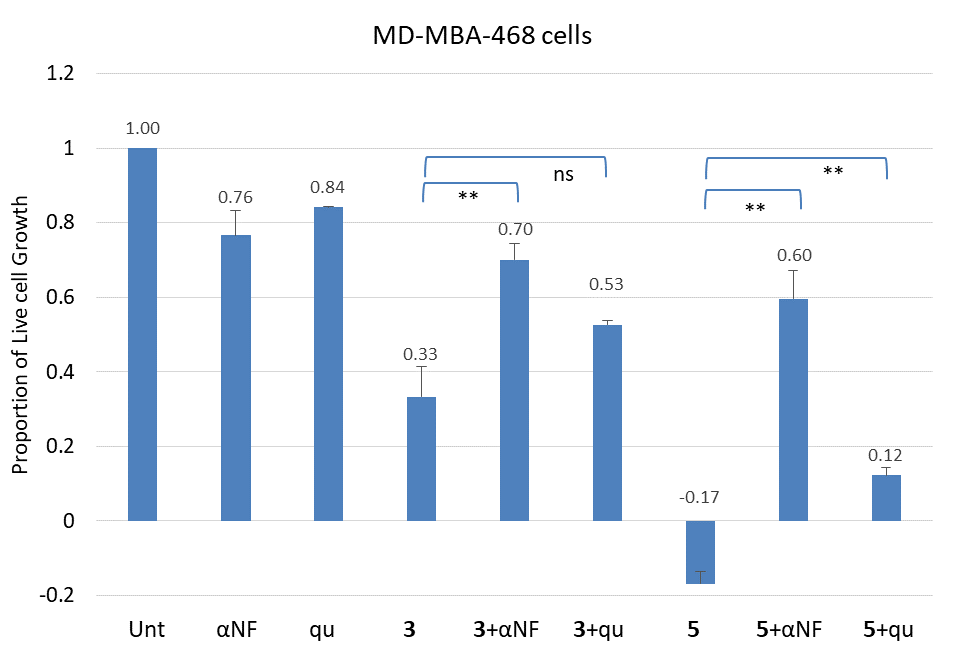

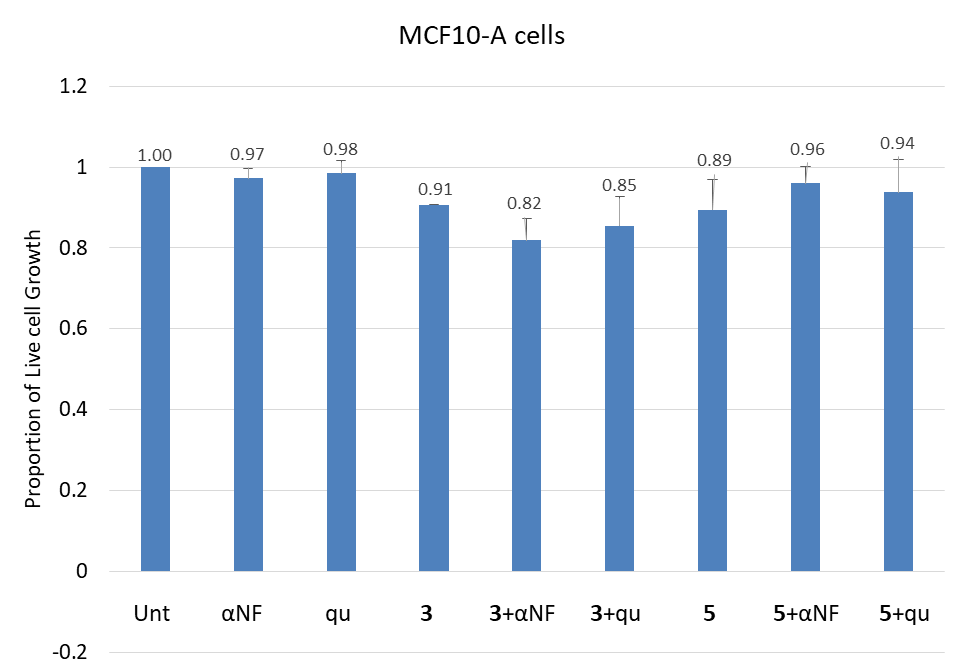


Supp Figure 1. CYP1 and SULT1A1 inhibition ameliorates the effect of **3** and **5**. Growth inhibition (72 h, MTT assay) of **3** and **5** (0.1 µM) in the presence of the CYP1 family inhibitor, α-naphthoflavone (αNF, 10 µM) and SULT1A1 inhibitor, quercetin (qu, 5 µM) in (A) MDA-MB-468 breast cancer cells and (B) MCF10A normal breast cells. Significant differences are at the *P* < 0.01 ** level, using a paired T-test with a two-tailed distribution.

# Computational Methods and Results

AhR modelling (Paula et al., 2019):

| 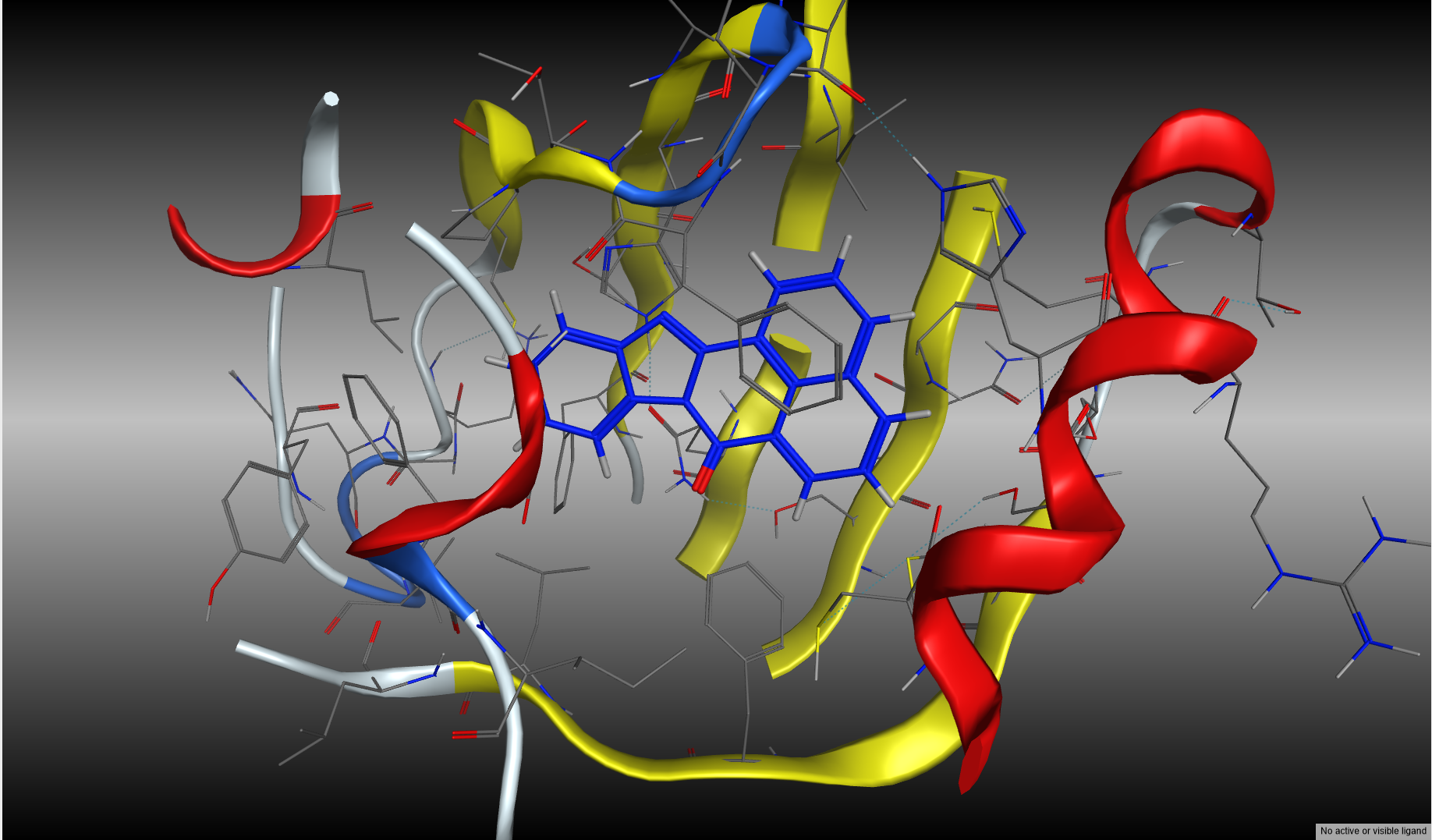 |
| --- |
| 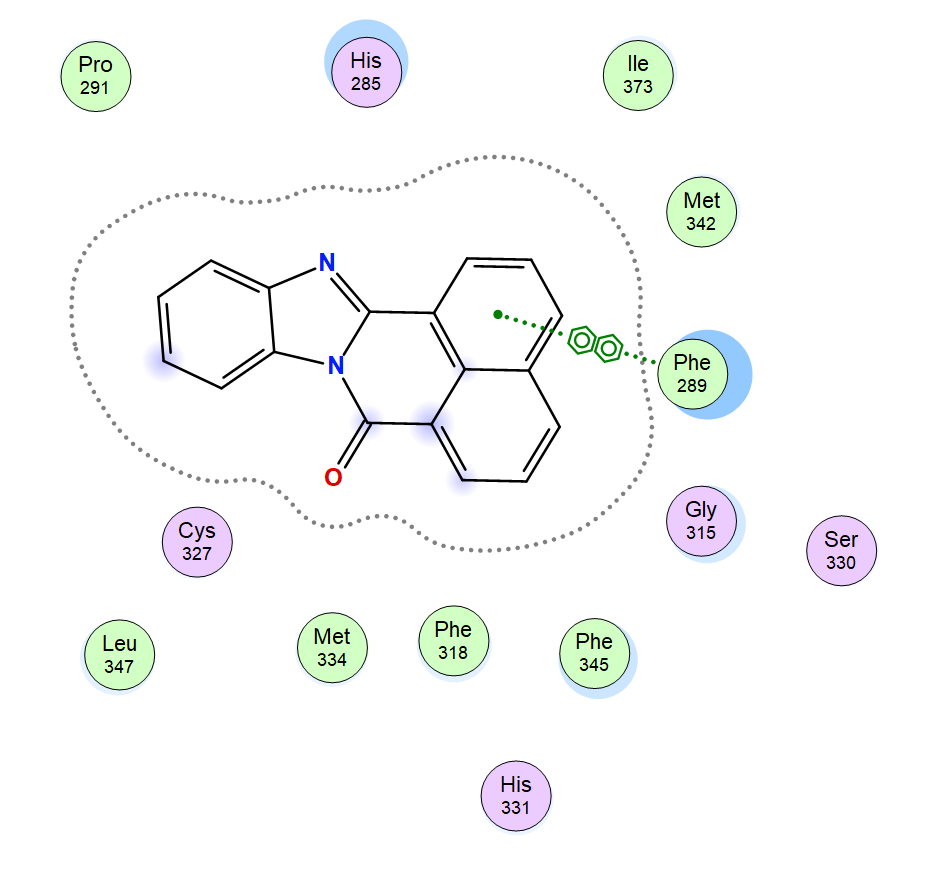 |
| 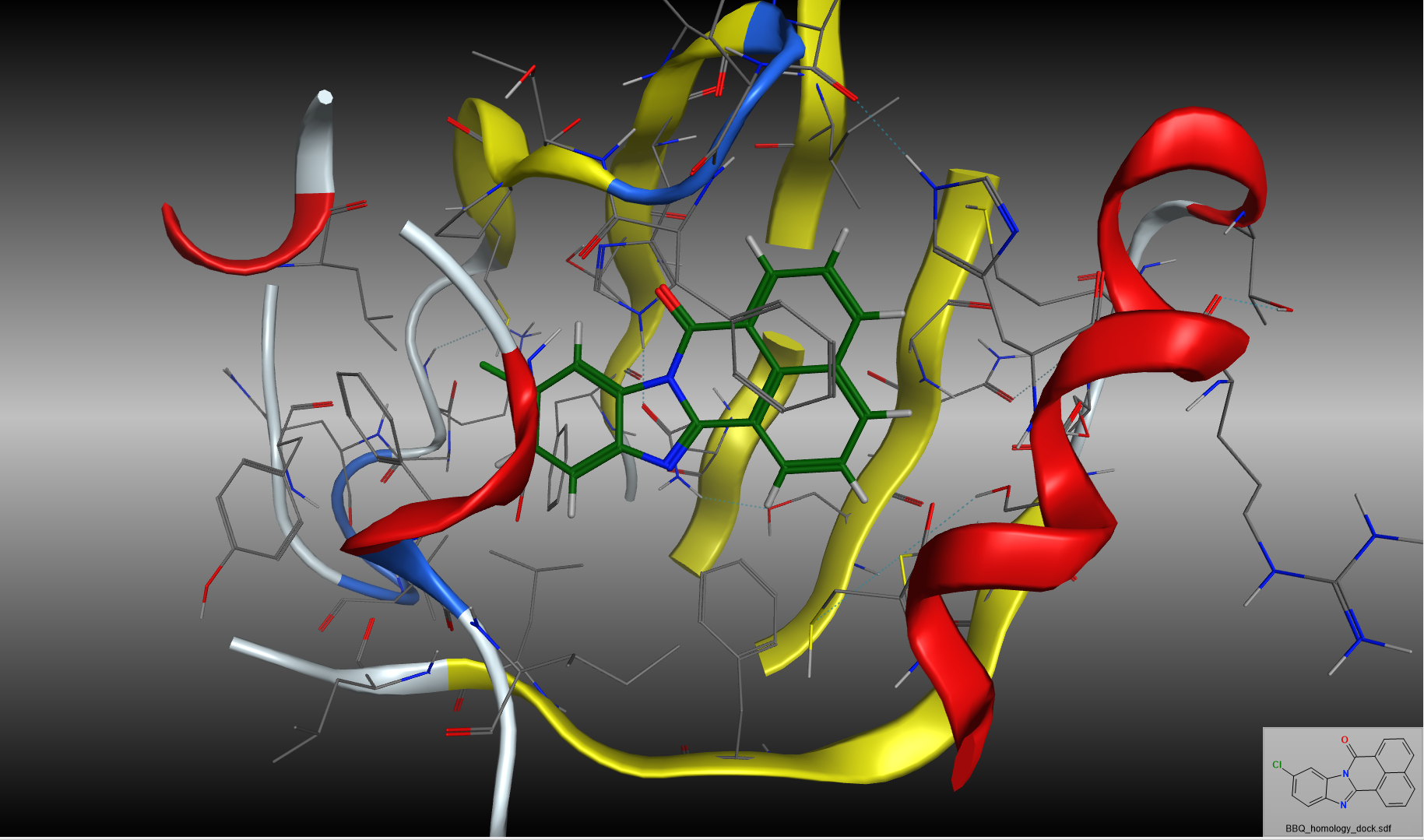 |
| 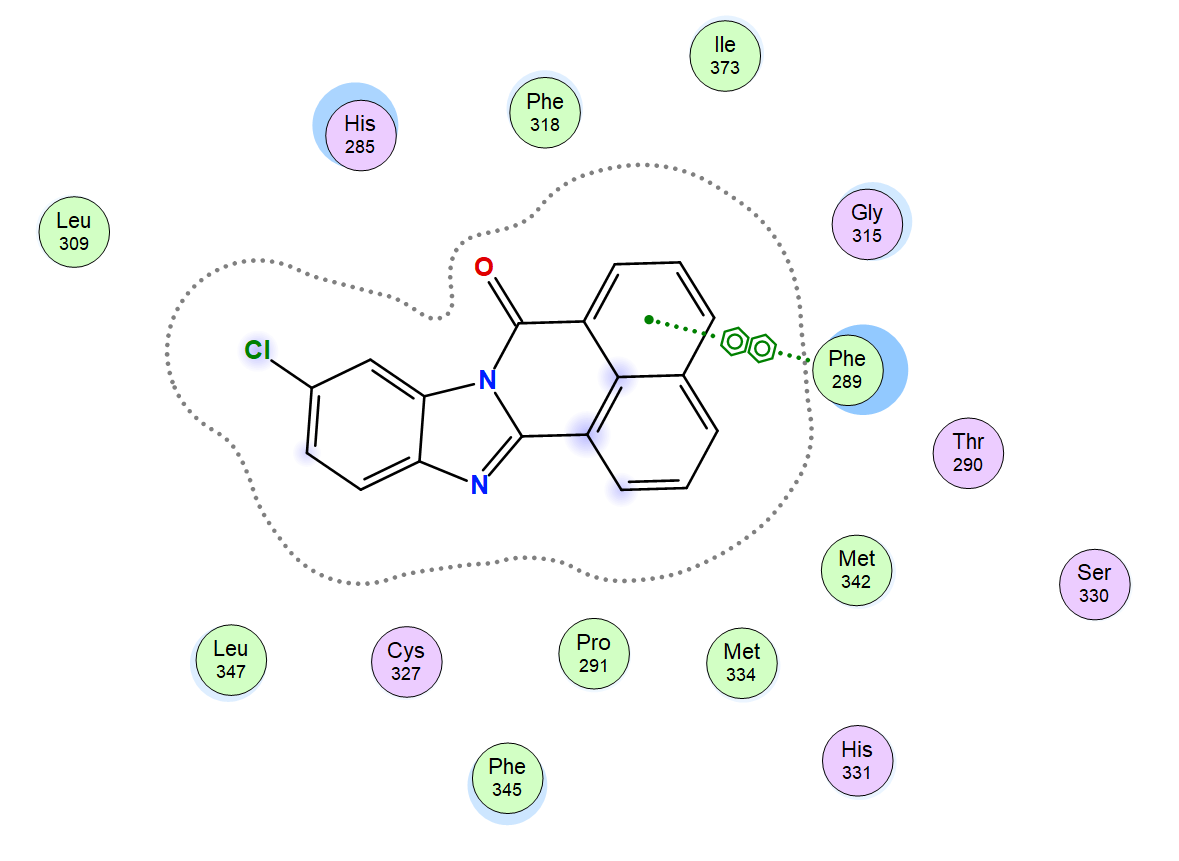 |
| 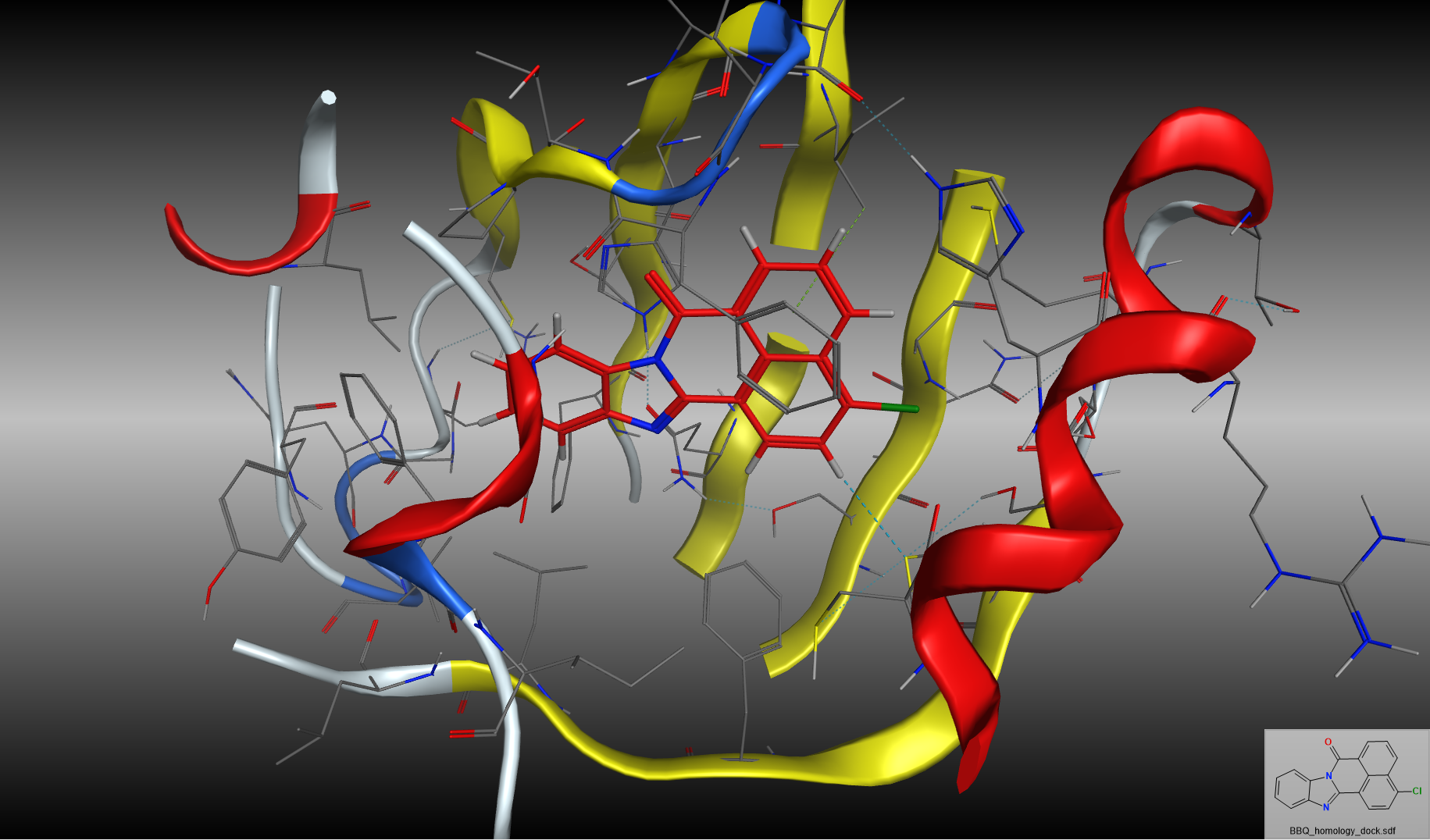 |
| 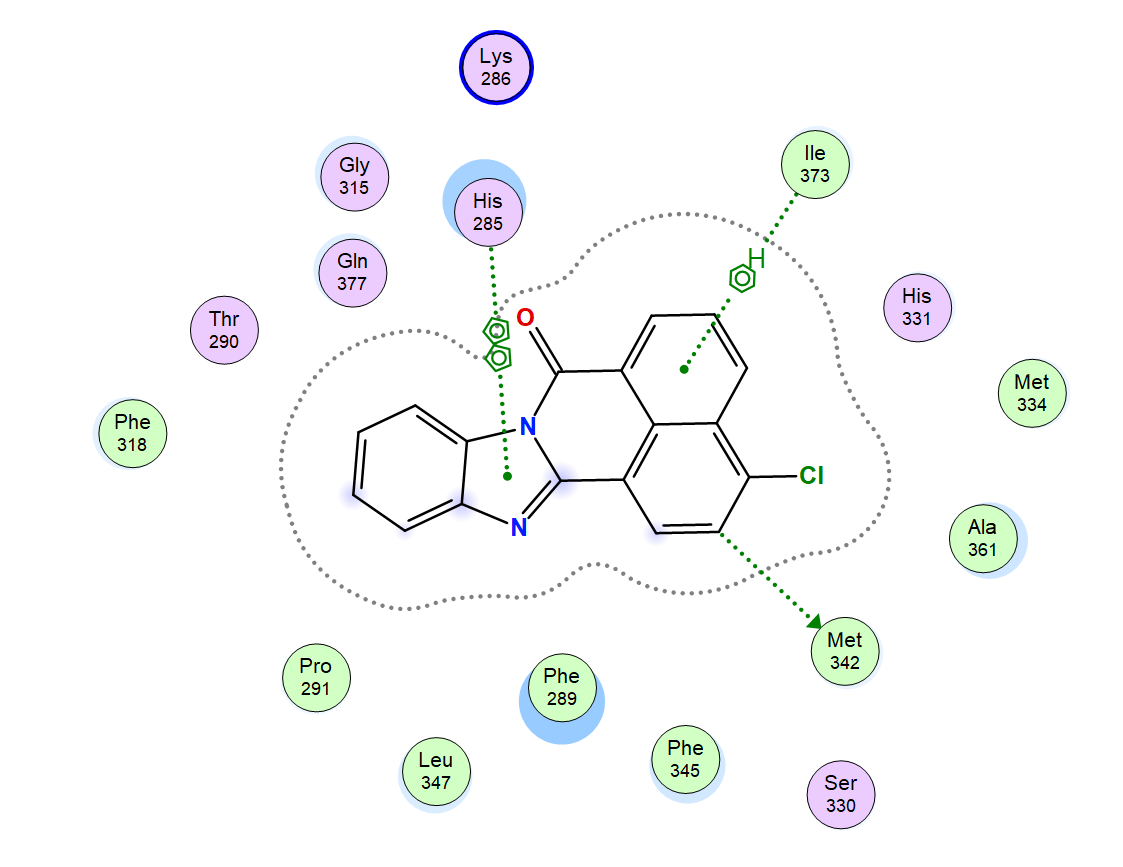 |
| 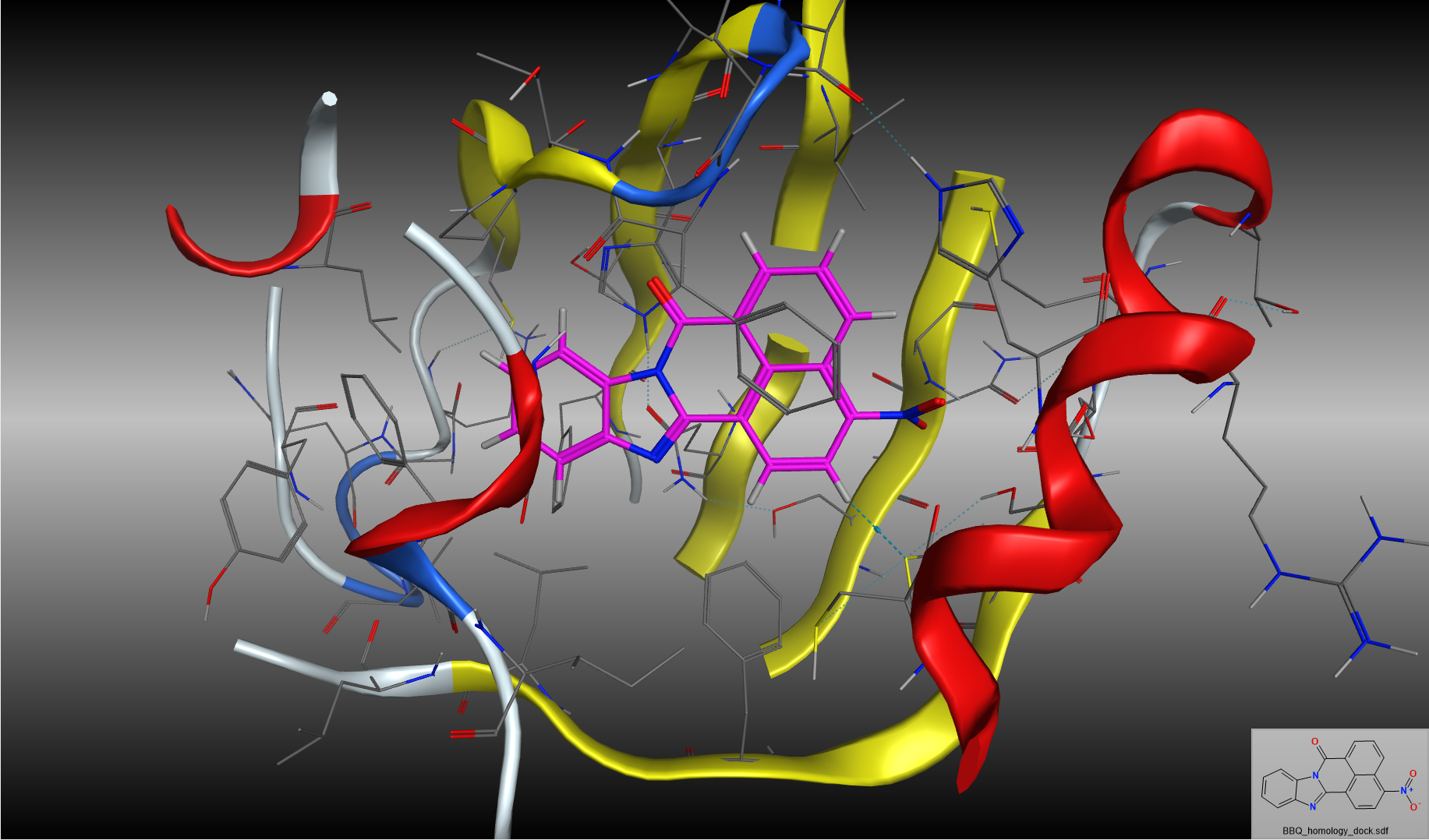 |
| 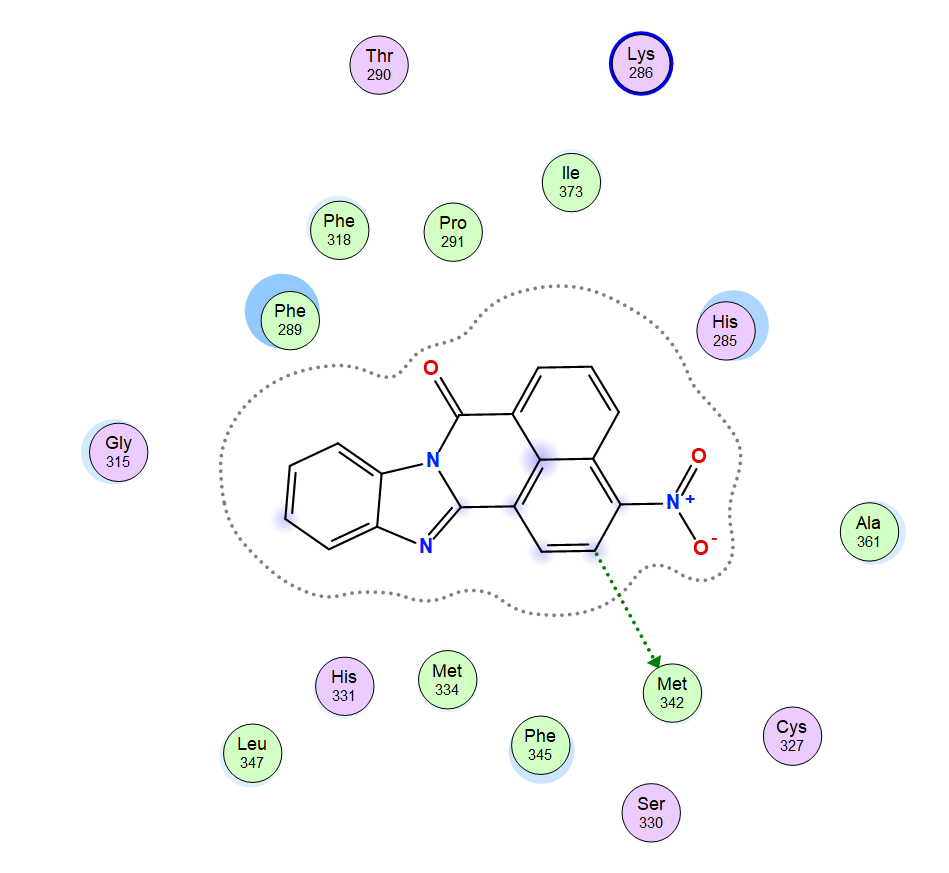 |

**Cyp1A1 modelling**

PDB: 4I8V

Endogenous Ligand: $\alpha$-naphthoflavone

| 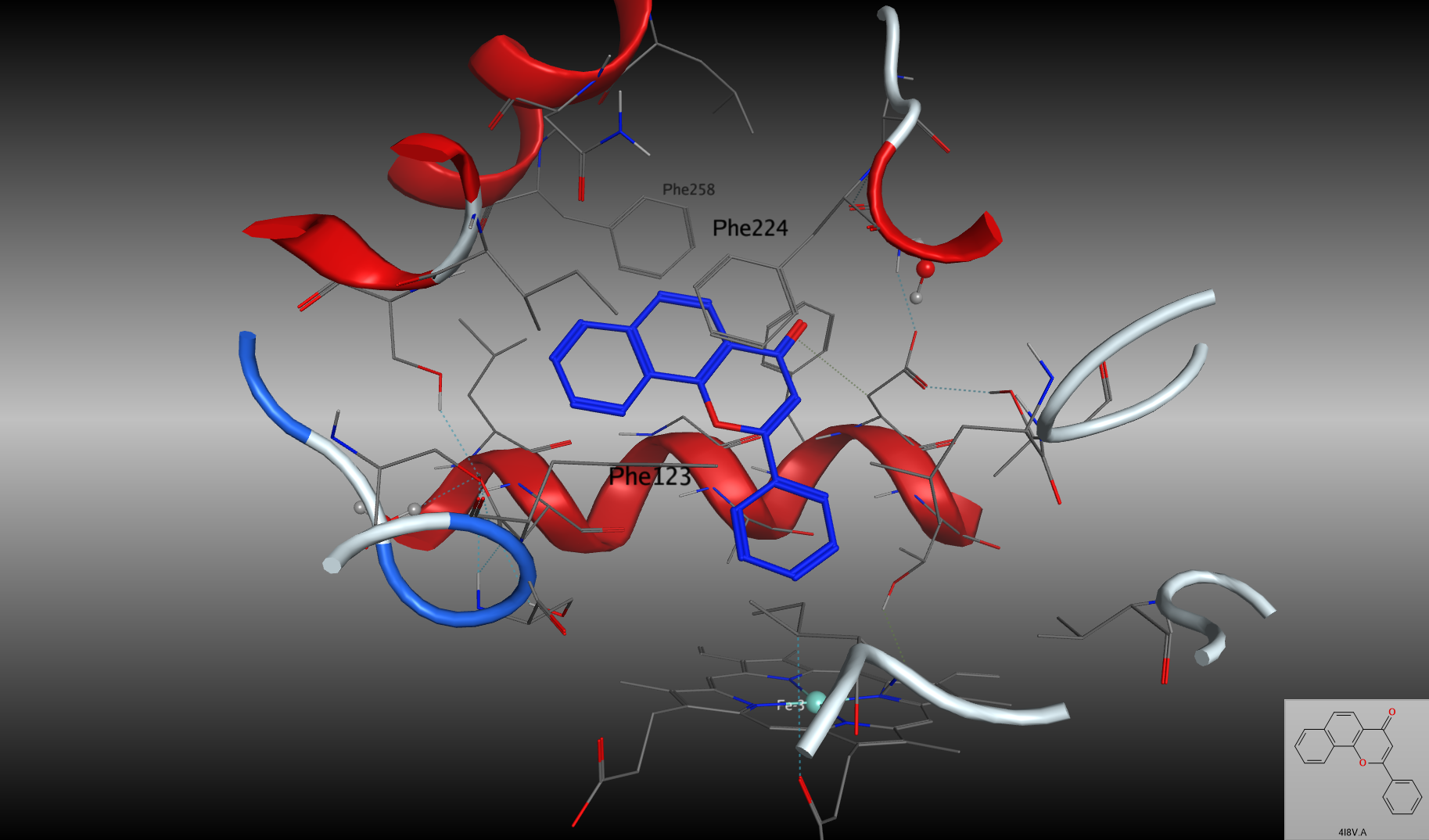 |
| --- |
| 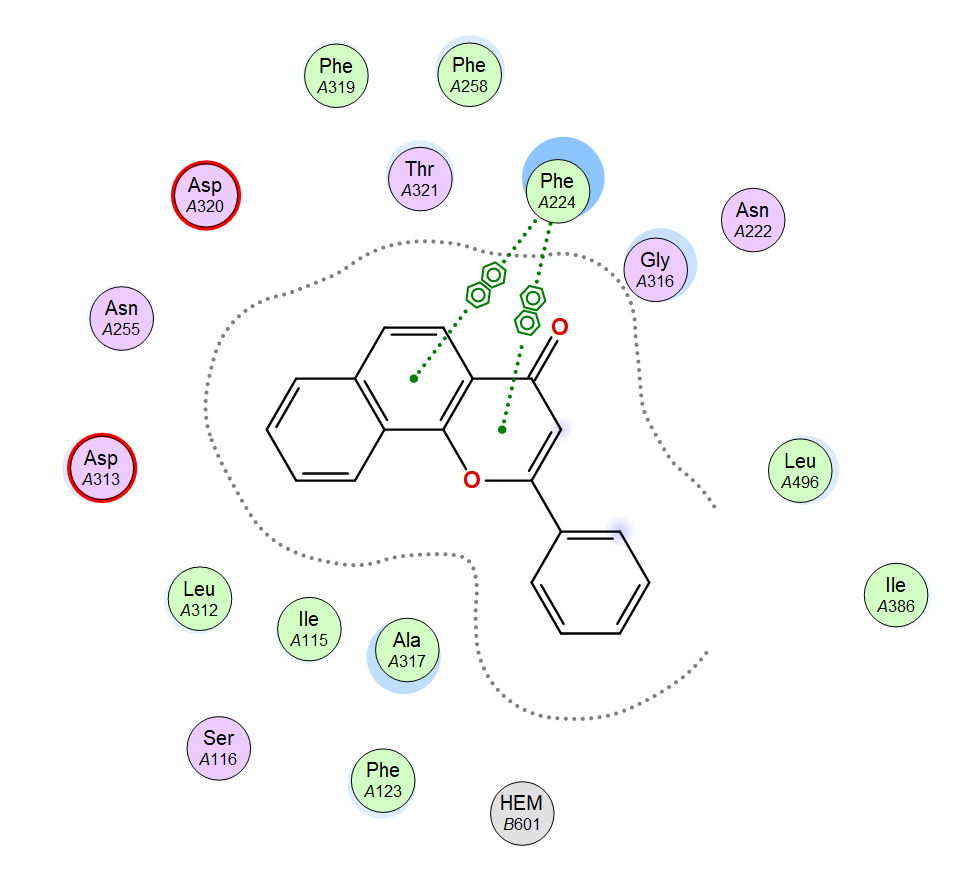 |
| 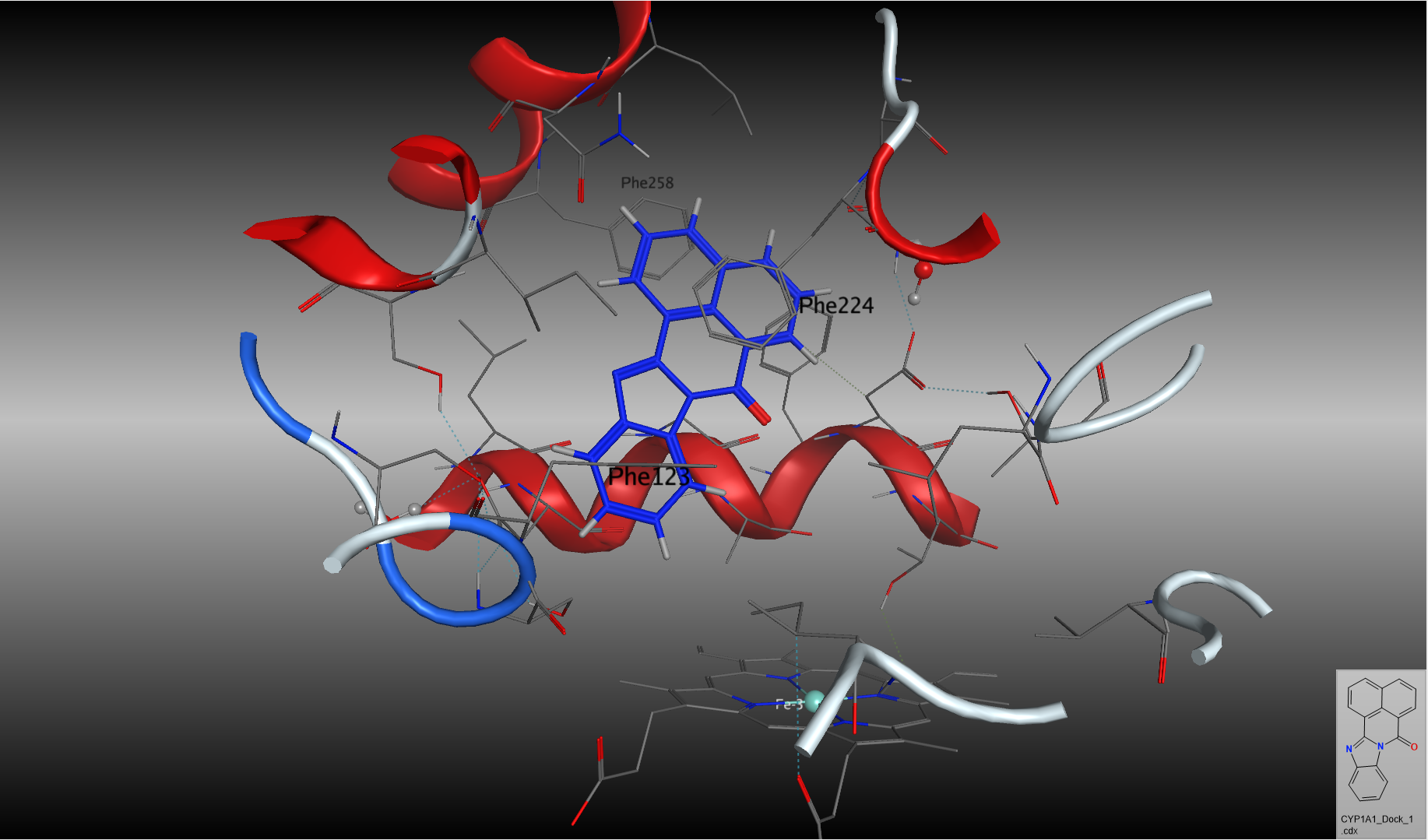 |
| 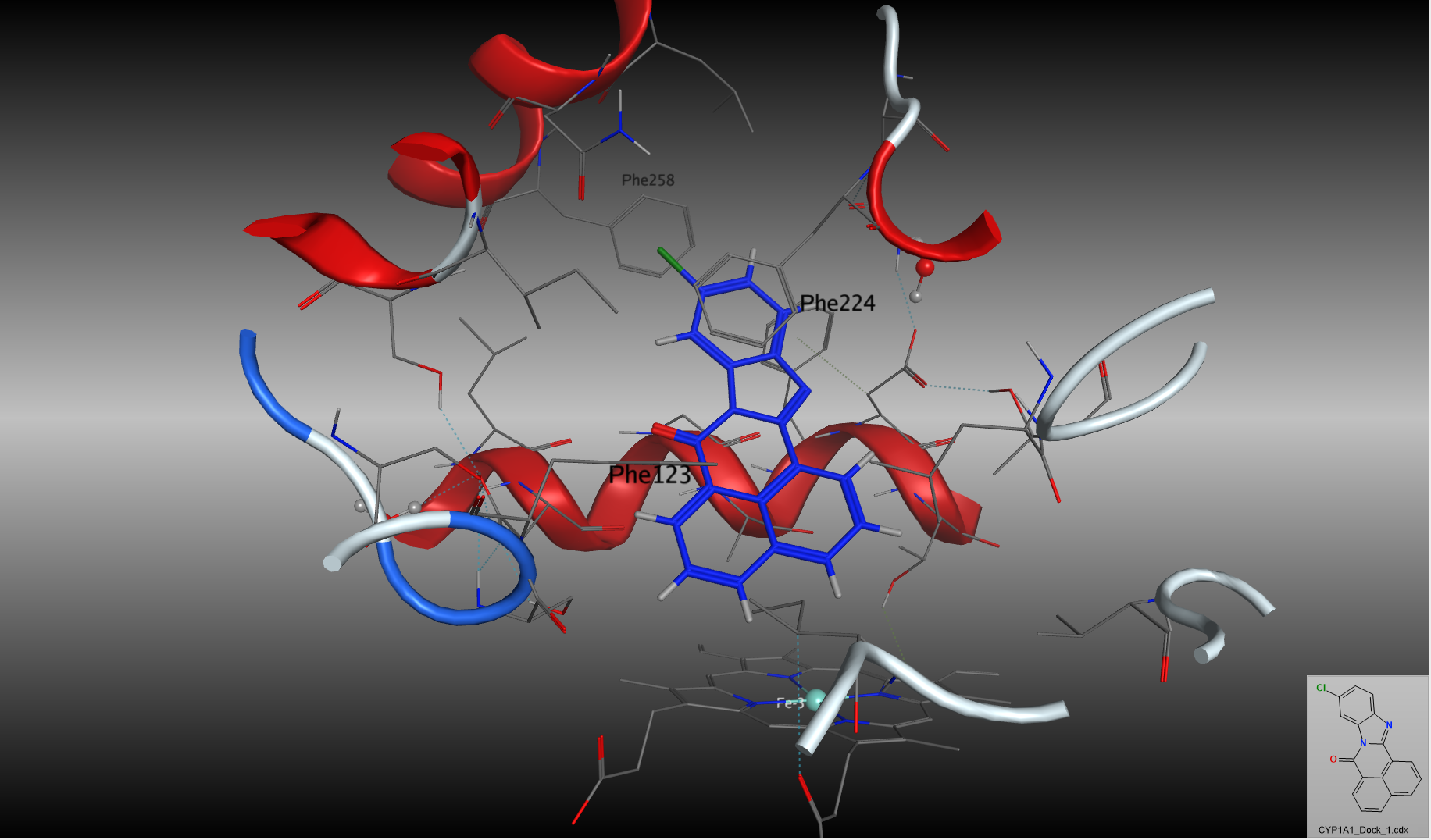 |
| 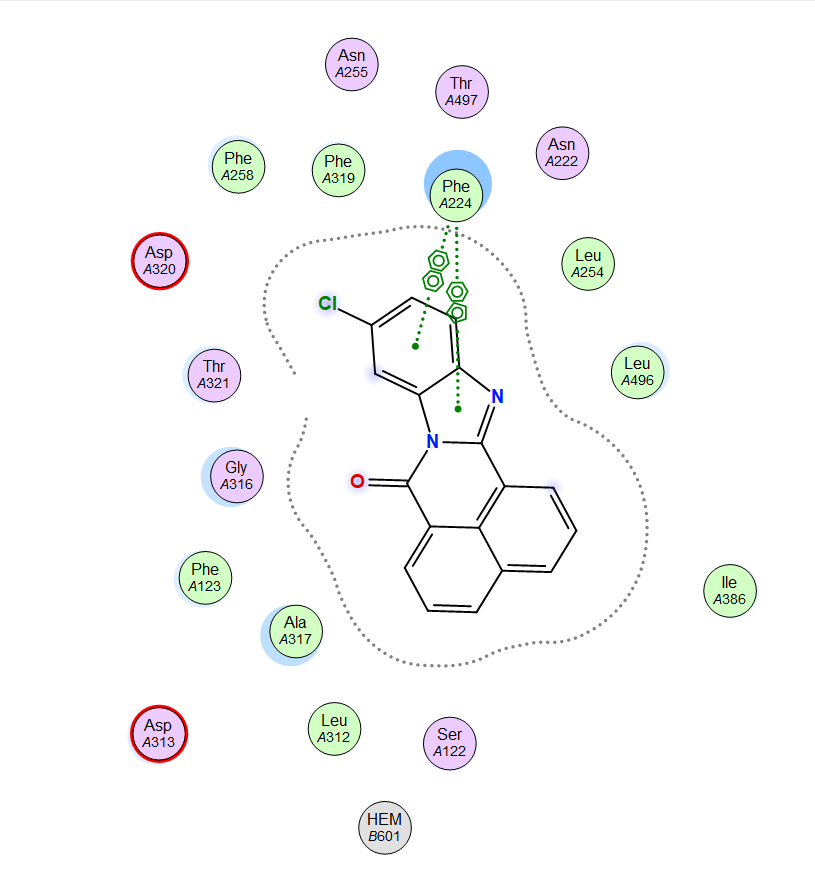  Best Pose Binding Energy: -8.8389 |
| 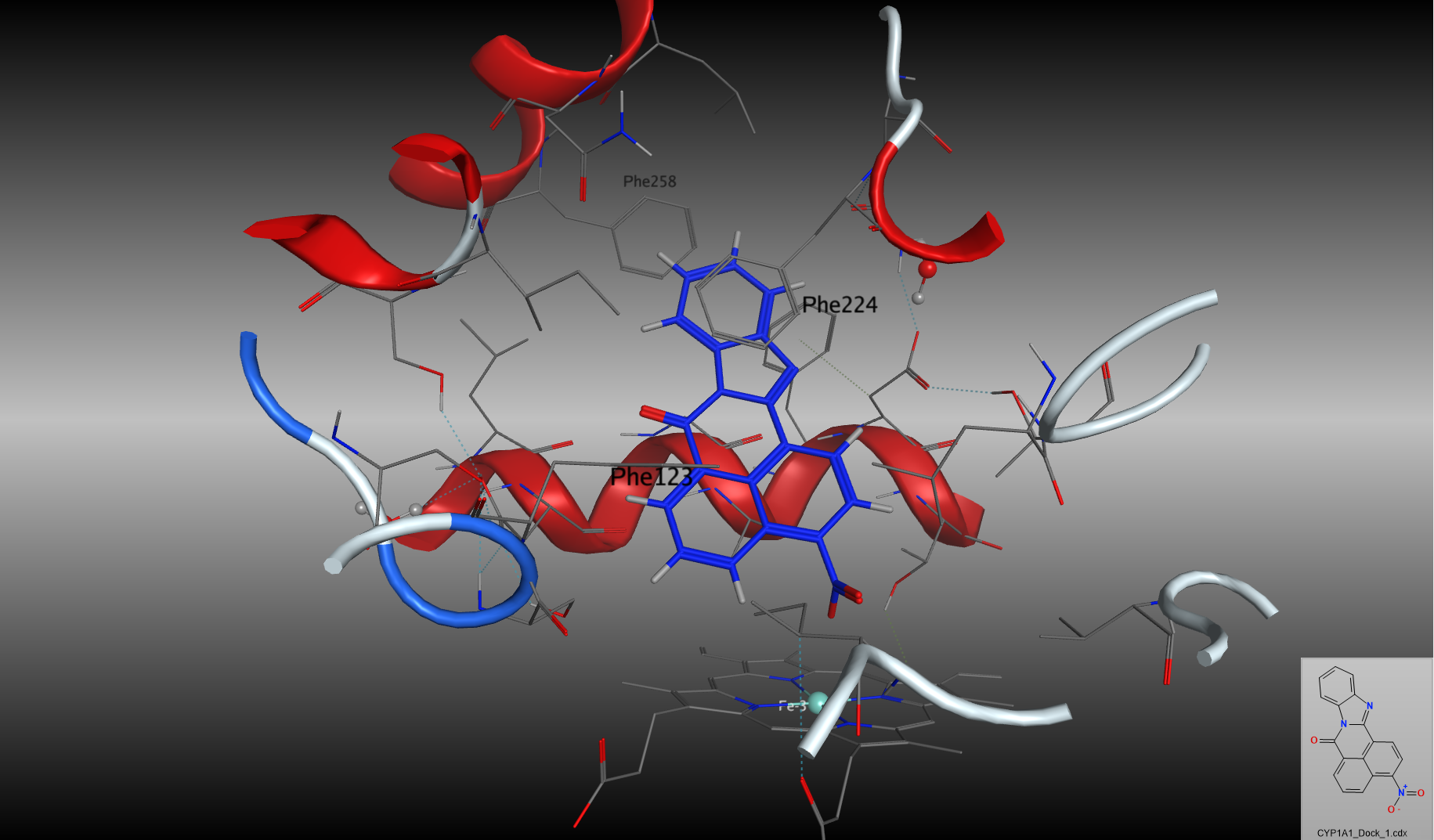 |
| 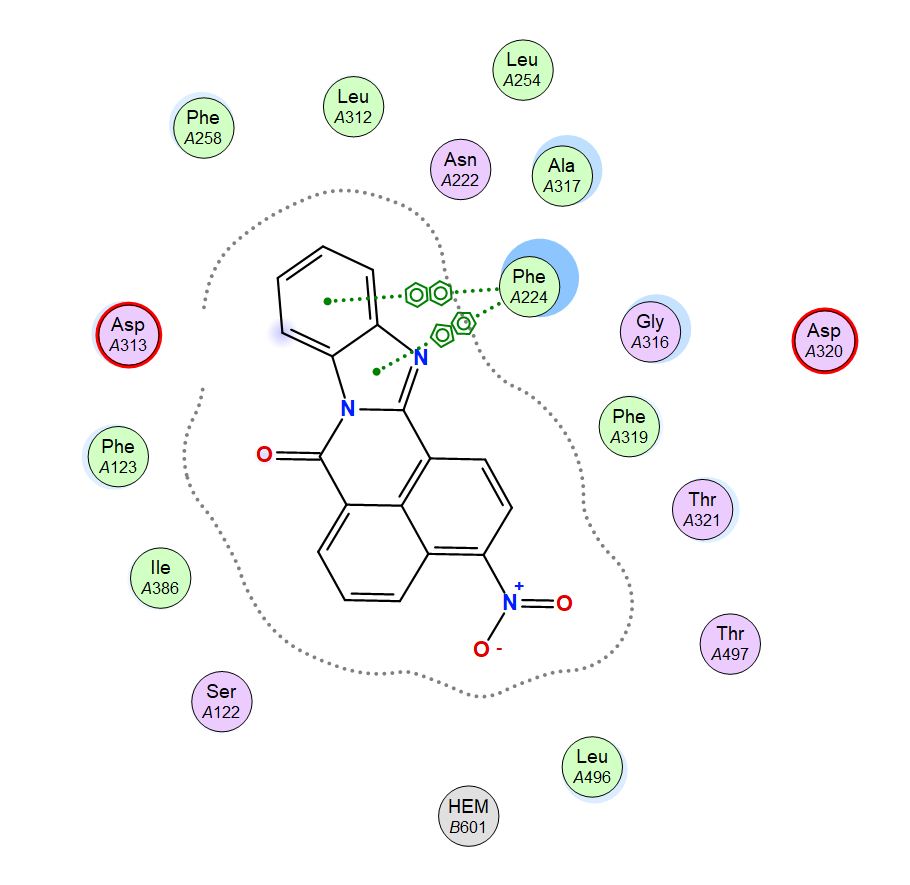  Best Pose Binding Energy: -9.4217 kJ/mol |

**Density Functional Theory (DFT)**

Computed as described in the experimental section. HOMO/LUMO plots and energies for **3**, **5**, **10b**, **11b.** Visualised in Avogadro v.1.2.0

| **Compound** | **HOMO (isovalue = 0.02)** | **LUMO (isovalue = 0.02)** | **LUMO-HOMO (eV)** |
| --- | --- | --- | --- |
| ****  **3** | 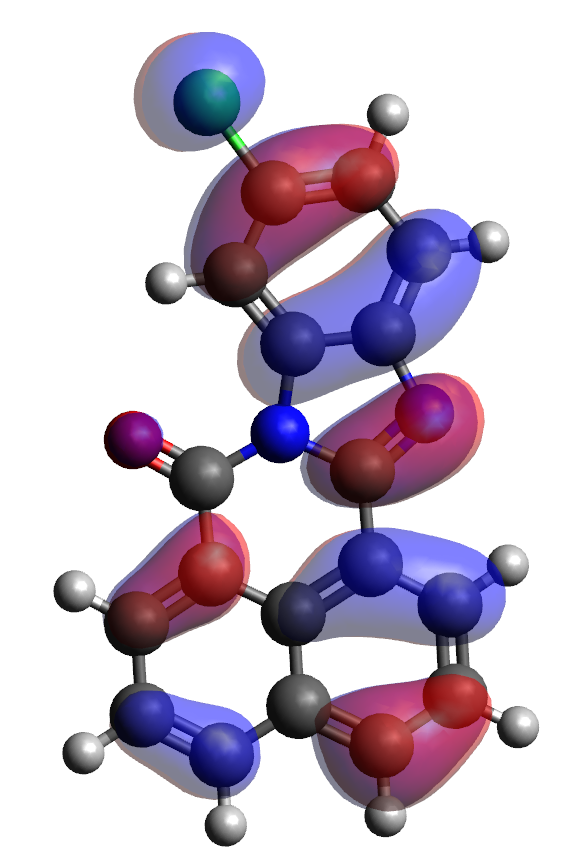 | 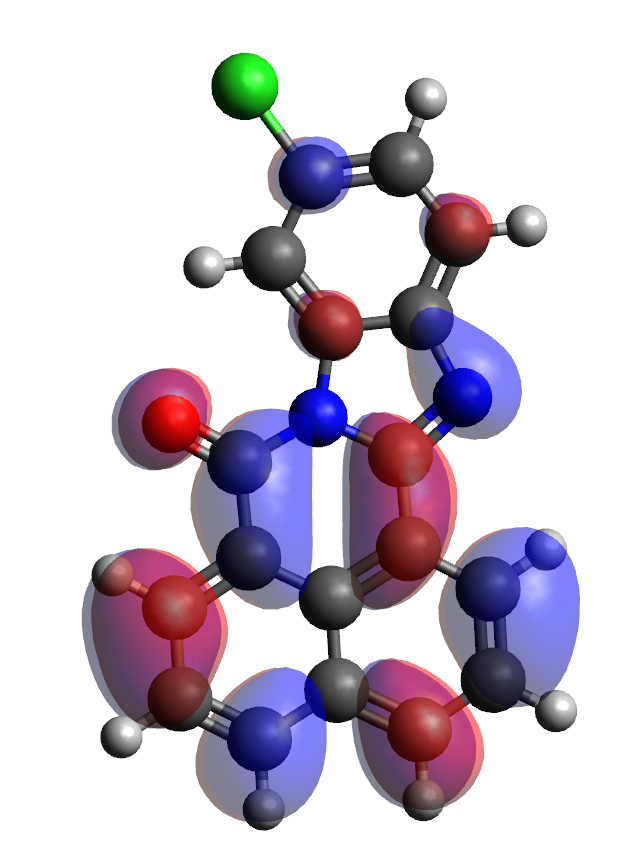 | 1.7150 |
| ****  **5** | 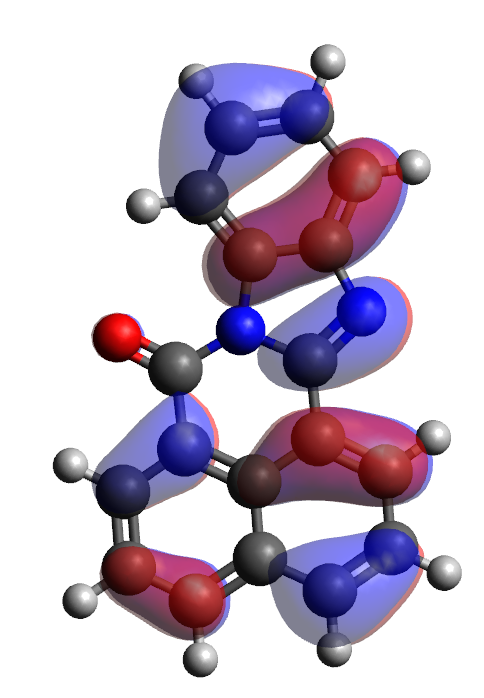 | 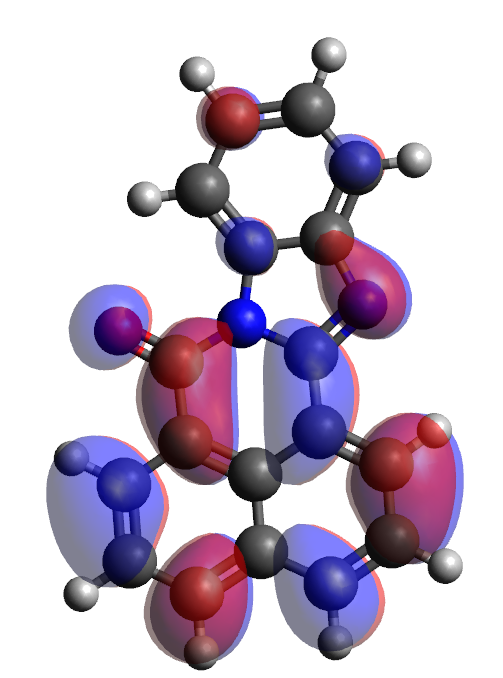 | 1.7811 |
| ****  **10b** | 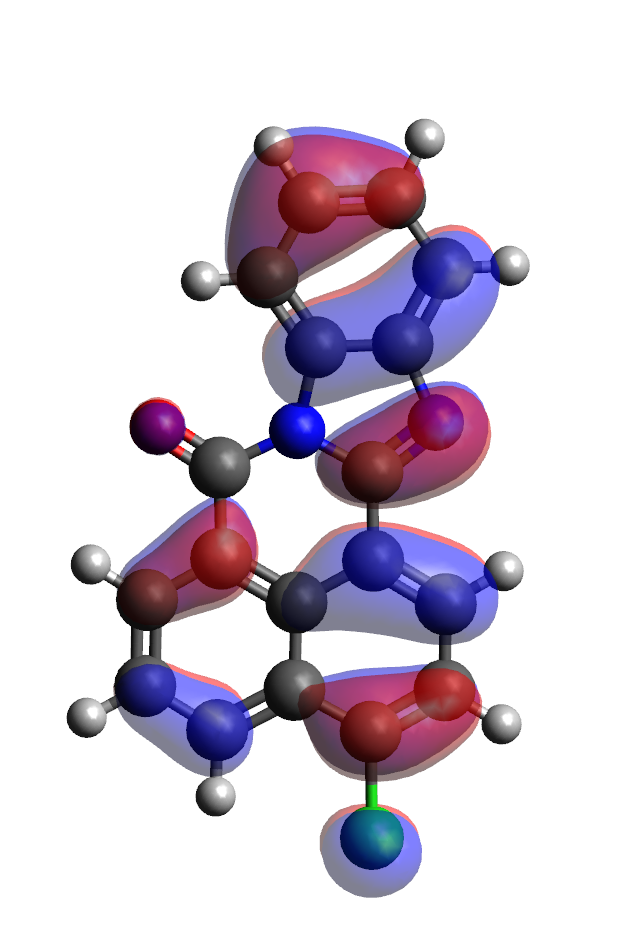 | 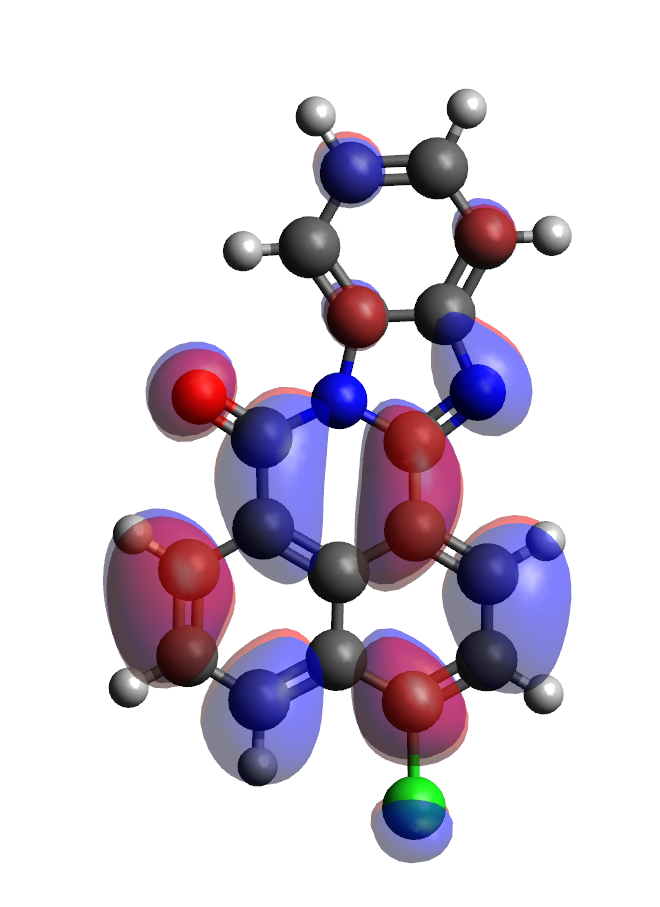 | 1.7976 |
| ****  **11b** | 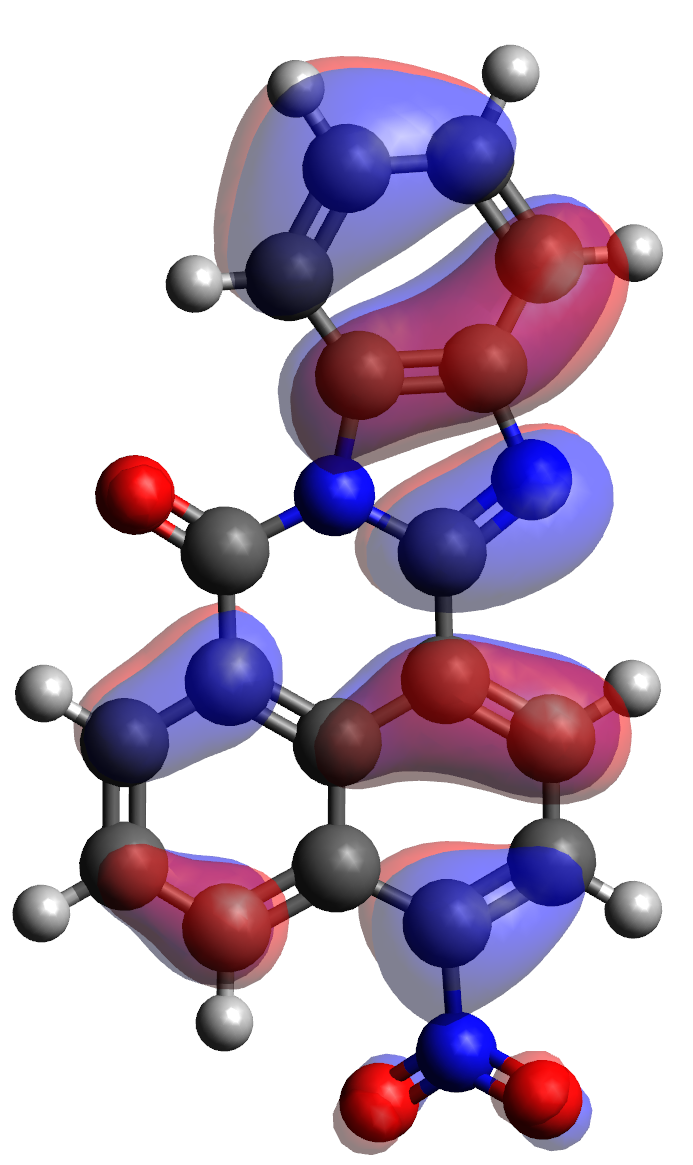 | 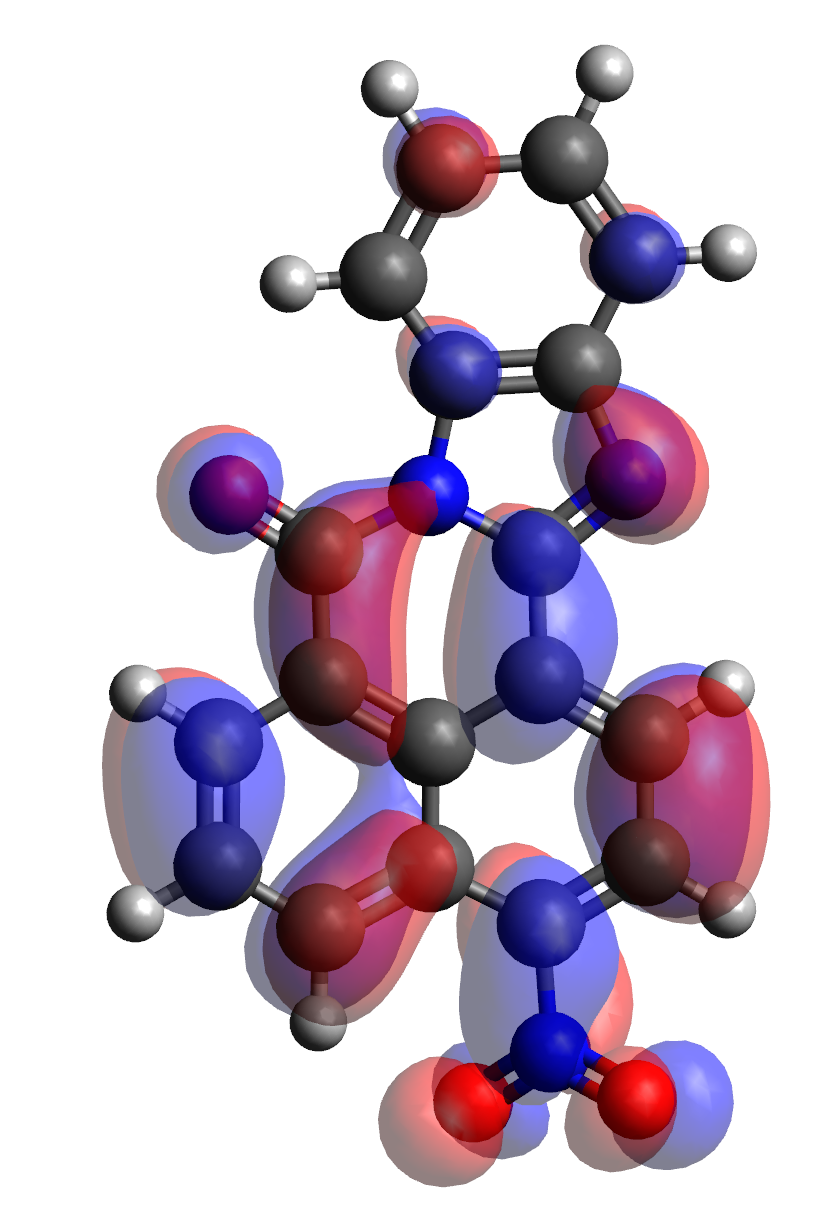 | 0.5646 |

Fukui Indices for **FICZ, 8-OH FICZ**, **3**, **4**, **5**, **10a, 10b**, **11a**, **11b, 13, OH-3, OH-4, OH-5, OH-10b, OH-11b:**

The Fukui function for electrophilic attack, $f^{-}(r)$, is afforded by:

$$f^{-}\left( r \right)=\rho_{N}(r)-\rho_{N-1}(r)$$

Where $\rho_{N}(r)$ is the electronic density of the neutral state, and $\rho_{N-1}(r)$ the electronic density of the molecule with one less electron. Likewise, the Fukui function for nucleophilic attack, $f^{+}(r)$:

$$f^{+}(r)=\rho_{N+1}(r)-\rho_{N}(r)$$

Where $\rho_{N+1}(r)$ is the electronic density after the addition of one electron. Finally, the position most likely to undergo a radical or SET reaction, $f^{0}(r)$, can be calculated by:

$$f^{0}\left( r \right)=\frac{1}{2}[\rho_{N+1}\left( r \right)-\rho_{N-1}\left( r \right)]$$

Calculation of $p_{N-1}(r)$ and $p_{N+1}(r)$ was conducted via the previously described functional/basis set combination using the optimised geometry of the neutral molecule identified in the initial calculations and the associated Hirshfeld charge population densities.

**FICZ**

|  | **Atomic Charges** | | | **Fukui Indices** | | |
| --- | --- | --- | --- | --- | --- | --- |
| **Atom Number** | **Neutral** | **Anion** | **Cation** | $\boldsymbol{F}^{\boldsymbol{+}}$ | $\boldsymbol{F}^{\boldsymbol{-}}$ | $\boldsymbol{F}^{\boldsymbol{0}}$ |
| **1** | -0.25501 | -0.38565 | -0.27517 | 0.13064 | -0.020157 | 0.055243 |
| **2** | 0.194791 | 0.170114 | 0.236072 | 0.02468 | 0.041281 | 0.032979 |
| **3** | -0.01595 | -0.06992 | -0.01117 | 0.05397 | 0.004775 | 0.029373 |
| **4** | 0.000548 | 0.006331 | 0.017668 | -0.00578 | 0.017120 | 0.005668 |
| **5** | -0.00725 | -0.0209 | -0.00383 | 0.01365 | 0.003422 | 0.008538 |
| **6** | 0.094825 | 0.064997 | 0.17034 | 0.02983 | 0.075515 | 0.052672 |
| **7** | -0.17615 | -0.22964 | -0.15834 | 0.05349 | 0.017814 | 0.035652 |
| **8** | -0.05737 | -0.09311 | -0.0356 | 0.03574 | 0.021773 | 0.028757 |
| **9** | -0.0109 | -0.05395 | 0.047205 | 0.04305 | 0.058104 | 0.050578 |
| **10** | -0.01935 | -0.05904 | 0.007185 | 0.03969 | 0.026535 | 0.033112 |
| **11** | -0.01438 | -0.073 | 0.024323 | 0.05862 | 0.038706 | 0.048664 |
| **12** | -0.02433 | -0.0689 | 0.000032 | 0.04456 | 0.024365 | 0.034464 |
| **13** | -0.00733 | -0.05997 | 0.036048 | 0.05264 | 0.043378 | 0.048008 |
| **14** | -0.03773 | -0.05707 | -0.04996 | 0.01934 | -0.012233 | 0.003556 |
| **15** | 0.108013 | 0.113228 | 0.16588 | -0.00522 | 0.057867 | 0.026326 |
| **16** | -0.21487 | -0.29634 | -0.16563 | 0.08147 | 0.049243 | 0.065357 |
| **17** | 0.027504 | 0.025973 | 0.078844 | 0.00153 | 0.051340 | 0.026436 |
| **18** | 0.040256 | 0.038941 | 0.081596 | 0.00131 | 0.041340 | 0.021327 |
| **19** | -0.04347 | -0.04372 | -0.01176 | 0.00025 | 0.031708 | 0.015979 |
| **20** | 0.031645 | 0.009245 | 0.092231 | 0.02240 | 0.060586 | 0.041493 |
| **21** | -0.0763 | -0.13939 | 0.003394 | 0.06309 | 0.079692 | 0.071391 |
| **22** | -0.04351 | -0.06387 | -0.0085 | 0.02036 | 0.035009 | 0.027683 |
| **23** | -0.02877 | -0.05302 | 0.018359 | 0.02425 | 0.047124 | 0.035688 |
| **7H** | 0.177244 | 0.162376 | 0.204194 | 0.01487 | 0.026950 | 0.020909 |
| **8H** | 0.04009 | 0.012209 | 0.061109 | 0.02788 | 0.021019 | 0.024450 |
| **9H** | 0.048602 | 0.023291 | 0.072669 | 0.02531 | 0.024067 | 0.024689 |
| **11H** | 0.043321 | 0.012614 | 0.063888 | 0.03071 | 0.020567 | 0.025637 |
| **12H** | 0.045105 | 0.013747 | 0.064318 | 0.03136 | 0.019213 | 0.025286 |
| **13H** | 0.047557 | 0.020224 | 0.069275 | 0.02733 | 0.021718 | 0.024526 |
| **19H** | 0.044706 | 0.040798 | 0.066429 | 0.00391 | 0.021723 | 0.012816 |
| **22H** | 0.040863 | 0.022824 | 0.065372 | 0.01804 | 0.024509 | 0.021274 |
| **23H** | 0.047545 | 0.030531 | 0.073465 | 0.01701 | 0.025920 | 0.021467 |
|  | **-0.00008** | **-1.00008** | **0.999923** | **1.00000** | **1.00001** | **1.00000** |

**8-OH FICZ**

|  | **Atomic Charges** | | | **Fukui Indices** | | |
| --- | --- | --- | --- | --- | --- | --- |
| **Atom Number** | **Neutral** | **Anion** | **Cation** | $\boldsymbol{F}^{\boldsymbol{+}}$ | $\boldsymbol{F}^{\boldsymbol{-}}$ | $\boldsymbol{F}^{\boldsymbol{0}}$ |
| **1** | -0.2974 | -0.40194 | -0.23741 | 0.10454 | 0.059995 | 0.082267 |
| **2** | 0.163843 | 0.064978 | 0.184384 | 0.09887 | 0.020541 | 0.059703 |
| **3** | -0.04449 | -0.08234 | 0.019599 | 0.03785 | 0.064084 | 0.050968 |
| **4** | 0.067074 | 0.0483 | 0.081736 | 0.01877 | 0.014662 | 0.016718 |
| **5** | -0.02255 | -0.06232 | -0.01224 | 0.03977 | 0.010312 | 0.025040 |
| **6** | -0.02954 | -0.0354 | -0.00799 | 0.00586 | 0.021554 | 0.013705 |
| **7** | -0.03114 | -0.05575 | -0.01362 | 0.02461 | 0.017520 | 0.021065 |
| **8** | -0.05409 | -0.07933 | -0.00548 | 0.02524 | 0.048609 | 0.036925 |
| **9** | -0.03151 | -0.0739 | -0.0053 | 0.04240 | 0.026209 | 0.034302 |
| **10** | -0.05167 | -0.06797 | -0.01787 | 0.01631 | 0.033796 | 0.025051 |
| **11** | 0.059072 | 0.042317 | 0.065005 | 0.01675 | 0.005933 | 0.011344 |
| **12** | -0.10316 | -0.11103 | -0.0232 | 0.00788 | 0.079954 | 0.043915 |
| **13** | -0.03017 | -0.11741 | 0.023556 | 0.08724 | 0.053725 | 0.070485 |
| **14** | 0.040554 | 0.030076 | 0.062582 | 0.01048 | 0.022028 | 0.016253 |
| **15** | -0.01465 | -0.05546 | -0.00285 | 0.04081 | 0.011796 | 0.026301 |
| **16** | -0.02647 | -0.02779 | -0.00438 | 0.00132 | 0.022086 | 0.011705 |
| **17** | -0.06437 | -0.08372 | -0.04491 | 0.01935 | 0.019464 | 0.019405 |
| **18** | 0.082136 | 0.061568 | 0.118772 | 0.02057 | 0.036636 | 0.028602 |
| **19** | -0.22823 | -0.24897 | -0.196 | 0.02075 | 0.032230 | 0.026489 |
| **20** | -0.04193 | -0.08969 | -0.01403 | 0.04776 | 0.027900 | 0.037828 |
| **21** | -0.04432 | -0.0753 | -0.01386 | 0.03098 | 0.030458 | 0.030720 |
| **22** | 0.046237 | 0.028083 | 0.055176 | 0.01815 | 0.008939 | 0.013547 |
| **23** | -0.11642 | -0.13092 | -0.03473 | 0.01450 | 0.081684 | 0.048093 |
| **2H** | 0.036214 | -0.0008 | 0.053216 | 0.03701 | 0.017002 | 0.027007 |
| **7H** | 0.035496 | 0.020811 | 0.049776 | 0.01468 | 0.014280 | 0.014483 |
| **8H** | 0.030747 | 0.010499 | 0.056195 | 0.02025 | 0.025448 | 0.022848 |
| **9H** | 0.035716 | 0.010922 | 0.056847 | 0.02479 | 0.021131 | 0.022963 |
| **10H** | 0.037951 | 0.022926 | 0.057094 | 0.01503 | 0.019143 | 0.017084 |
| **12H** | 0.128581 | 0.117707 | 0.155385 | 0.01087 | 0.026804 | 0.018839 |
| **13H** | 0.042995 | 0.002493 | 0.068621 | 0.04050 | 0.025626 | 0.033064 |
| **17H** | 0.029445 | 0.018635 | 0.042184 | 0.01081 | 0.012739 | 0.011775 |
| **19H** | 0.172361 | 0.161673 | 0.186349 | 0.01069 | 0.013988 | 0.012338 |
| **20H** | 0.043466 | 0.016275 | 0.0649 | 0.02719 | 0.021434 | 0.024313 |
| **21H** | 0.03891 | 0.017713 | 0.058482 | 0.02120 | 0.019572 | 0.020385 |
| **23H** | 0.141512 | 0.125258 | 0.174252 | 0.01625 | 0.032740 | 0.024497 |
|  | **0.000223** | **-0.99980** | **1.000245** | **1.00002** | **1.00002** | **1.00002** |

**3**

|  | **Atomic Charges** | | | **Fukui Indices** | | |
| --- | --- | --- | --- | --- | --- | --- |
| **Atom Number** | **Neutral** | **Anion** | **Cation** | $\boldsymbol{F}^{\boldsymbol{+}}$ | $\boldsymbol{F}^{\boldsymbol{-}}$ | $\boldsymbol{F}^{\boldsymbol{0}}$ |
| **1** | -0.30108 | -0.38139 | -0.27271 | 0.08031 | 0.028370 | 0.054338 |
| **2** | 0.230309 | 0.175105 | 0.237059 | 0.05520 | 0.006750 | 0.030977 |
| **3** | -0.02269 | -0.06615 | -0.01383 | 0.04347 | 0.008858 | 0.026163 |
| **4** | 0.005104 | 0.003977 | 0.014663 | 0.00113 | 0.009559 | 0.005343 |
| **5** | 0.001095 | -0.00717 | 0.009377 | 0.00826 | 0.008282 | 0.008271 |
| **6** | -0.01633 | -0.07119 | 0.039628 | 0.05486 | 0.055959 | 0.055408 |
| **7** | -0.02872 | -0.06827 | -0.00615 | 0.03955 | 0.022572 | 0.031059 |
| **8** | -0.00078 | -0.05945 | 0.036298 | 0.05867 | 0.037080 | 0.047875 |
| **9** | -0.01057 | -0.05257 | 0.011233 | 0.04200 | 0.021803 | 0.031902 |
| **10** | -0.00699 | -0.07159 | 0.016973 | 0.06460 | 0.023960 | 0.044281 |
| **11** | -0.02988 | -0.06904 | -0.00275 | 0.03916 | 0.027129 | 0.033144 |
| **12** | 0.005111 | -0.05949 | 0.030318 | 0.06460 | 0.025207 | 0.044902 |
| **13** | -0.05717 | -0.05708 | -0.0473 | -0.00009 | 0.009867 | 0.004888 |
| **14** | 0.132268 | 0.111278 | 0.18005 | 0.02099 | 0.047782 | 0.034386 |
| **15** | -0.23331 | -0.29359 | -0.16588 | 0.06027 | 0.067435 | 0.063853 |
| **16** | 0.032158 | 0.02657 | 0.094863 | 0.00559 | 0.062705 | 0.034146 |
| **17** | 0.046427 | 0.038818 | 0.089635 | 0.00761 | 0.043208 | 0.025409 |
| **18** | -0.03862 | -0.04397 | -0.00588 | 0.00536 | 0.032735 | 0.019047 |
| **19** | 0.03648 | 0.009339 | 0.10316 | 0.02714 | 0.066680 | 0.046911 |
| **20** | -0.08954 | -0.13886 | 0.021022 | 0.04932 | 0.110559 | 0.079942 |
| **21** | -0.04135 | -0.06303 | -0.00213 | 0.02168 | 0.039222 | 0.030448 |
| **22** | -0.02679 | -0.05236 | 0.024474 | 0.02558 | 0.051262 | 0.038419 |
| **6H** | 0.043134 | 0.013077 | 0.068752 | 0.03006 | 0.025618 | 0.027838 |
| **7H** | 0.04317 | 0.014233 | 0.063398 | 0.02894 | 0.020228 | 0.024583 |
| **8H** | 0.050923 | 0.020853 | 0.067055 | 0.03007 | 0.016132 | 0.023101 |
| **10H** | 0.045378 | 0.012168 | 0.062148 | 0.03321 | 0.016770 | 0.024990 |
| **11H** | 0.043652 | 0.014058 | 0.062795 | 0.02959 | 0.019143 | 0.024369 |
| **12H** | 0.052236 | 0.020738 | 0.066898 | 0.03150 | 0.014662 | 0.023080 |
| **18H** | 0.04772 | 0.040658 | 0.069977 | 0.00706 | 0.022257 | 0.014660 |
| **21H** | 0.040871 | 0.023185 | 0.068985 | 0.01769 | 0.028114 | 0.022900 |
| **22H** | 0.047762 | 0.031129 | 0.077848 | 0.01663 | 0.030086 | 0.023360 |
|  | **-0.00012** | **-1.00000** | **0.99982** | **0.999987** | **0.999994** | **0.99999** |

**4**

|  | **Atomic Charges** | | | **Fukui Indices** | | |
| --- | --- | --- | --- | --- | --- | --- |
| **Atom Number** | **Neutral** | **Anion** | **Cation** | $\boldsymbol{F}^{\boldsymbol{+}}$ | $\boldsymbol{F}^{\boldsymbol{-}}$ | $\boldsymbol{F}^{\boldsymbol{0}}$ |
| **1** | -0.30313 | -0.38379 | -0.27212 | 0.08066 | 0.031008 | 0.055834 |
| **2** | 0.229907 | 0.174843 | 0.237354 | 0.05506 | 0.007447 | 0.031255 |
| **3** | -0.02271 | -0.06555 | -0.01191 | 0.04285 | 0.010803 | 0.026824 |
| **4** | 0.005132 | 0.003869 | 0.015074 | 0.00126 | 0.009942 | 0.005603 |
| **5** | 0.001073 | -0.00718 | 0.0097 | 0.00825 | 0.008627 | 0.008438 |
| **6** | -0.01571 | -0.07093 | 0.044089 | 0.05523 | 0.059794 | 0.057511 |
| **7** | -0.02851 | -0.06784 | -0.00399 | 0.03933 | 0.024513 | 0.031921 |
| **8** | 0.000157 | -0.05901 | 0.0395 | 0.05917 | 0.039343 | 0.049255 |
| **9** | -0.01068 | -0.0523 | 0.016793 | 0.04161 | 0.027476 | 0.034545 |
| **10** | 0.134225 | 0.111676 | 0.179119 | 0.02255 | 0.044894 | 0.033722 |
| **11** | -0.23368 | -0.29337 | -0.16172 | 0.05969 | 0.071957 | 0.065824 |
| **12** | 0.038243 | 0.031651 | 0.09498 | 0.00659 | 0.056737 | 0.031664 |
| **13** | -0.03682 | -0.05988 | 0.005262 | 0.02306 | 0.042080 | 0.032572 |
| **14** | 0.03592 | 0.019307 | 0.066103 | 0.01661 | 0.030183 | 0.023398 |
| **15** | -0.09193 | -0.13658 | -0.01456 | 0.04465 | 0.077368 | 0.061011 |
| **16** | -0.0408 | -0.07472 | 0.045086 | 0.03393 | 0.085881 | 0.059905 |
| **17** | -0.02852 | -0.03661 | 0.000295 | 0.00809 | 0.028812 | 0.018453 |
| **18** | 0.040125 | 0.033389 | 0.090389 | 0.00674 | 0.050264 | 0.028500 |
| **19** | -0.05723 | -0.05712 | -0.04773 | -0.00011 | 0.009504 | 0.004695 |
| **20** | -0.00687 | -0.07121 | 0.017999 | 0.06434 | 0.024866 | 0.044602 |
| **21** | -0.02997 | -0.06895 | -0.00089 | 0.03898 | 0.029083 | 0.034030 |
| **22** | 0.005133 | -0.05953 | 0.031689 | 0.06466 | 0.026556 | 0.045609 |
| **6H** | 0.043334 | 0.013152 | 0.070248 | 0.03018 | 0.026914 | 0.028548 |
| **7H** | 0.043406 | 0.014527 | 0.064748 | 0.02888 | 0.021342 | 0.025111 |
| **8H** | 0.05138 | 0.021267 | 0.068698 | 0.03011 | 0.017318 | 0.023716 |
| **13H** | 0.050036 | 0.034756 | 0.076573 | 0.01528 | 0.026537 | 0.020909 |
| **16H** | 0.041225 | 0.021196 | 0.076523 | 0.02003 | 0.035298 | 0.027664 |
| **17H** | 0.046026 | 0.03789 | 0.068389 | 0.00814 | 0.022363 | 0.015250 |
| **20H** | 0.04546 | 0.012352 | 0.062804 | 0.03311 | 0.017344 | 0.025226 |
| **21H** | 0.043607 | 0.014068 | 0.06372 | 0.02954 | 0.020113 | 0.024826 |
| **22H** | 0.052051 | 0.020522 | 0.067686 | 0.03153 | 0.015635 | 0.023582 |
|  | **0.000096** | **-1.00009** | **0.999906** | **0.999997** | **1.000002** | **1.00000** |

**5**

|  | **Atomic Charges** | | | **Fukui Indices** | | |
| --- | --- | --- | --- | --- | --- | --- |
| **Atom Number** | **Neutral** | **Anion** | **Cation** | $\boldsymbol{F}^{\boldsymbol{+}}$ | $\boldsymbol{F}^{\boldsymbol{-}}$ | $\boldsymbol{F}^{\boldsymbol{0}}$ |
| **1** | -0.30453 | -0.38493 | -0.27372 | 0.08040 | 0.030810 | 0.055605 |
| **2** | 0.229268 | 0.174574 | 0.236521 | 0.05469 | 0.007253 | 0.030974 |
| **3** | -0.02229 | -0.06755 | -0.01204 | 0.04526 | 0.010249 | 0.027754 |
| **4** | 0.004619 | 0.003209 | 0.014513 | 0.00141 | 0.009894 | 0.005652 |
| **5** | 0.000729 | -0.00767 | 0.009371 | 0.00840 | 0.008642 | 0.008520 |
| **6** | -0.01799 | -0.07308 | 0.041588 | 0.05509 | 0.059578 | 0.057332 |
| **7** | -0.02932 | -0.06982 | -0.0054 | 0.04050 | 0.023915 | 0.032208 |
| **8** | -0.00197 | -0.06056 | 0.037728 | 0.05859 | 0.039699 | 0.049143 |
| **9** | -0.00995 | -0.05272 | 0.014807 | 0.04277 | 0.024754 | 0.033762 |
| **10** | -0.00849 | -0.07407 | 0.016875 | 0.06558 | 0.025361 | 0.045472 |
| **11** | -0.0308 | -0.07101 | -0.00189 | 0.04021 | 0.028911 | 0.034561 |
| **12** | 0.003478 | -0.06145 | 0.030594 | 0.06493 | 0.027116 | 0.046021 |
| **13** | -0.0582 | -0.05821 | -0.04777 | 0.00001 | 0.010436 | 0.005224 |
| **14** | 0.13063 | 0.11058 | 0.179618 | 0.02005 | 0.048988 | 0.034519 |
| **15** | -0.23463 | -0.29538 | -0.1602 | 0.06075 | 0.074427 | 0.067590 |
| **16** | 0.032507 | 0.026096 | 0.092859 | 0.00641 | 0.060352 | 0.033382 |
| **17** | 0.041127 | 0.033384 | 0.085739 | 0.00774 | 0.044612 | 0.026178 |
| **18** | -0.0369 | -0.04512 | -0.0007 | 0.00822 | 0.036202 | 0.022213 |
| **19** | -0.03885 | -0.07475 | 0.049279 | 0.03589 | 0.088132 | 0.062013 |
| **20** | -0.03999 | -0.06445 | 0.000103 | 0.02445 | 0.040097 | 0.032275 |
| **21** | -0.03503 | -0.06079 | 0.02103 | 0.02576 | 0.056056 | 0.040907 |
| **6H** | 0.042238 | 0.011917 | 0.069287 | 0.03032 | 0.027049 | 0.028685 |
| **7H** | 0.042459 | 0.013074 | 0.063791 | 0.02939 | 0.021332 | 0.025359 |
| **8H** | 0.050373 | 0.020133 | 0.067631 | 0.03024 | 0.017258 | 0.023749 |
| **10H** | 0.04454 | 0.010806 | 0.062147 | 0.03373 | 0.017607 | 0.025671 |
| **11H** | 0.042762 | 0.012631 | 0.062999 | 0.03013 | 0.020237 | 0.025184 |
| **12H** | 0.05123 | 0.019335 | 0.067034 | 0.03190 | 0.015804 | 0.023850 |
| **18H** | 0.041403 | 0.032941 | 0.065996 | 0.00846 | 0.024593 | 0.016528 |
| **19H** | 0.034579 | 0.012679 | 0.073251 | 0.02190 | 0.038672 | 0.030286 |
| **20H** | 0.03416 | 0.014498 | 0.064753 | 0.01966 | 0.030593 | 0.025128 |
| **21H** | 0.042719 | 0.025571 | 0.07409 | 0.01715 | 0.031371 | 0.024260 |
|  | **-0.00011** | **-1.0001** | **0.999893** | **0.999998** | **1.00000** | **0.999999** |

**10a**

|  | **Atomic Charges** | | | **Fukui Indices** | | |
| --- | --- | --- | --- | --- | --- | --- |
| **Atom Number** | **Neutral** | **Anion** | **Cation** | $\boldsymbol{F}^{\boldsymbol{+}}$ | $\boldsymbol{F}^{\boldsymbol{-}}$ | $\boldsymbol{F}^{\boldsymbol{0}}$ |
| **1** | -0.25587 | -0.38189 | -0.27375 | 0.12601 | -0.017878 | 0.054068 |
| **2** | 0.19446 | 0.176601 | 0.236132 | 0.01786 | 0.041672 | 0.029765 |
| **3** | -0.01818 | -0.07052 | -0.01507 | 0.05234 | 0.003116 | 0.027728 |
| **4** | 0.00224 | 0.006887 | 0.018739 | -0.00465 | 0.016499 | 0.005926 |
| **5** | -0.0011 | -0.0106 | 0.003776 | 0.00950 | 0.004878 | 0.007189 |
| **6** | -0.0267 | -0.07135 | 0.03791 | 0.04465 | 0.064612 | 0.054629 |
| **7** | -0.02098 | -0.06594 | -0.00245 | 0.04496 | 0.018530 | 0.031744 |
| **8** | -0.00979 | -0.05525 | 0.038044 | 0.04546 | 0.047836 | 0.046648 |
| **9** | -0.00329 | -0.05052 | 0.013394 | 0.04723 | 0.016683 | 0.031954 |
| **10** | 0.048175 | 0.006066 | 0.077298 | 0.04211 | 0.029123 | 0.035616 |
| **11** | -0.03857 | -0.12849 | -0.00281 | 0.08992 | 0.035767 | 0.062844 |
| **12** | -0.03085 | -0.07174 | -0.00929 | 0.04090 | 0.021560 | 0.031228 |
| **13** | -0.00619 | -0.0553 | 0.032979 | 0.04911 | 0.039167 | 0.044140 |
| **14** | -0.03775 | -0.05769 | -0.04726 | 0.01994 | -0.009507 | 0.005216 |
| **15** | 0.107181 | 0.113866 | 0.181235 | -0.00669 | 0.074054 | 0.033685 |
| **16** | -0.20894 | -0.29074 | -0.1613 | 0.08180 | 0.047632 | 0.064717 |
| **17** | 0.029145 | 0.026809 | 0.096358 | 0.00234 | 0.067213 | 0.034775 |
| **18** | 0.037428 | 0.034188 | 0.087506 | 0.00324 | 0.050078 | 0.026659 |
| **19** | -0.03855 | -0.04418 | 0.000634 | 0.00562 | 0.039187 | 0.022406 |
| **20** | -0.03594 | -0.07205 | 0.052522 | 0.03612 | 0.088460 | 0.062288 |
| **21** | -0.03886 | -0.06285 | 0.001896 | 0.02399 | 0.040751 | 0.032371 |
| **22** | -0.03344 | -0.0587 | 0.022124 | 0.02526 | 0.055563 | 0.040413 |
| **6H** | 0.034617 | 0.009736 | 0.06041 | 0.02488 | 0.025793 | 0.025337 |
| **7H** | 0.045983 | 0.015872 | 0.065012 | 0.03011 | 0.019029 | 0.024570 |
| **8H** | 0.048414 | 0.022787 | 0.06817 | 0.02563 | 0.019756 | 0.022692 |
| **12H** | 0.048729 | 0.020315 | 0.065956 | 0.02841 | 0.017227 | 0.022821 |
| **13H** | 0.050026 | 0.023992 | 0.069587 | 0.02603 | 0.019561 | 0.022798 |
| **19H** | 0.040402 | 0.033878 | 0.067 | 0.00652 | 0.026598 | 0.016561 |
| **20H** | 0.037397 | 0.014288 | 0.074512 | 0.02311 | 0.037115 | 0.030112 |
| **21H** | 0.036443 | 0.015744 | 0.065765 | 0.02070 | 0.029322 | 0.025011 |
| **22H** | 0.044471 | 0.026882 | 0.075071 | 0.01759 | 0.030600 | 0.024095 |
|  | **0.00011** | **-0.9999** | **1.000107** | **1.000005** | **0.999997** | **1.000001** |

**10b**

|  | **Atomic Charges** | | | **Fukui Indices** | | |
| --- | --- | --- | --- | --- | --- | --- |
| **Atom Number** | **Neutral** | **Anion** | **Cation** | $\boldsymbol{F}^{\boldsymbol{+}}$ | $\boldsymbol{F}^{\boldsymbol{-}}$ | $\boldsymbol{F}^{\boldsymbol{0}}$ |
| **1** | -0.30267 | -0.37872 | -0.27257 | 0.07605 | 0.030101 | 0.053075 |
| **2** | 0.229736 | 0.180285 | 0.23673 | 0.04945 | 0.006994 | 0.028223 |
| **3** | -0.02078 | -0.06546 | -0.01111 | 0.04468 | 0.009668 | 0.027173 |
| **4** | 0.008394 | 0.007156 | 0.017531 | 0.00124 | 0.009137 | 0.005187 |
| **5** | -0.00361 | -0.01047 | 0.003726 | 0.00686 | 0.007336 | 0.007099 |
| **6** | 0.051714 | 0.006779 | 0.097725 | 0.04493 | 0.046011 | 0.045473 |
| **7** | -0.05442 | -0.12701 | 0.018821 | 0.07259 | 0.073245 | 0.072915 |
| **8** | -0.03176 | -0.07031 | -0.01105 | 0.03855 | 0.020713 | 0.029630 |
| **9** | 0.001799 | -0.05448 | 0.040009 | 0.05628 | 0.038210 | 0.047243 |
| **10** | -0.01037 | -0.05562 | 0.013817 | 0.04525 | 0.024189 | 0.034718 |
| **11** | -0.00942 | -0.07213 | 0.014766 | 0.06271 | 0.024189 | 0.043448 |
| **12** | -0.02595 | -0.06715 | 0.001685 | 0.04120 | 0.027635 | 0.034417 |
| **13** | 0.006554 | -0.05618 | 0.032592 | 0.06274 | 0.026038 | 0.044388 |
| **14** | -0.05772 | -0.05803 | -0.04781 | 0.00031 | 0.009905 | 0.005108 |
| **15** | 0.130029 | 0.11153 | 0.177268 | 0.01850 | 0.047239 | 0.032869 |
| **16** | -0.23331 | -0.2933 | -0.16044 | 0.05999 | 0.072876 | 0.066431 |
| **17** | 0.032884 | 0.027191 | 0.091983 | 0.00569 | 0.059099 | 0.032396 |
| **18** | 0.041534 | 0.033642 | 0.085173 | 0.00789 | 0.043639 | 0.025765 |
| **19** | -0.03635 | -0.04464 | -0.00093 | 0.00829 | 0.035417 | 0.021854 |
| **20** | -0.03763 | -0.0729 | 0.048601 | 0.03527 | 0.086231 | 0.060750 |
| **21** | -0.03896 | -0.06264 | 0.000276 | 0.02368 | 0.039234 | 0.031457 |
| **22** | -0.03417 | -0.05942 | 0.020553 | 0.02525 | 0.054718 | 0.039985 |
| **8H** | 0.048142 | 0.020847 | 0.067143 | 0.02730 | 0.019001 | 0.023148 |
| **9H** | 0.053971 | 0.024828 | 0.070605 | 0.02914 | 0.016634 | 0.022889 |
| **11H** | 0.038719 | 0.008793 | 0.05409 | 0.02993 | 0.015371 | 0.022649 |
| **12H** | 0.045198 | 0.015518 | 0.064628 | 0.02968 | 0.019430 | 0.024555 |
| **13H** | 0.052916 | 0.021994 | 0.068228 | 0.03092 | 0.015312 | 0.023117 |
| **19H** | 0.041948 | 0.033554 | 0.066027 | 0.00839 | 0.024079 | 0.016237 |
| **20H** | 0.035359 | 0.013925 | 0.073165 | 0.02143 | 0.037806 | 0.029620 |
| **21H** | 0.034886 | 0.015733 | 0.06481 | 0.01915 | 0.029924 | 0.024539 |
| **22H** | 0.043383 | 0.026703 | 0.074012 | 0.01668 | 0.030629 | 0.023655 |
|  | **0.000053** | **-0.99996** | **1.000063** | **1.000009** | **1.00001** | **1.00001** |

**11a**

|  | **Atomic Charges** | | | **Fukui Indices** | | |
| --- | --- | --- | --- | --- | --- | --- |
| **Atom Number** | **Neutral** | **Anion** | **Cation** | $\boldsymbol{F}^{\boldsymbol{+}}$ | $\boldsymbol{F}^{\boldsymbol{-}}$ | $\boldsymbol{F}^{\boldsymbol{0}}$ |
| **1** | -0.29376 | -0.35862 | -0.26468 | 0.06486 | 0.029077 | 0.046970 |
| **2** | 0.233095 | 0.192029 | 0.2389 | 0.04107 | 0.005805 | 0.023436 |
| **3** | -0.00058 | -0.06421 | -0.00077 | 0.06363 | -0.000194 | 0.031718 |
| **4** | 0.006702 | 0.002545 | 0.018394 | 0.00416 | 0.011692 | 0.007925 |
| **5** | 0.010505 | -0.00132 | 0.017538 | 0.01183 | 0.007033 | 0.009430 |
| **6** | -0.02062 | -0.04748 | 0.034779 | 0.02686 | 0.055394 | 0.041129 |
| **7** | -0.01636 | -0.0599 | 0.003148 | 0.04354 | 0.019506 | 0.031523 |
| **8** | 0.001838 | -0.0397 | 0.03662 | 0.04154 | 0.034782 | 0.038160 |
| **9** | -0.00742 | -0.03582 | 0.014987 | 0.02840 | 0.022407 | 0.025401 |
| **10** | 0.05286 | 0.003564 | 0.076731 | 0.04930 | 0.023871 | 0.036583 |
| **11** | 0.268565 | 0.221566 | 0.241481 | 0.04700 | -0.027084 | 0.009958 |
| **12** | -0.20905 | -0.29192 | -0.17769 | 0.08287 | 0.031356 | 0.057113 |
| **13** | -0.21923 | -0.30581 | -0.14876 | 0.08658 | 0.070466 | 0.078524 |
| **14** | -0.00534 | -0.06043 | 0.006245 | 0.05509 | 0.011584 | 0.033338 |
| **15** | 0.001868 | -0.0456 | 0.027661 | 0.04747 | 0.025793 | 0.036630 |
| **16** | -0.05647 | -0.05767 | -0.04637 | 0.00121 | 0.010092 | 0.005650 |
| **17** | 0.130413 | 0.129183 | 0.182682 | 0.00123 | 0.052269 | 0.026749 |
| **18** | -0.22736 | -0.26996 | -0.15892 | 0.04260 | 0.068443 | 0.055519 |
| **19** | 0.033919 | 0.028579 | 0.099877 | 0.00534 | 0.065958 | 0.035649 |
| **20** | 0.041708 | 0.038179 | 0.088853 | 0.00353 | 0.047145 | 0.025337 |
| **21** | -0.03515 | -0.04166 | 0.002353 | 0.00651 | 0.037498 | 0.022005 |
| **22** | -0.03505 | -0.06196 | 0.05628 | 0.02690 | 0.091332 | 0.059118 |
| **23** | -0.03636 | -0.05813 | 0.004806 | 0.02176 | 0.041167 | 0.031466 |
| **24** | -0.0318 | -0.05133 | 0.024392 | 0.01953 | 0.056192 | 0.037862 |
| **6H** | 0.037925 | 0.02174 | 0.05889 | 0.01619 | 0.020965 | 0.018575 |
| **7H** | 0.048404 | 0.020997 | 0.06743 | 0.02741 | 0.019026 | 0.023217 |
| **8H** | 0.053525 | 0.029127 | 0.068662 | 0.02440 | 0.015137 | 0.019768 |
| **14H** | 0.054663 | 0.026717 | 0.066784 | 0.02795 | 0.012121 | 0.020034 |
| **15H** | 0.056806 | 0.029313 | 0.070514 | 0.02749 | 0.013708 | 0.020601 |
| **21H** | 0.042933 | 0.036766 | 0.068342 | 0.00617 | 0.025409 | 0.015788 |
| **22H** | 0.037036 | 0.019808 | 0.076364 | 0.01723 | 0.039328 | 0.028278 |
| **23H** | 0.036576 | 0.019858 | 0.067548 | 0.01672 | 0.030972 | 0.023845 |
| **24H** | 0.045033 | 0.031364 | 0.076788 | 0.01367 | 0.031755 | 0.022712 |
|  | **-0.00015** | **-1.00017** | **0.999852** | **1.00001** | **1.00001** | **1.00001** |

**11b**

|  | **Atomic Charges** | | | **Fukui Indices** | | |
| --- | --- | --- | --- | --- | --- | --- |
| **Atom Number** | **Neutral** | **Anion** | **Cation** | $\boldsymbol{F}^{\boldsymbol{+}}$ | $\boldsymbol{F}^{\boldsymbol{-}}$ | $\boldsymbol{F}^{\boldsymbol{0}}$ |
| **1** | -0.29762 | -0.3572 | -0.26811 | 0.05957 | 0.029512 | 0.044543 |
| **2** | 0.231177 | 0.200844 | 0.237763 | 0.03033 | 0.006586 | 0.018460 |
| **3** | -0.01965 | -0.05215 | -0.0129 | 0.03250 | 0.006746 | 0.019624 |
| **4** | 0.007723 | 0.005634 | 0.017733 | 0.00209 | 0.010010 | 0.006050 |
| **5** | 0.009165 | -0.0001 | 0.013544 | 0.00927 | 0.004379 | 0.006822 |
| **6** | 0.049069 | 0.003882 | 0.095201 | 0.04519 | 0.046132 | 0.045660 |
| **7** | 0.266958 | 0.240838 | 0.236361 | 0.02612 | -0.030597 | -0.002239 |
| **8** | -0.21918 | -0.27591 | -0.17184 | 0.05673 | 0.047341 | 0.052035 |
| **9** | -0.22335 | -0.28792 | -0.15076 | 0.06456 | 0.072594 | 0.068578 |
| **10** | -0.00622 | -0.05742 | 0.0011 | 0.05121 | 0.007315 | 0.029260 |
| **11** | -0.00125 | -0.04994 | 0.034998 | 0.04869 | 0.036243 | 0.042469 |
| **12** | 0.008324 | -0.05273 | 0.016687 | 0.06105 | 0.008363 | 0.034708 |
| **13** | -0.00749 | -0.05057 | 0.014501 | 0.04309 | 0.021986 | 0.032537 |
| **14** | -0.01845 | -0.06038 | 0.004067 | 0.04193 | 0.022519 | 0.032223 |
| **15** | 0.008624 | -0.04383 | 0.031488 | 0.05246 | 0.022864 | 0.037660 |
| **16** | -0.05655 | -0.0593 | -0.04533 | 0.00274 | 0.011222 | 0.006982 |
| **17** | 0.128164 | 0.113594 | 0.185873 | 0.01457 | 0.057709 | 0.036140 |
| **18** | -0.22398 | -0.28172 | -0.16285 | 0.05774 | 0.061135 | 0.059438 |
| **19** | 0.033567 | 0.029723 | 0.10636 | 0.00384 | 0.072793 | 0.038318 |
| **20** | 0.044034 | 0.034649 | 0.092925 | 0.00938 | 0.048891 | 0.029138 |
| **21** | -0.03491 | -0.04448 | 0.004406 | 0.00957 | 0.039320 | 0.024445 |
| **22** | -0.03308 | -0.06792 | 0.061743 | 0.03484 | 0.094823 | 0.064830 |
| **23** | -0.03654 | -0.05871 | 0.007003 | 0.02217 | 0.043540 | 0.032856 |
| **24** | -0.03082 | -0.05515 | 0.02552 | 0.02433 | 0.056341 | 0.040334 |
| **10H** | 0.056408 | 0.028483 | 0.069814 | 0.02793 | 0.013406 | 0.020666 |
| **11H** | 0.056293 | 0.028653 | 0.070245 | 0.02764 | 0.013952 | 0.020796 |
| **13H** | 0.042636 | 0.021439 | 0.055978 | 0.02120 | 0.013342 | 0.017270 |
| **14H** | 0.04899 | 0.020786 | 0.066016 | 0.02820 | 0.017026 | 0.022615 |
| **15H** | 0.054648 | 0.027292 | 0.068036 | 0.02736 | 0.013388 | 0.020372 |
| **21H** | 0.043426 | 0.034409 | 0.06967 | 0.00902 | 0.026244 | 0.017631 |
| **22H** | 0.037626 | 0.016788 | 0.078036 | 0.02084 | 0.040410 | 0.030624 |
| **23H** | 0.036774 | 0.018566 | 0.068755 | 0.01821 | 0.031981 | 0.025095 |
| **24H** | 0.045403 | 0.029747 | 0.077908 | 0.01566 | 0.032505 | 0.024081 |
|  | **-0.000083** | **-1.00009** | **0.999938** | **1.00001** | **1.00002** | **1.00002** |

**13**

|  | **Atomic Charges** | | | **Fukui Indices** | | |
| --- | --- | --- | --- | --- | --- | --- |
| **Atom Number** | **Neutral** | **Anion** | **Cation** | $\boldsymbol{F}^{\boldsymbol{+}}$ | $\boldsymbol{F}^{\boldsymbol{-}}$ | $\boldsymbol{F}^{\boldsymbol{0}}$ |
| **1** | -0.27692 | -0.39232 | -0.28722 | 0.11540 | -0.010293 | 0.052552 |
| **2** | 0.179901 | 0.177221 | 0.223677 | 0.00268 | 0.043776 | 0.023228 |
| **3** | -0.01658 | -0.06286 | -0.00815 | 0.04628 | 0.008422 | 0.027351 |
| **4** | -0.00082 | 0.003437 | 0.010263 | -0.00425 | 0.011080 | 0.003413 |
| **5** | 0.003052 | -0.00833 | 0.007781 | 0.01139 | 0.004729 | 0.008057 |
| **6** | -0.02589 | -0.07889 | 0.035453 | 0.05300 | 0.061345 | 0.057172 |
| **7** | -0.02816 | -0.07144 | -0.01105 | 0.04328 | 0.017101 | 0.030192 |
| **8** | -0.01635 | -0.06388 | 0.030148 | 0.04753 | 0.046498 | 0.047016 |
| **9** | -0.0074 | -0.0584 | 0.003892 | 0.05099 | 0.011296 | 0.031144 |
| **10** | 0.12569 | 0.119439 | 0.194564 | 0.00625 | 0.068874 | 0.037562 |
| **11** | -0.19776 | -0.2895 | -0.13726 | 0.09175 | 0.060497 | 0.076121 |
| **12** | 0.033884 | 0.040737 | 0.084715 | -0.00685 | 0.050831 | 0.021989 |
| **13** | -0.03395 | -0.05793 | 0.008277 | 0.02398 | 0.042228 | 0.033103 |
| **14** | -0.03679 | -0.05678 | 0.001086 | 0.01999 | 0.037879 | 0.028935 |
| **15** | -0.03658 | -0.07895 | 0.031281 | 0.04238 | 0.067857 | 0.055117 |
| **16** | -0.03814 | -0.0509 | -0.01068 | 0.01275 | 0.027468 | 0.020110 |
| **17** | -0.00694 | -0.02142 | 0.030637 | 0.01448 | 0.037580 | 0.026030 |
| **18** | 0.022387 | 0.025204 | 0.043072 | -0.00282 | 0.020685 | 0.008934 |
| **19** | -0.05274 | -0.07427 | -0.0464 | 0.02153 | 0.006342 | 0.013937 |
| **20** | -0.0219 | -0.0712 | 0.017648 | 0.04930 | 0.039545 | 0.044425 |
| **21** | -0.02795 | -0.0723 | -0.00289 | 0.04435 | 0.025061 | 0.034707 |
| **22** | -0.01433 | -0.06131 | 0.030043 | 0.04699 | 0.044369 | 0.045679 |
| **6H** | 0.040177 | 0.009489 | 0.066914 | 0.03069 | 0.026737 | 0.028713 |
| **7H** | 0.041807 | 0.011508 | 0.060343 | 0.03030 | 0.018536 | 0.024418 |
| **8H** | 0.039503 | 0.014518 | 0.057944 | 0.02498 | 0.018441 | 0.021713 |
| **13H** | 0.038479 | 0.0245 | 0.06247 | 0.01398 | 0.023991 | 0.018985 |
| **14H** | 0.037167 | 0.018174 | 0.063342 | 0.01899 | 0.026175 | 0.022584 |
| **15H** | 0.036638 | 0.011512 | 0.068202 | 0.02513 | 0.031564 | 0.028345 |
| **16H** | 0.037494 | 0.023335 | 0.059842 | 0.01416 | 0.022348 | 0.018254 |
| **18H1** | 0.037409 | 0.024635 | 0.06093 | 0.01277 | 0.023521 | 0.018148 |
| **18H2** | 0.037318 | 0.024402 | 0.060949 | 0.01292 | 0.023631 | 0.018274 |
| **20H** | 0.04103 | 0.01171 | 0.061518 | 0.02932 | 0.020488 | 0.024904 |
| **21H** | 0.042407 | 0.011767 | 0.061814 | 0.03064 | 0.019407 | 0.025024 |
| **22H** | 0.044716 | 0.018968 | 0.066716 | 0.02575 | 0.022000 | 0.023874 |
|  | **-0.00014** | **-1.00014** | **0.99987** | **1.00000** | **1.00001** | **1.00001** |

**OH-3**

|  | **Atomic Charges** | | | **Fukui Indices** | | |
| --- | --- | --- | --- | --- | --- | --- |
| **Atom Number** | **Neutral** | **Anion** | **Cation** | $\boldsymbol{F}^{\boldsymbol{+}}$ | $\boldsymbol{F}^{\boldsymbol{-}}$ | $\boldsymbol{F}^{\boldsymbol{0}}$ |
| **1** | -0.25501 | -0.38565 | -0.28722 | 0.13064 | -0.020157 | 0.055243 |
| **2** | 0.194791 | 0.170114 | 0.223677 | 0.02468 | 0.041281 | 0.032979 |
| **3** | -0.01595 | -0.06992 | -0.00815 | 0.05397 | 0.004775 | 0.029373 |
| **4** | 0.000548 | 0.006331 | 0.010263 | -0.00578 | 0.017120 | 0.005668 |
| **5** | -0.00725 | -0.0209 | 0.007781 | 0.01365 | 0.003422 | 0.008538 |
| **6** | 0.094825 | 0.064997 | 0.035453 | 0.02983 | 0.075515 | 0.052672 |
| **7** | -0.17615 | -0.22964 | -0.01105 | 0.05349 | 0.017814 | 0.035652 |
| **8** | -0.05737 | -0.09311 | 0.030148 | 0.03574 | 0.021773 | 0.028757 |
| **9** | -0.0109 | -0.05395 | 0.003892 | 0.04305 | 0.058104 | 0.050578 |
| **10** | -0.01935 | -0.05904 | 0.194564 | 0.03969 | 0.026535 | 0.033112 |
| **11** | -0.01438 | -0.073 | -0.13726 | 0.05862 | 0.038706 | 0.048664 |
| **12** | -0.02433 | -0.0689 | 0.084715 | 0.04456 | 0.024365 | 0.034464 |
| **13** | -0.00733 | -0.05997 | 0.008277 | 0.05264 | 0.043378 | 0.048008 |
| **14** | -0.03773 | -0.05707 | 0.001086 | 0.01934 | -0.012233 | 0.003556 |
| **15** | 0.108013 | 0.113228 | 0.031281 | -0.00522 | 0.057867 | 0.026326 |
| **16** | -0.21487 | -0.29634 | -0.01068 | 0.08147 | 0.049243 | 0.065357 |
| **17** | 0.027504 | 0.025973 | 0.030637 | 0.00153 | 0.051340 | 0.026436 |
| **18** | 0.040256 | 0.038941 | 0.043072 | 0.00131 | 0.041340 | 0.021327 |
| **19** | -0.04347 | -0.04372 | -0.0464 | 0.00025 | 0.031708 | 0.015979 |
| **20** | 0.031645 | 0.009245 | 0.017648 | 0.02240 | 0.060586 | 0.041493 |
| **21** | -0.0763 | -0.13939 | -0.00289 | 0.06309 | 0.079692 | 0.071391 |
| **22** | -0.04351 | -0.06387 | 0.030043 | 0.02036 | 0.035009 | 0.027683 |
| **23** | -0.02877 | -0.05302 | 0.066914 | 0.02425 | 0.047124 | 0.035688 |
| **7H** | 0.177244 | 0.162376 | 0.060343 | 0.01487 | 0.026950 | 0.020909 |
| **8H** | 0.04009 | 0.012209 | 0.057944 | 0.02788 | 0.021019 | 0.024450 |
| **9H** | 0.048602 | 0.023291 | 0.06247 | 0.02531 | 0.024067 | 0.024689 |
| **11H** | 0.043321 | 0.012614 | 0.063342 | 0.03071 | 0.020567 | 0.025637 |
| **12H** | 0.045105 | 0.013747 | 0.068202 | 0.03136 | 0.019213 | 0.025286 |
| **13H** | 0.047557 | 0.020224 | 0.059842 | 0.02733 | 0.021718 | 0.024526 |
| **19H** | 0.044706 | 0.040798 | 0.06093 | 0.00391 | 0.021723 | 0.012816 |
| **22H** | 0.040863 | 0.022824 | 0.060949 | 0.01804 | 0.024509 | 0.021274 |
| **23H** | 0.047545 | 0.030531 | 0.061518 | 0.01701 | 0.025920 | 0.021467 |
|  | **-0.000053** | **-1.00005** | **0.99994** | **0.999996** | **0.999993** | **0.999994** |

**OH-4**

|  | **Atomic Charges** | | | **Fukui Indices** | | |
| --- | --- | --- | --- | --- | --- | --- |
| **Atom Number** | **Neutral** | **Anion** | **Cation** | $\boldsymbol{F}^{\boldsymbol{+}}$ | $\boldsymbol{F}^{\boldsymbol{-}}$ | $\boldsymbol{F}^{\boldsymbol{0}}$ |
| **1** | -0.25967 | -0.38842 | -0.28064 | 0.12875 | -0.020969 | 0.053889 |
| **2** | 0.194647 | 0.172415 | 0.236853 | 0.02223 | 0.042206 | 0.032219 |
| **3** | -0.01716 | -0.06842 | -0.02113 | 0.05126 | -0.003970 | 0.023642 |
| **4** | -0.00082 | 0.003548 | 0.013787 | -0.00437 | 0.014610 | 0.005119 |
| **5** | 0.004421 | -0.00728 | 0.008739 | 0.01170 | 0.004318 | 0.008007 |
| **6** | -0.02487 | -0.07089 | 0.025601 | 0.04602 | 0.050473 | 0.048246 |
| **7** | -0.02494 | -0.06823 | -0.01044 | 0.04329 | 0.014502 | 0.028897 |
| **8** | -0.01222 | -0.05932 | 0.026785 | 0.04710 | 0.039008 | 0.043052 |
| **9** | -0.00545 | -0.05148 | -0.00201 | 0.04603 | 0.003448 | 0.024739 |
| **10** | -0.01543 | -0.07322 | 0.013068 | 0.05779 | 0.028500 | 0.043143 |
| **11** | -0.0257 | -0.06981 | -0.00883 | 0.04411 | 0.016869 | 0.030490 |
| **12** | -0.00912 | -0.06146 | 0.023872 | 0.05234 | 0.032994 | 0.042666 |
| **13** | -0.03777 | -0.05679 | -0.04604 | 0.01902 | -0.008263 | 0.005377 |
| **14** | 0.105524 | 0.110561 | 0.177628 | -0.00504 | 0.072104 | 0.033534 |
| **15** | -0.20833 | -0.29308 | -0.19131 | 0.08475 | 0.017021 | 0.050884 |
| **16** | 0.022515 | 0.018069 | 0.102212 | 0.00445 | 0.079697 | 0.042072 |
| **17** | 0.036832 | 0.039561 | 0.109432 | -0.00273 | 0.072600 | 0.034936 |
| **18** | -0.06358 | -0.0684 | -0.03424 | 0.00482 | 0.029339 | 0.017082 |
| **19** | 0.079556 | 0.0664 | 0.174067 | 0.01316 | 0.094511 | 0.053834 |
| **20** | -0.1873 | -0.23481 | -0.14357 | 0.04751 | 0.043724 | 0.045619 |
| **21** | 0.016909 | 0.002651 | 0.07825 | 0.01426 | 0.061341 | 0.037799 |
| **22** | -0.06714 | -0.12575 | 0.022923 | 0.05861 | 0.090067 | 0.074336 |
| **23** | -0.03455 | -0.0503 | -0.00151 | 0.01575 | 0.033034 | 0.024392 |
| **6H** | 0.041383 | 0.013232 | 0.0641 | 0.02815 | 0.022717 | 0.025434 |
| **7H** | 0.044334 | 0.014297 | 0.060109 | 0.03004 | 0.015775 | 0.022906 |
| **8H** | 0.047405 | 0.021146 | 0.063239 | 0.02626 | 0.015834 | 0.021047 |
| **10H** | 0.043706 | 0.01155 | 0.060116 | 0.03216 | 0.016410 | 0.024283 |
| **11H** | 0.044645 | 0.013551 | 0.059654 | 0.03109 | 0.015009 | 0.023052 |
| **12H** | 0.046545 | 0.019406 | 0.062973 | 0.02714 | 0.016428 | 0.021784 |
| **18H** | 0.039361 | 0.033903 | 0.065762 | 0.00546 | 0.026401 | 0.015930 |
| **20H** | 0.17471 | 0.169902 | 0.210748 | 0.00481 | 0.036038 | 0.020423 |
| **23H** | 0.051449 | 0.037336 | 0.079675 | 0.01411 | 0.028226 | 0.021170 |
|  | **-0.00013** | **-1.00013** | **0.999872** | **1.00000** | **1.00000** | **1.00000** |

**OH-5**

|  | **Atomic Charges** | | | **Fukui Indices** | | |
| --- | --- | --- | --- | --- | --- | --- |
| **Atom Number** | **Neutral** | **Anion** | **Cation** | $\boldsymbol{F}^{\boldsymbol{+}}$ | $\boldsymbol{F}^{\boldsymbol{-}}$ | $\boldsymbol{F}^{\boldsymbol{0}}$ |
| **1** | -0.26048 | -0.38939 | -0.28108 | 0.12891 | -0.020601 | 0.054156 |
| **2** | 0.194294 | 0.172005 | 0.236271 | 0.02229 | 0.041977 | 0.032133 |
| **3** | -0.01711 | -0.07003 | -0.01974 | 0.05292 | -0.002629 | 0.025147 |
| **4** | -0.00108 | 0.002806 | 0.01355 | -0.00388 | 0.014625 | 0.005372 |
| **5** | 0.003916 | -0.00773 | 0.008761 | 0.01165 | 0.004845 | 0.008245 |
| **6** | -0.02654 | -0.07274 | 0.026548 | 0.04620 | 0.053090 | 0.049644 |
| **7** | -0.026 | -0.06993 | -0.01025 | 0.04394 | 0.015748 | 0.029842 |
| **8** | -0.01368 | -0.06072 | 0.027186 | 0.04704 | 0.040869 | 0.043954 |
| **9** | -0.00531 | -0.05141 | -0.00013 | 0.04609 | 0.005187 | 0.025641 |
| **10** | -0.01654 | -0.07582 | 0.012798 | 0.05928 | 0.029340 | 0.044309 |
| **11** | -0.02664 | -0.07152 | -0.00821 | 0.04489 | 0.018429 | 0.031659 |
| **12** | -0.01022 | -0.06317 | 0.024008 | 0.05295 | 0.034227 | 0.043588 |
| **13** | -0.03846 | -0.05774 | -0.04744 | 0.01928 | -0.008981 | 0.005149 |
| **14** | 0.102788 | 0.108893 | 0.178031 | -0.00610 | 0.075243 | 0.034569 |
| **15** | -0.20994 | -0.29445 | -0.18598 | 0.08452 | 0.023957 | 0.054237 |
| **16** | 0.018624 | 0.01271 | 0.101035 | 0.00591 | 0.082411 | 0.044163 |
| **17** | 0.038376 | 0.039368 | 0.101704 | -0.00099 | 0.063328 | 0.031168 |
| **18** | -0.06859 | -0.07509 | -0.03026 | 0.00649 | 0.038333 | 0.022412 |
| **19** | 0.082871 | 0.066734 | 0.176924 | 0.01614 | 0.094053 | 0.055095 |
| **20** | -0.19753 | -0.24638 | -0.15018 | 0.04885 | 0.047347 | 0.048099 |
| **21** | -0.05174 | -0.07879 | 0.009676 | 0.02705 | 0.061412 | 0.044231 |
| **22** | -0.02909 | -0.04908 | 0.019114 | 0.01999 | 0.048200 | 0.034096 |
| **6H** | 0.040523 | 0.012151 | 0.06435 | 0.02837 | 0.023827 | 0.026100 |
| **7H** | 0.043459 | 0.013005 | 0.06017 | 0.03045 | 0.016711 | 0.023583 |
| **8H** | 0.046558 | 0.020104 | 0.063239 | 0.02645 | 0.016681 | 0.021568 |
| **10H** | 0.043002 | 0.010146 | 0.060004 | 0.03286 | 0.017002 | 0.024929 |
| **11H** | 0.043906 | 0.012308 | 0.059758 | 0.03160 | 0.015852 | 0.023725 |
| **12H** | 0.045898 | 0.018362 | 0.063084 | 0.02754 | 0.017186 | 0.022361 |
| **18H** | 0.036349 | 0.030076 | 0.064732 | 0.00627 | 0.028383 | 0.017328 |
| **20H** | 0.170187 | 0.164751 | 0.206613 | 0.00544 | 0.036426 | 0.020931 |
| **21H** | 0.041729 | 0.020897 | 0.076719 | 0.02083 | 0.034990 | 0.027911 |
| **22H** | 0.046341 | 0.029569 | 0.078874 | 0.01677 | 0.032533 | 0.024653 |
|  | **-0.00012** | **-1.0001** | **0.999886** | **0.999988** | **1.000001** | **0.999994** |

**OH-10b**

|  | **Atomic Charges** | | | **Fukui Indices** | | |
| --- | --- | --- | --- | --- | --- | --- |
| **Atom Number** | **Neutral** | **Anion** | **Cation** | $\boldsymbol{F}^{\boldsymbol{+}}$ | $\boldsymbol{F}^{\boldsymbol{-}}$ | $\boldsymbol{F}^{\boldsymbol{0}}$ |
| **1** | -0.25842 | -0.3826 | -0.2796 | 0.12418 | -0.021179 | 0.051499 |
| **2** | 0.194948 | 0.178059 | 0.23664 | 0.01689 | 0.041692 | 0.029291 |
| **3** | -0.01519 | -0.06788 | -0.01868 | 0.05269 | -0.003494 | 0.024598 |
| **4** | 0.002125 | 0.006816 | 0.016643 | -0.00469 | 0.014518 | 0.004914 |
| **5** | -0.00107 | -0.01038 | 0.003383 | 0.00931 | 0.004454 | 0.006881 |
| **6** | 0.039683 | 0.006846 | 0.084907 | 0.03284 | 0.045224 | 0.039031 |
| **7** | -0.04553 | -0.12699 | 0.00303 | 0.08146 | 0.048559 | 0.065008 |
| **8** | -0.0305 | -0.0704 | -0.01568 | 0.03990 | 0.014823 | 0.027359 |
| **9** | -0.01025 | -0.05478 | 0.029623 | 0.04453 | 0.039874 | 0.042202 |
| **10** | -0.00655 | -0.05442 | -0.00143 | 0.04786 | 0.005128 | 0.026495 |
| **11** | -0.01809 | -0.07376 | 0.010841 | 0.05567 | 0.028933 | 0.042302 |
| **12** | -0.02182 | -0.0676 | -0.00455 | 0.04577 | 0.017274 | 0.031523 |
| **13** | -0.00773 | -0.05792 | 0.025998 | 0.05019 | 0.033725 | 0.041959 |
| **14** | -0.0381 | -0.05758 | -0.04723 | 0.01948 | -0.009136 | 0.005172 |
| **15** | 0.102338 | 0.109434 | 0.176641 | -0.00710 | 0.074303 | 0.033603 |
| **16** | -0.20904 | -0.29242 | -0.18604 | 0.08339 | 0.022999 | 0.053193 |
| **17** | 0.019162 | 0.013737 | 0.100879 | 0.00542 | 0.081717 | 0.043571 |
| **18** | 0.038694 | 0.039585 | 0.101574 | -0.00089 | 0.062880 | 0.030995 |
| **19** | -0.06808 | -0.07467 | -0.03012 | 0.00659 | 0.037969 | 0.022278 |
| **20** | 0.083804 | 0.068163 | 0.177346 | 0.01564 | 0.093542 | 0.054591 |
| **21** | -0.19652 | -0.24507 | -0.14954 | 0.04854 | 0.046981 | 0.047762 |
| **22** | -0.05074 | -0.07709 | 0.010257 | 0.02635 | 0.060996 | 0.043674 |
| **23** | -0.02832 | -0.0478 | 0.0192 | 0.01948 | 0.047517 | 0.033501 |
| **8H** | 0.048161 | 0.020791 | 0.06378 | 0.02737 | 0.015619 | 0.021495 |
| **9H** | 0.050073 | 0.024781 | 0.066208 | 0.02529 | 0.016135 | 0.020714 |
| **11H** | 0.03647 | 0.008229 | 0.052207 | 0.02824 | 0.015737 | 0.021989 |
| **12H** | 0.046255 | 0.01522 | 0.061458 | 0.03104 | 0.015203 | 0.023119 |
| **13H** | 0.047389 | 0.021044 | 0.064261 | 0.02635 | 0.016872 | 0.021609 |
| **19H** | 0.036827 | 0.030583 | 0.064974 | 0.00624 | 0.028147 | 0.017196 |
| **21H** | 0.170703 | 0.165405 | 0.206922 | 0.00530 | 0.036219 | 0.020759 |
| **22H** | 0.042382 | 0.022051 | 0.077025 | 0.02033 | 0.034643 | 0.027487 |
| **23H** | 0.046962 | 0.030623 | 0.079101 | 0.01634 | 0.032139 | 0.024239 |
|  | **0.000026** | **-0.99997** | **1.000039** | **0.999995** | **1.000013** | **1.000004** |

**OH-11b**

|  | **Atomic Charges** | | | **Fukui Indices** | | |
| --- | --- | --- | --- | --- | --- | --- |
| **Atom Number** | **Neutral** | **Anion** | **Cation** | $\boldsymbol{F}^{\boldsymbol{+}}$ | $\boldsymbol{F}^{\boldsymbol{-}}$ | $\boldsymbol{F}^{\boldsymbol{0}}$ |
| **1** | -0.2543 | -0.35959 | -0.27487 | 0.10529 | -0.020571 | 0.042359 |
| **2** | 0.196491 | 0.20116 | 0.237767 | -0.00467 | 0.041276 | 0.018304 |
| **3** | -0.01508 | -0.05283 | -0.01819 | 0.03775 | -0.003107 | 0.017321 |
| **4** | 0.004272 | 0.005396 | 0.016439 | -0.00112 | 0.012167 | 0.005521 |
| **5** | 0.007177 | 0.000354 | 0.015473 | 0.00682 | 0.008296 | 0.007560 |
| **6** | 0.04106 | 0.002172 | 0.084117 | 0.03889 | 0.043057 | 0.040973 |
| **7** | 0.225063 | 0.238685 | 0.271146 | -0.01362 | 0.046083 | 0.016231 |
| **8** | -0.18325 | -0.27857 | -0.19729 | 0.09532 | -0.014041 | 0.040639 |
| **9** | -0.18826 | -0.29093 | -0.19501 | 0.10267 | -0.006751 | 0.047960 |
| **10** | -0.01579 | -0.05745 | 0.00373 | 0.04166 | 0.019522 | 0.030592 |
| **11** | -0.00797 | -0.05097 | 0.025535 | 0.04299 | 0.033505 | 0.038250 |
| **12** | 0.004908 | -0.05175 | 0.009135 | 0.05666 | 0.004227 | 0.030443 |
| **13** | -0.01486 | -0.05023 | 0.01183 | 0.03538 | 0.026685 | 0.031032 |
| **14** | -0.0171 | -0.06077 | 0.000325 | 0.04367 | 0.017425 | 0.030550 |
| **15** | -0.00428 | -0.04464 | 0.025694 | 0.04036 | 0.029976 | 0.035169 |
| **16** | -0.03698 | -0.0588 | -0.04525 | 0.02182 | -0.008270 | 0.006778 |
| **17** | 0.101678 | 0.110257 | 0.178583 | -0.00858 | 0.076905 | 0.034163 |
| **18** | -0.20316 | -0.27942 | -0.18293 | 0.07625 | 0.020231 | 0.048242 |
| **19** | 0.020868 | 0.016257 | 0.107974 | 0.00461 | 0.087106 | 0.045858 |
| **20** | 0.040611 | 0.040469 | 0.107788 | 0.00014 | 0.067177 | 0.033660 |
| **21** | -0.06683 | -0.07454 | -0.02893 | 0.00771 | 0.037901 | 0.022806 |
| **22** | 0.086876 | 0.072083 | 0.186679 | 0.01479 | 0.099803 | 0.057298 |
| **23** | -0.19319 | -0.24139 | -0.14016 | 0.04820 | 0.053023 | 0.050614 |
| **24** | -0.04793 | -0.07328 | 0.020428 | 0.02535 | 0.068359 | 0.046856 |
| **25** | -0.02583 | -0.04384 | 0.020146 | 0.01801 | 0.045975 | 0.031993 |
| **10H** | 0.052295 | 0.02829 | 0.0689 | 0.02400 | 0.016605 | 0.020305 |
| **11H** | 0.052836 | 0.028389 | 0.067089 | 0.02445 | 0.014253 | 0.019350 |
| **13H** | 0.039149 | 0.02163 | 0.05484 | 0.01752 | 0.015691 | 0.016605 |
| **14H** | 0.049156 | 0.02066 | 0.063943 | 0.02850 | 0.014787 | 0.021642 |
| **15H** | 0.049295 | 0.026701 | 0.064784 | 0.02259 | 0.015489 | 0.019042 |
| **21H** | 0.038145 | 0.0313 | 0.067276 | 0.00684 | 0.029131 | 0.017988 |
| **23H** | 0.172348 | 0.166999 | 0.210873 | 0.00535 | 0.038525 | 0.021937 |
| **24H** | 0.044148 | 0.024845 | 0.080799 | 0.01930 | 0.036651 | 0.027977 |
| **25H** | 0.048738 | 0.033654 | 0.08164 | 0.01508 | 0.032902 | 0.023993 |
|  | **0.00031** | **-0.99971** | **1.00031** | **1.00002** | **0.99999** | **1.00001** |

**Fukui plots for FICZ, 8-OH FICZ, 3, and 11b visualised at 0.0135 contour value:**

|  | **FICZ** |
| --- | --- |
| $\boldsymbol{F}^{\boldsymbol{+}}$ | **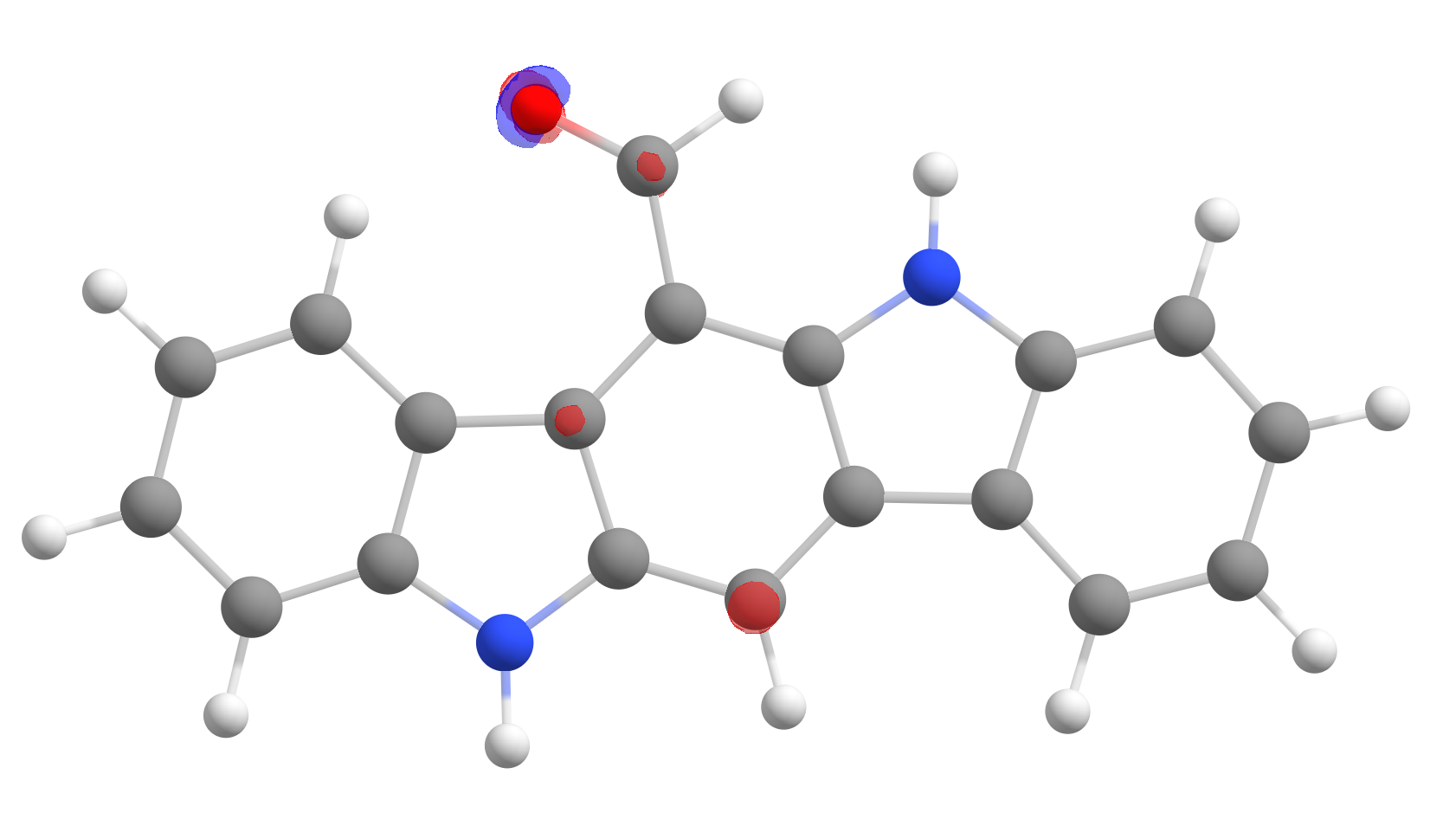** |
| $\boldsymbol{F}^{\boldsymbol{-}}$ | **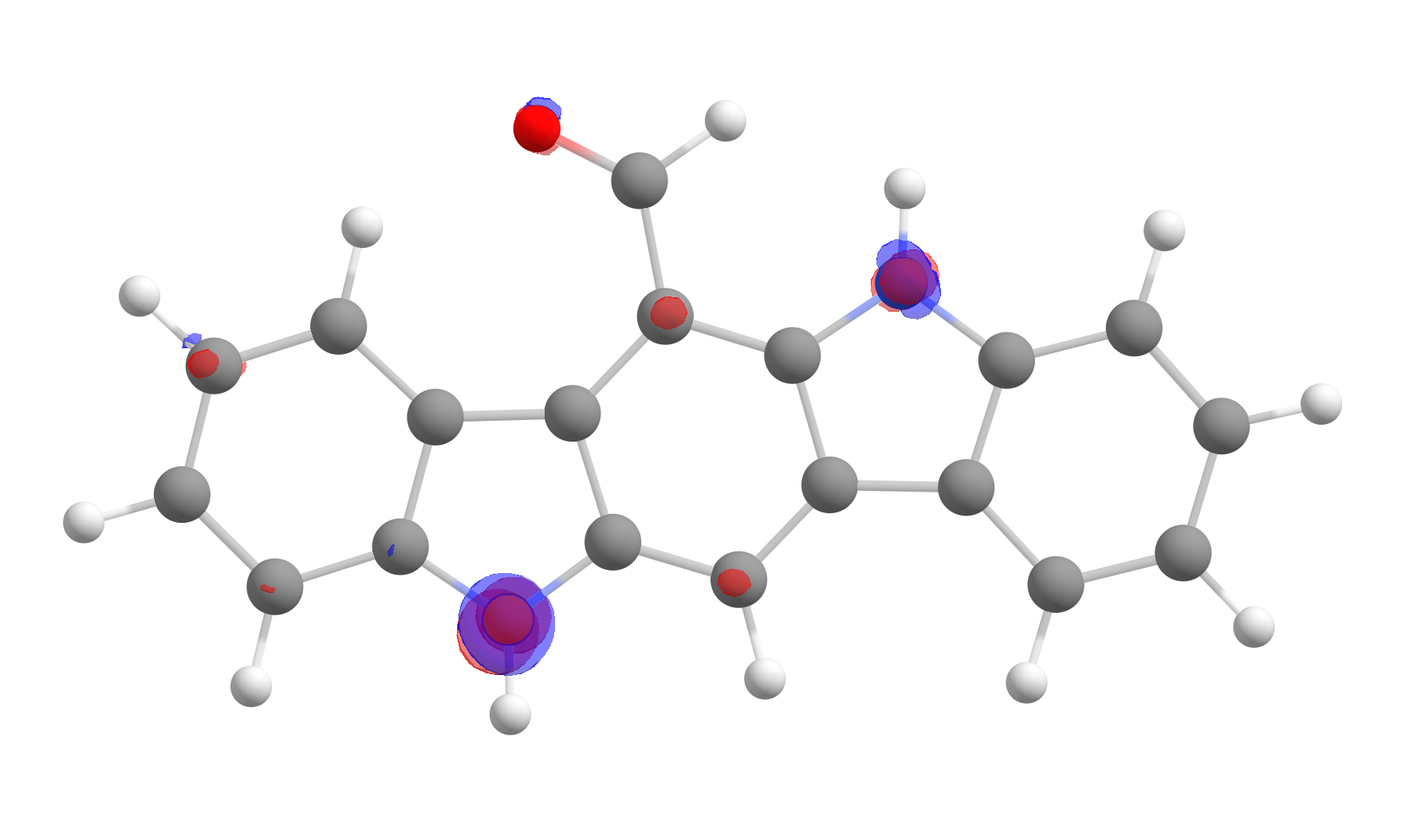** |
| $\boldsymbol{F}^{\boldsymbol{0}}$ | **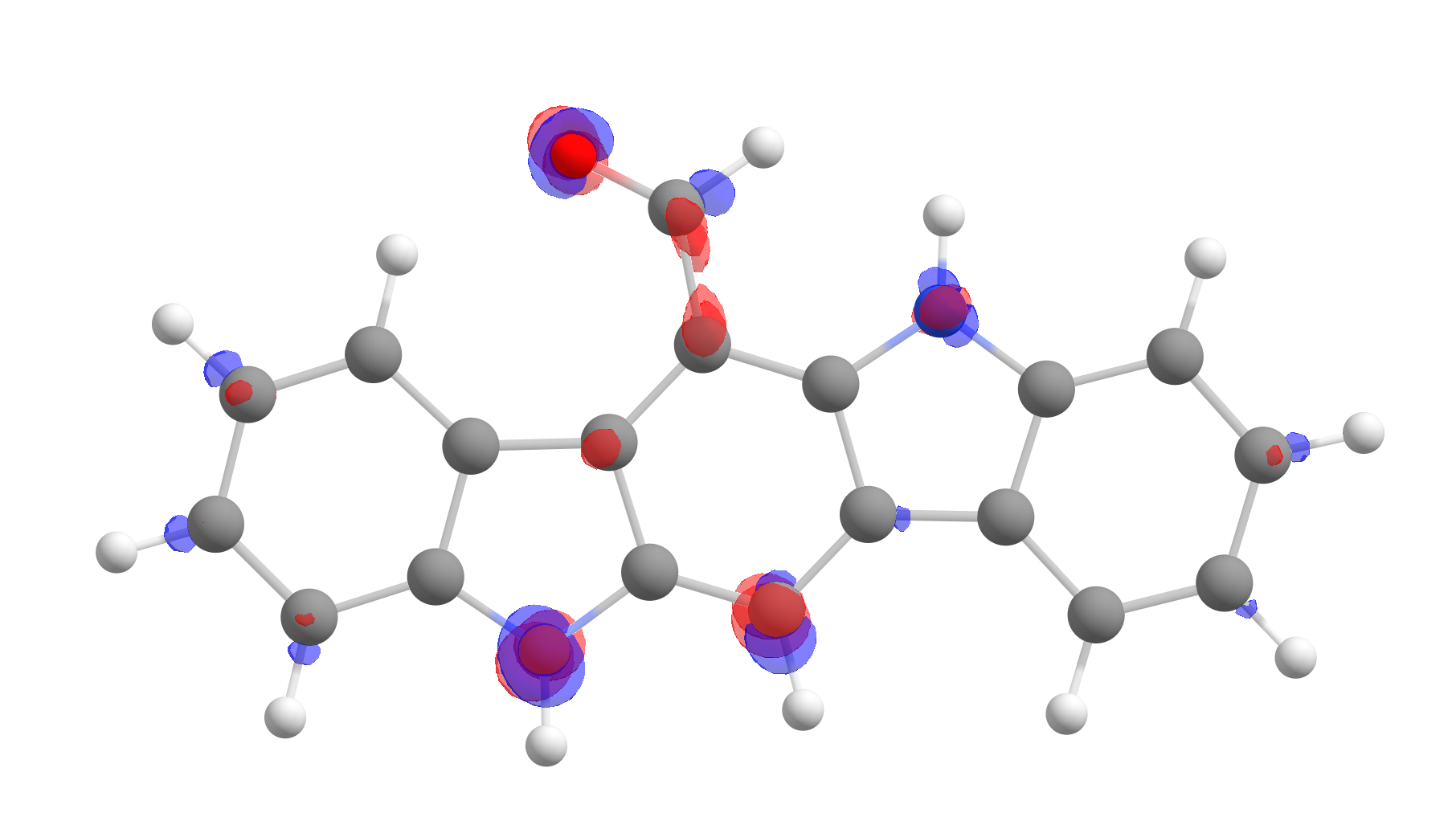** |

|  | **8-OH FICZ** |
| --- | --- |
| $\boldsymbol{F}^{\boldsymbol{+}}$ | **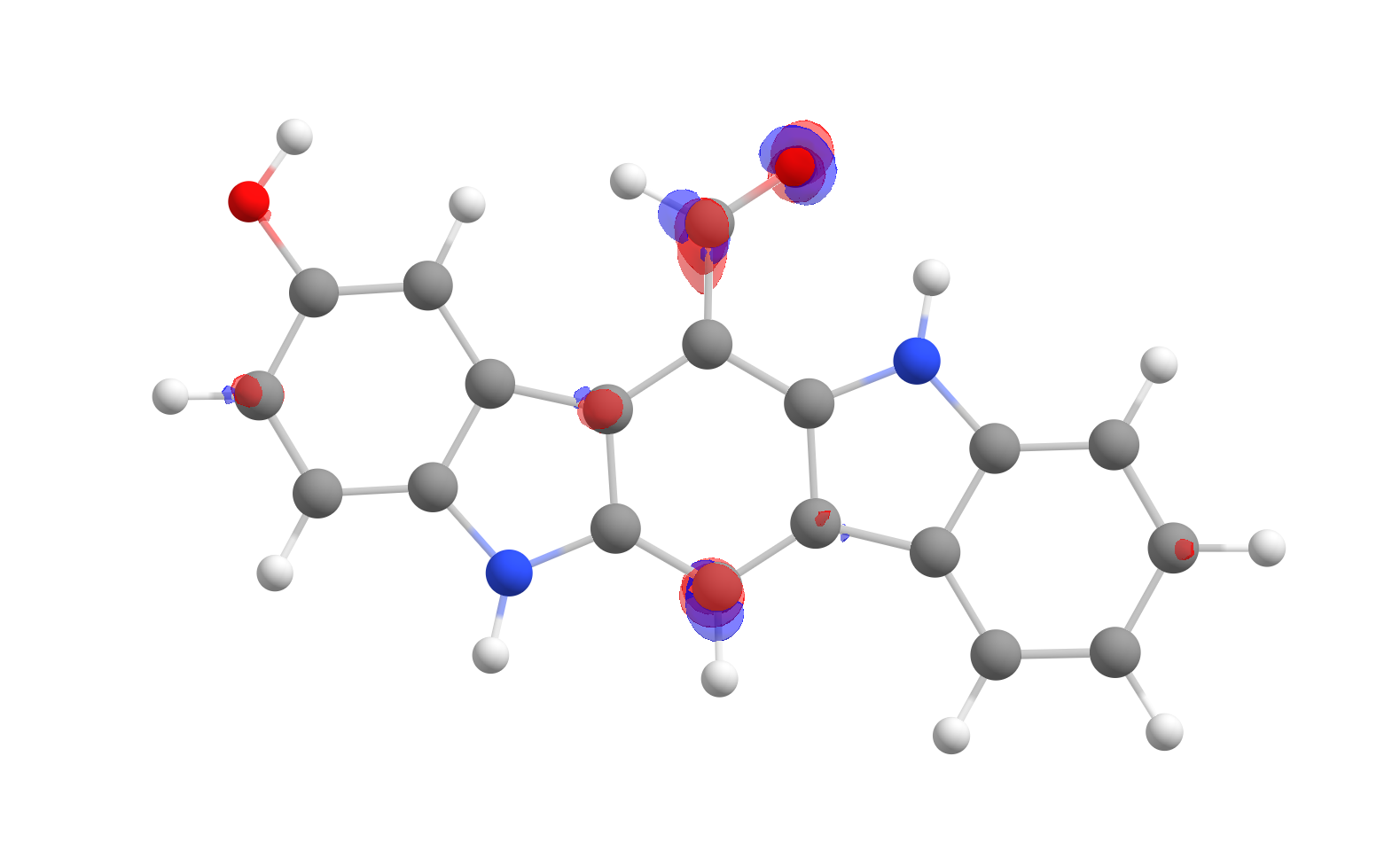** |
| $\boldsymbol{F}^{\boldsymbol{-}}$ | **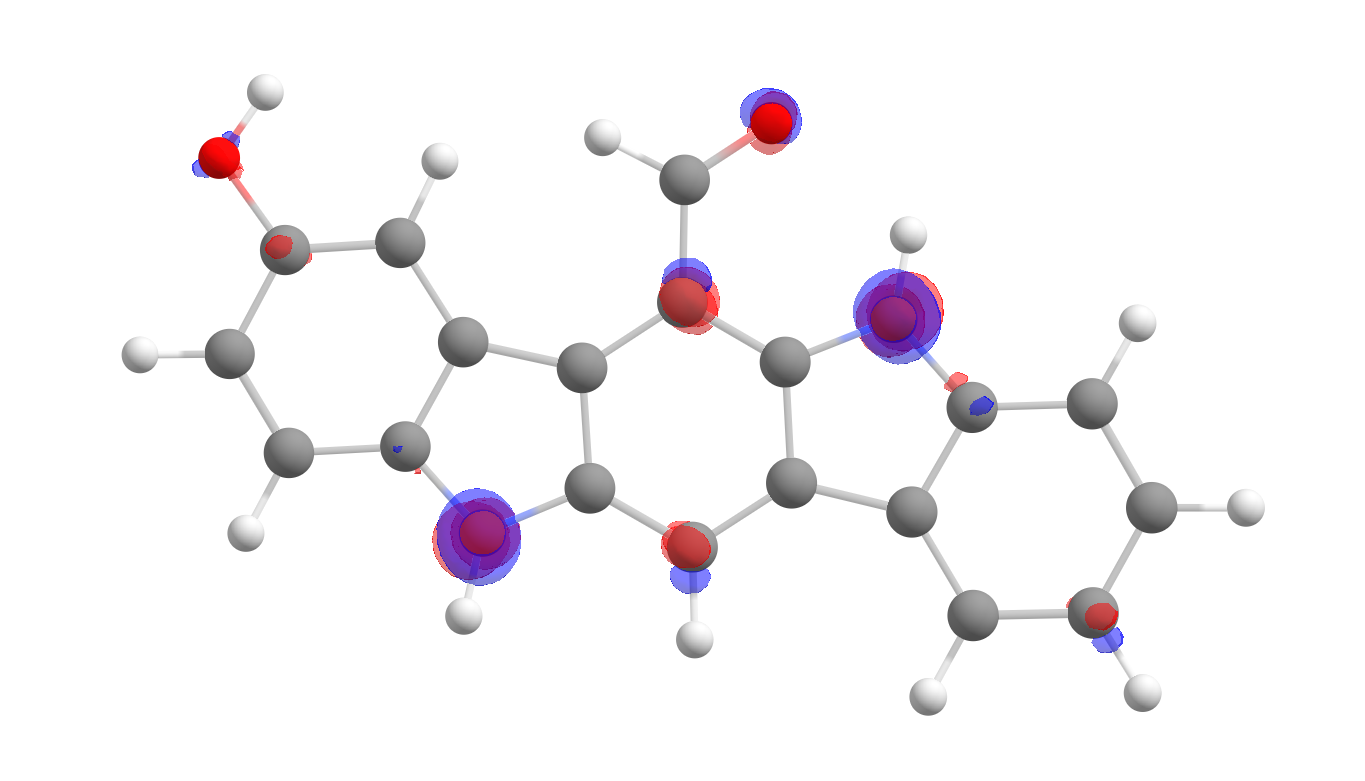** |
| $\boldsymbol{F}^{\boldsymbol{0}}$ | **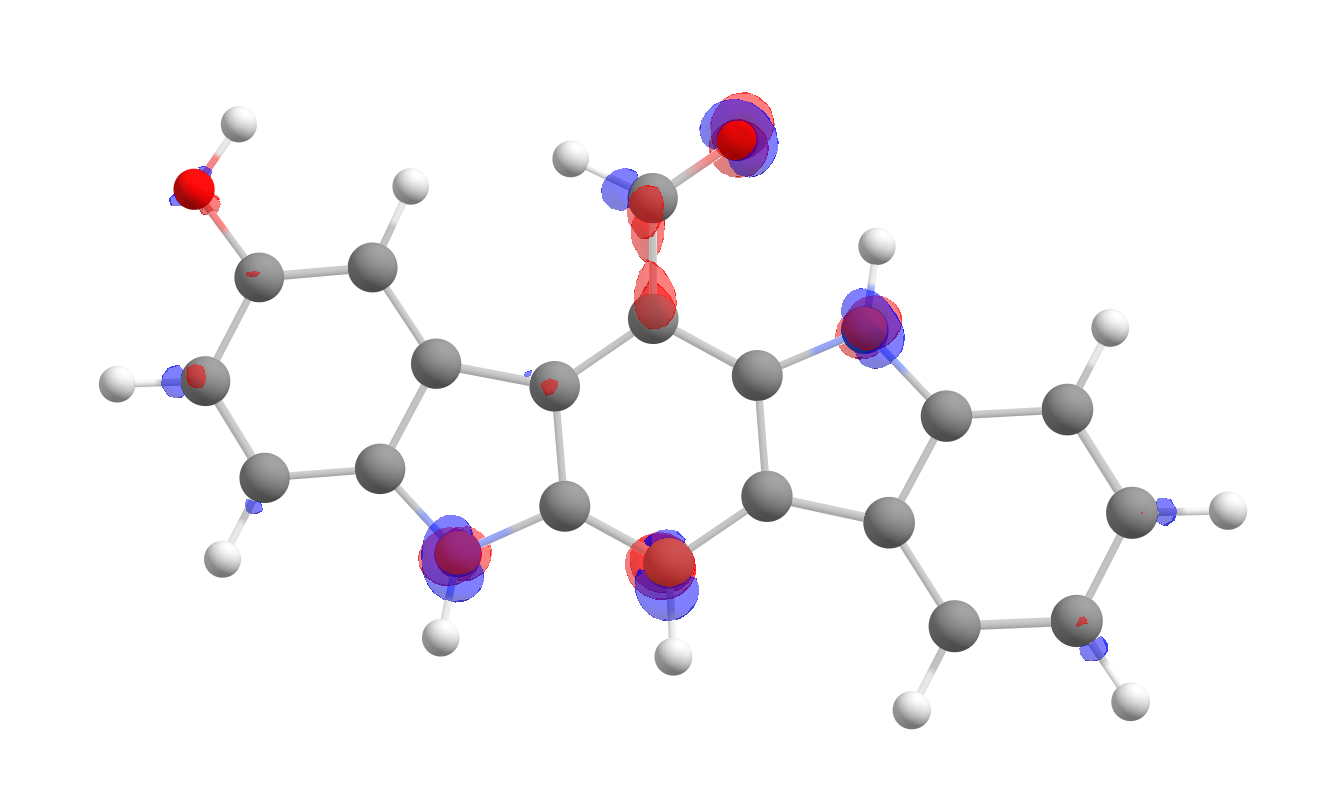** |

|  | **3** |
| --- | --- |
| $\boldsymbol{F}^{\boldsymbol{+}}$ | **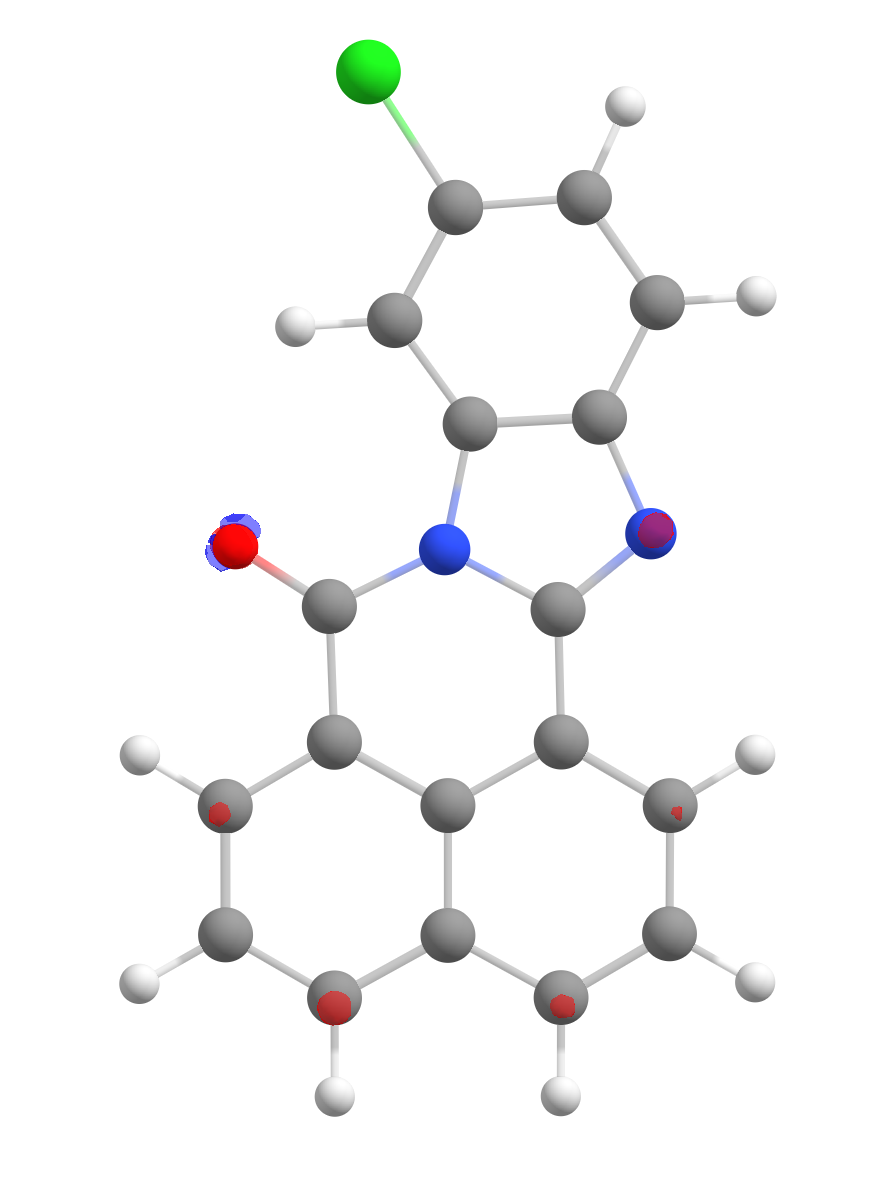** |
| $\boldsymbol{F}^{\boldsymbol{-}}$ | **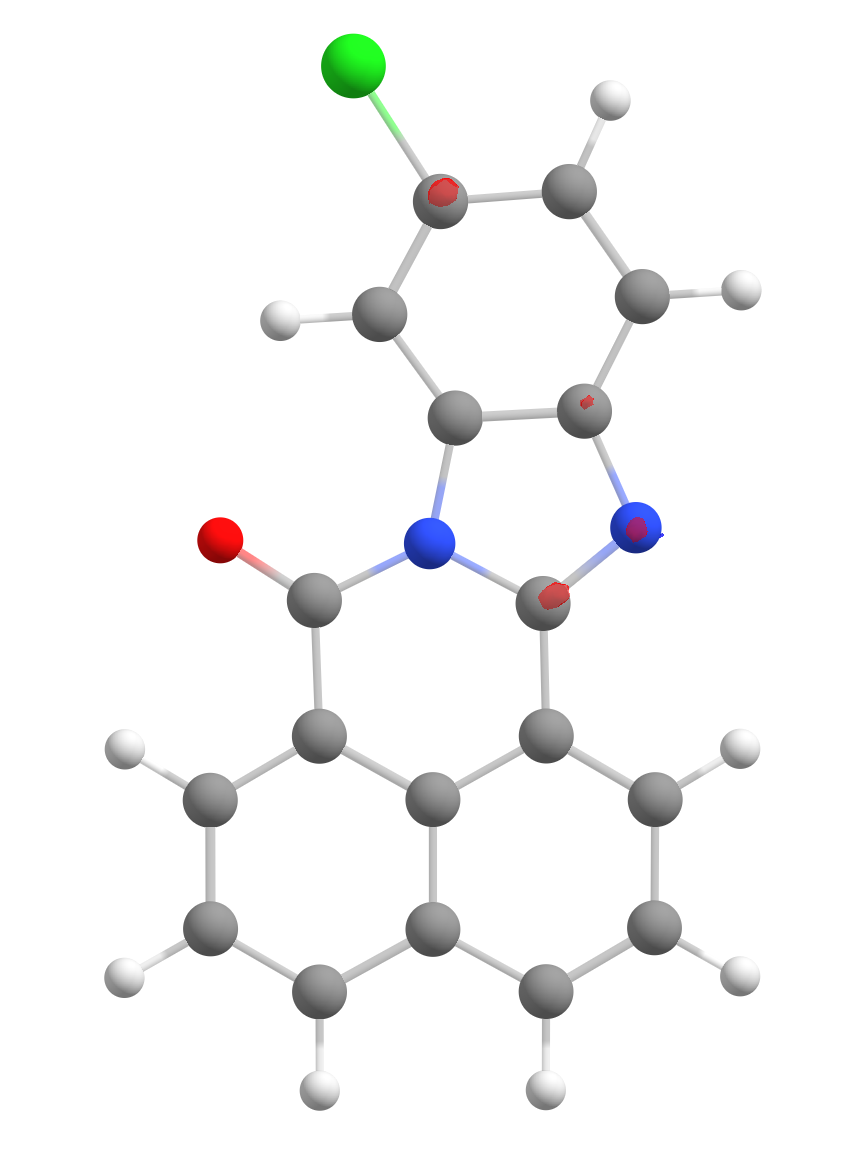** |
| $\boldsymbol{F}^{\boldsymbol{0}}$ | **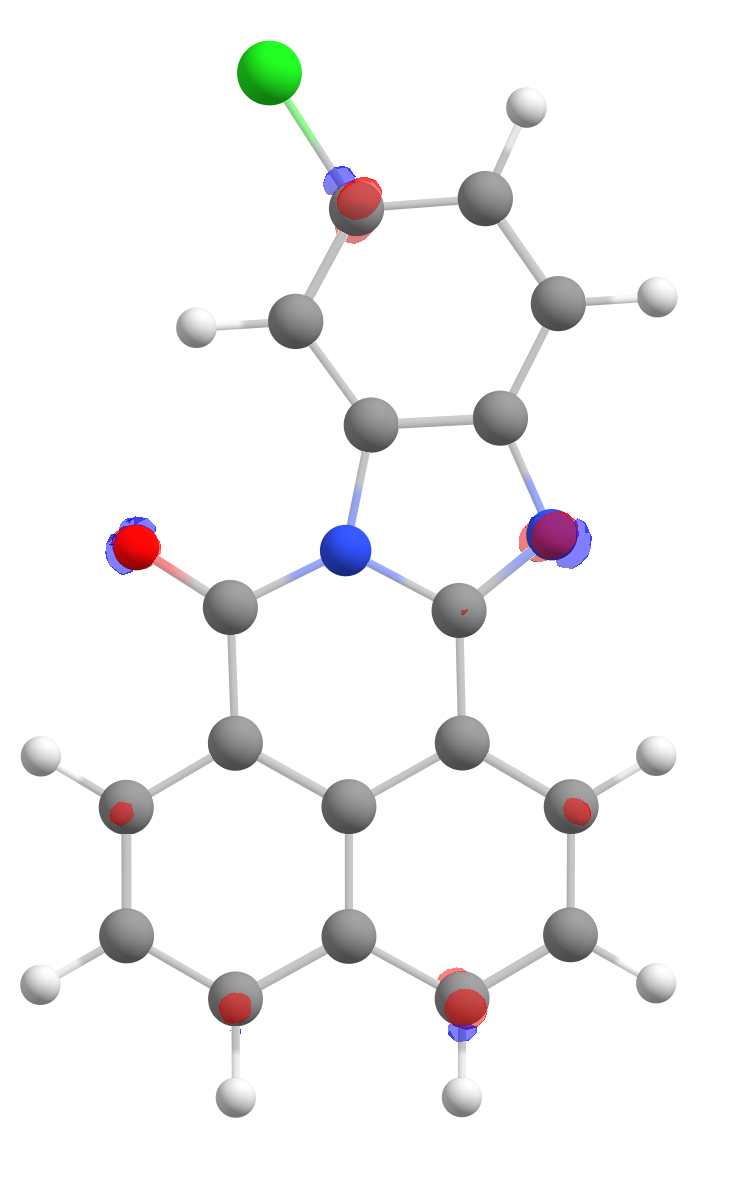** |

|  | **11b** |
| --- | --- |
| $\boldsymbol{F}^{\boldsymbol{+}}$ | **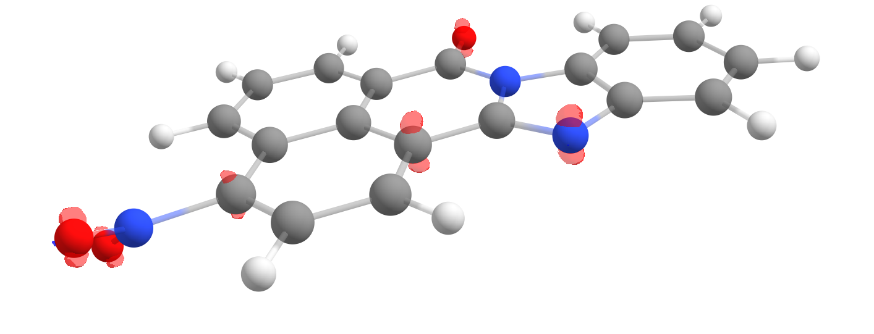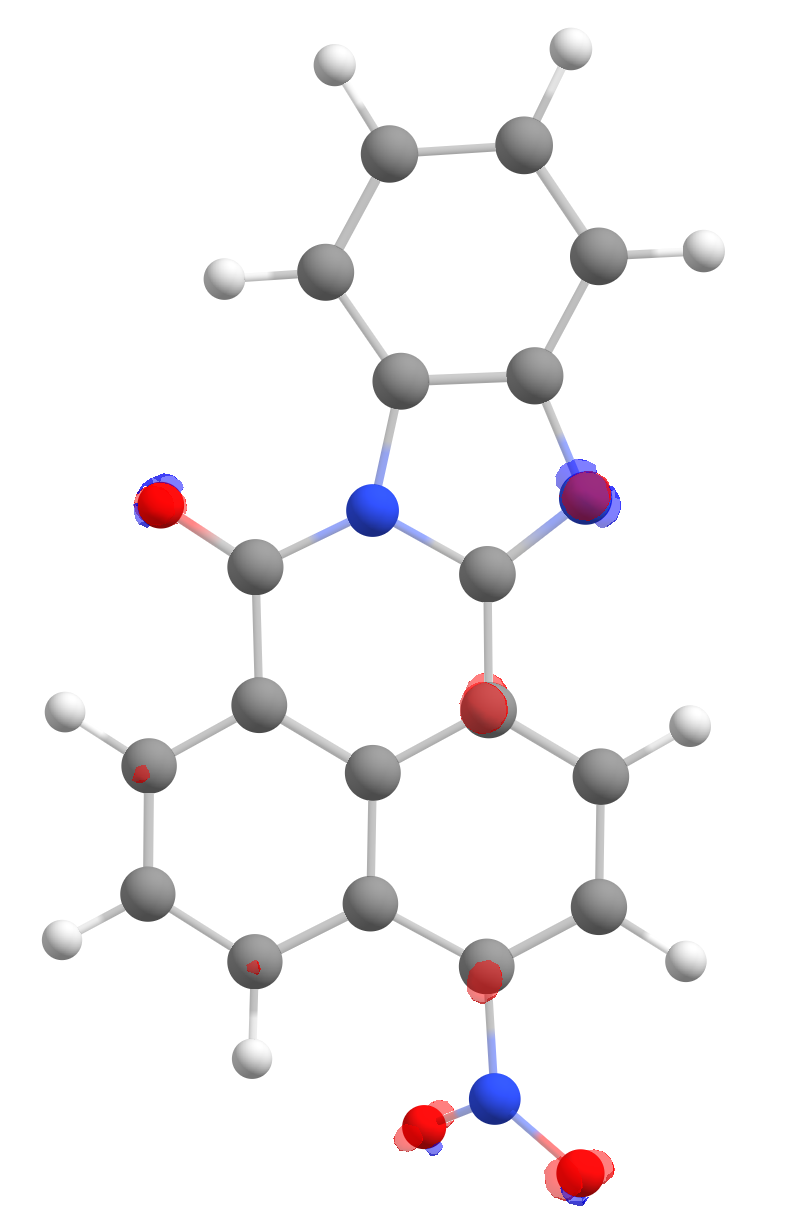** |
| $\boldsymbol{F}^{\boldsymbol{-}}$ | **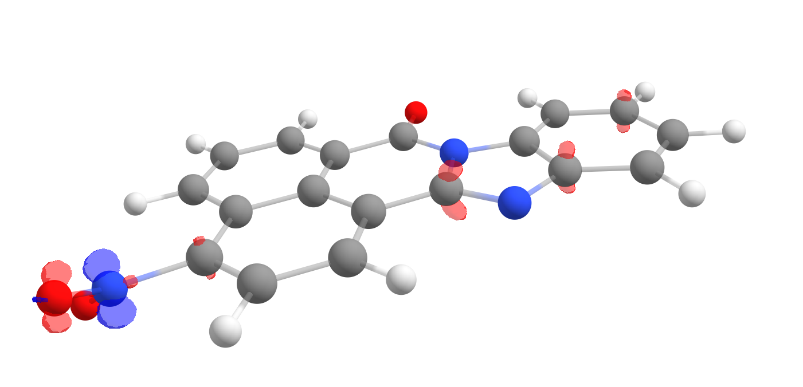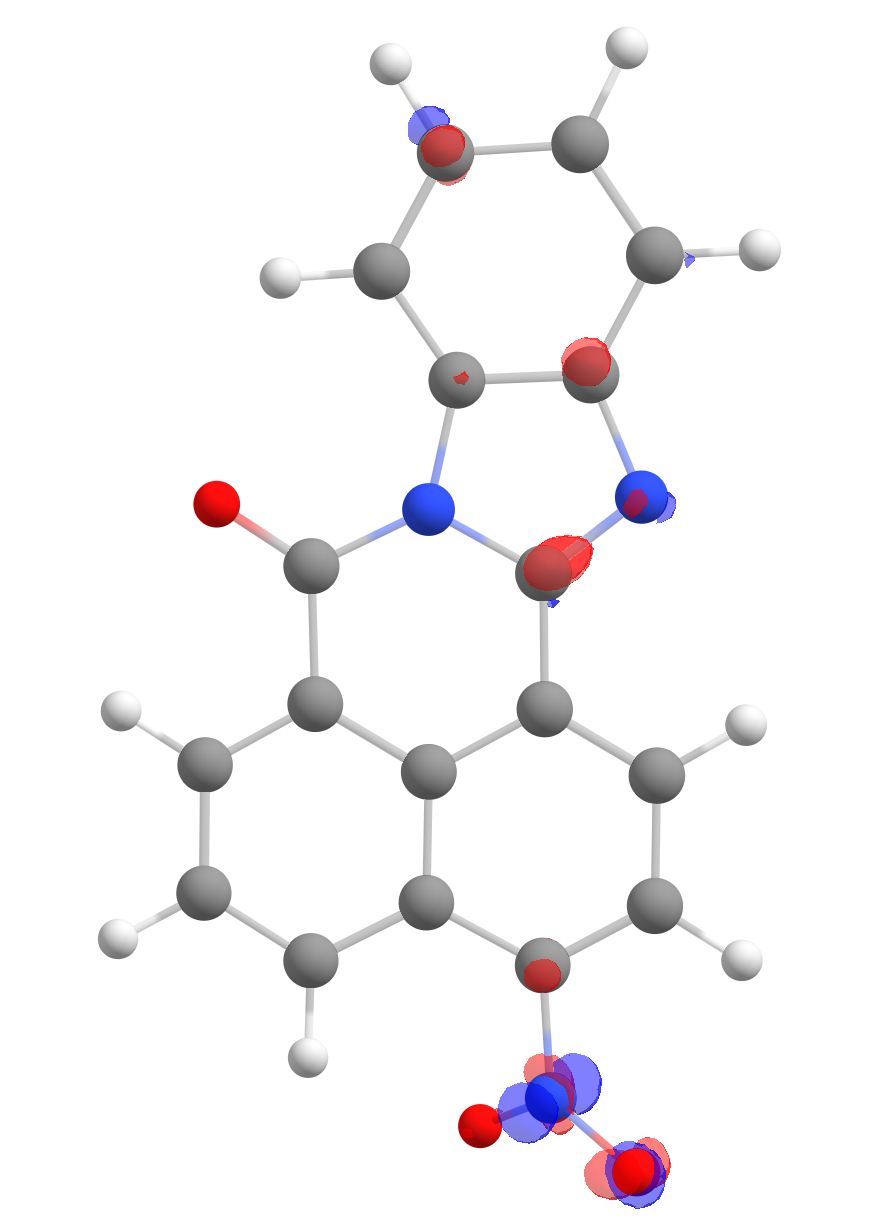** |
| $\boldsymbol{F}^{\boldsymbol{0}}$ | **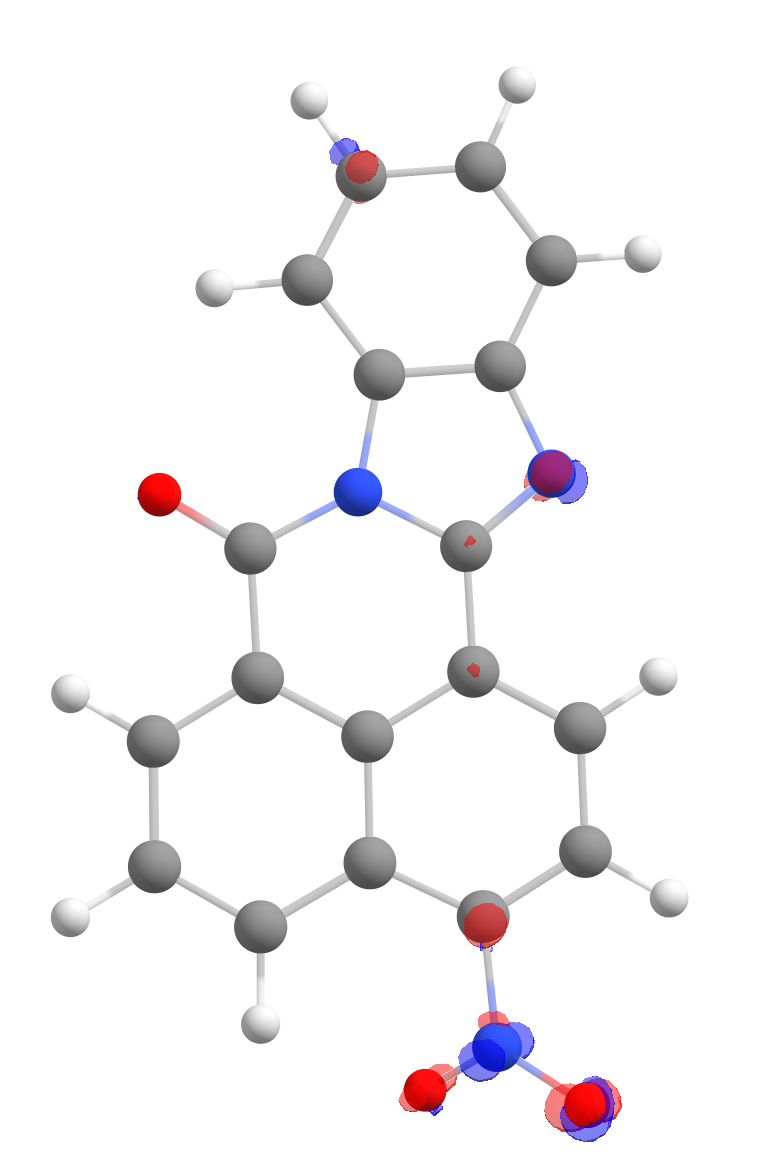** |
|  |  |

# Compound characterisation

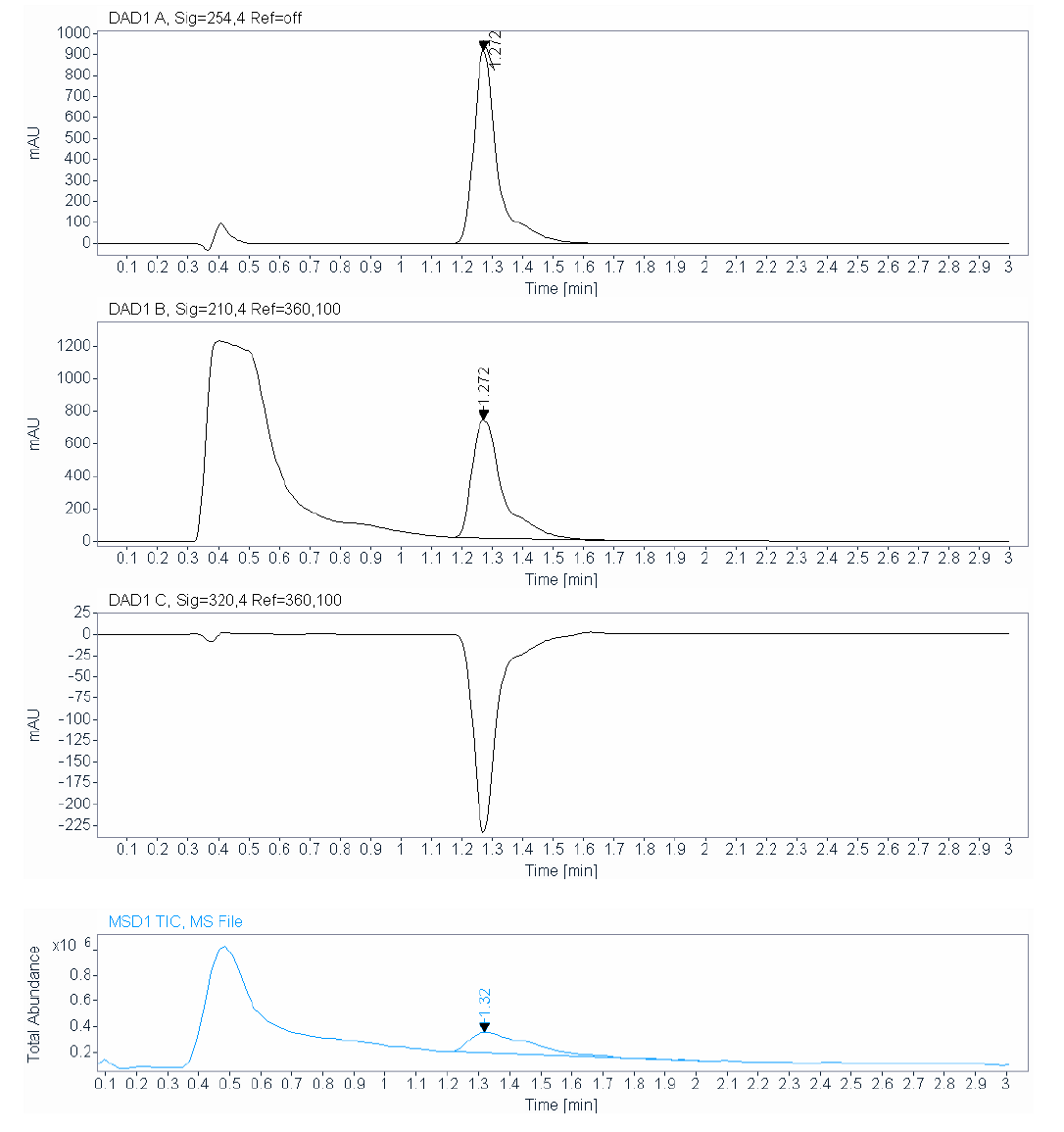


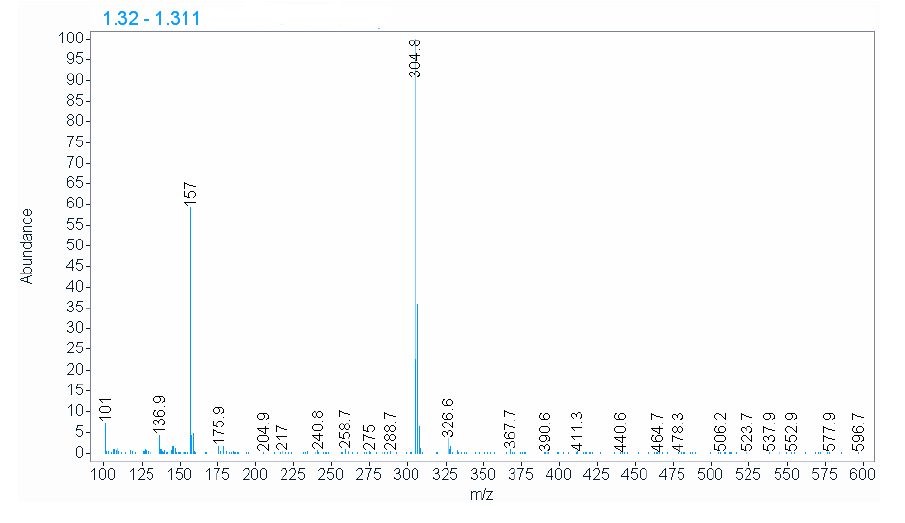

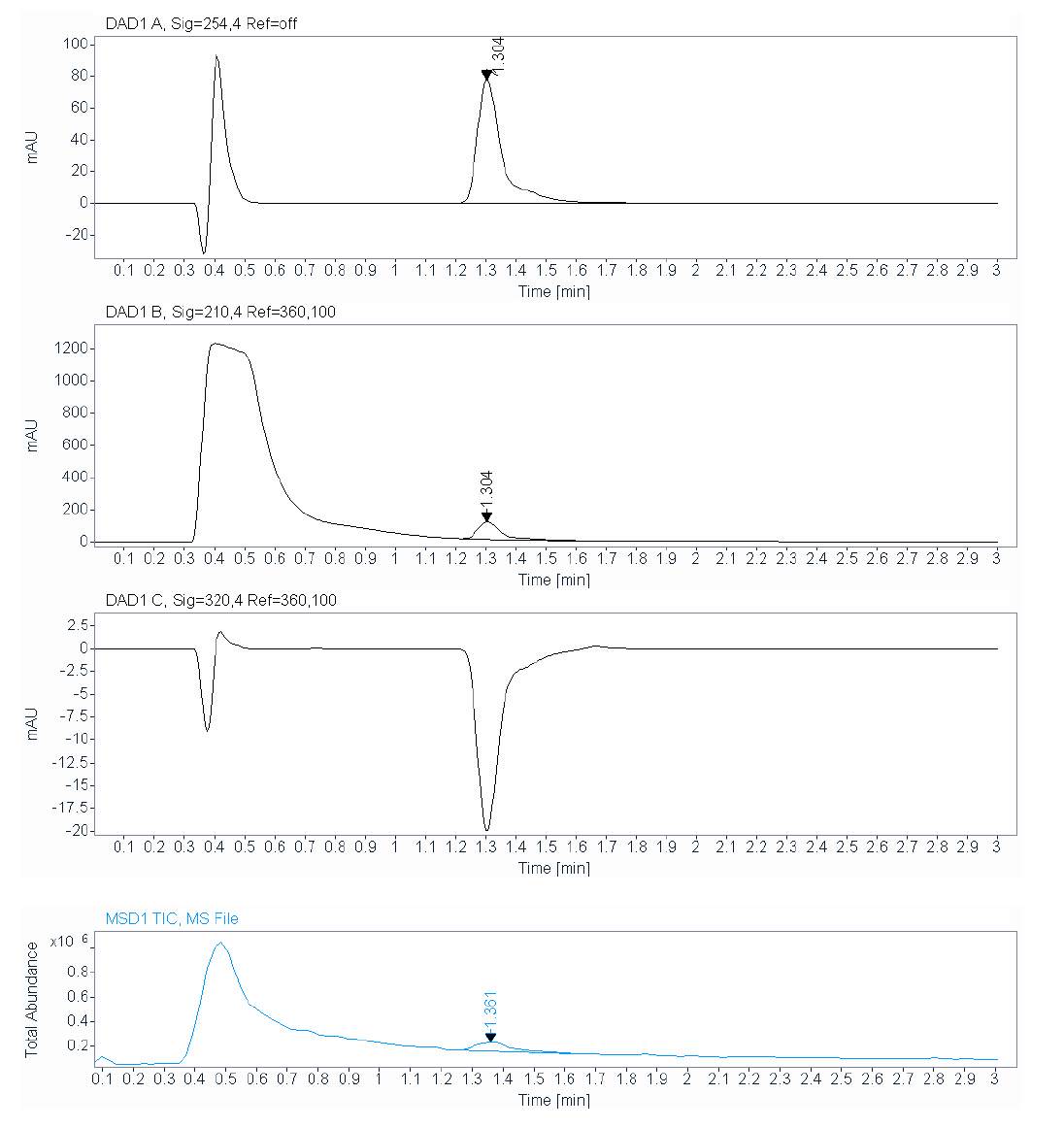


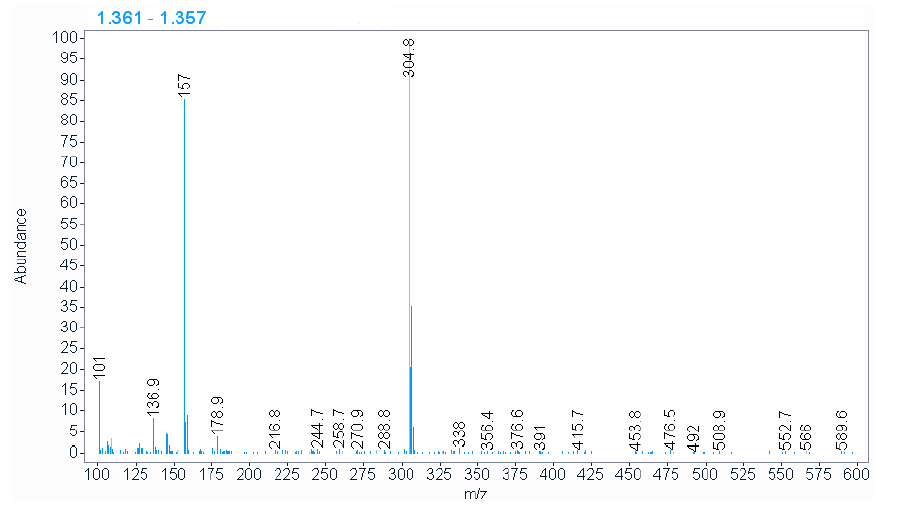

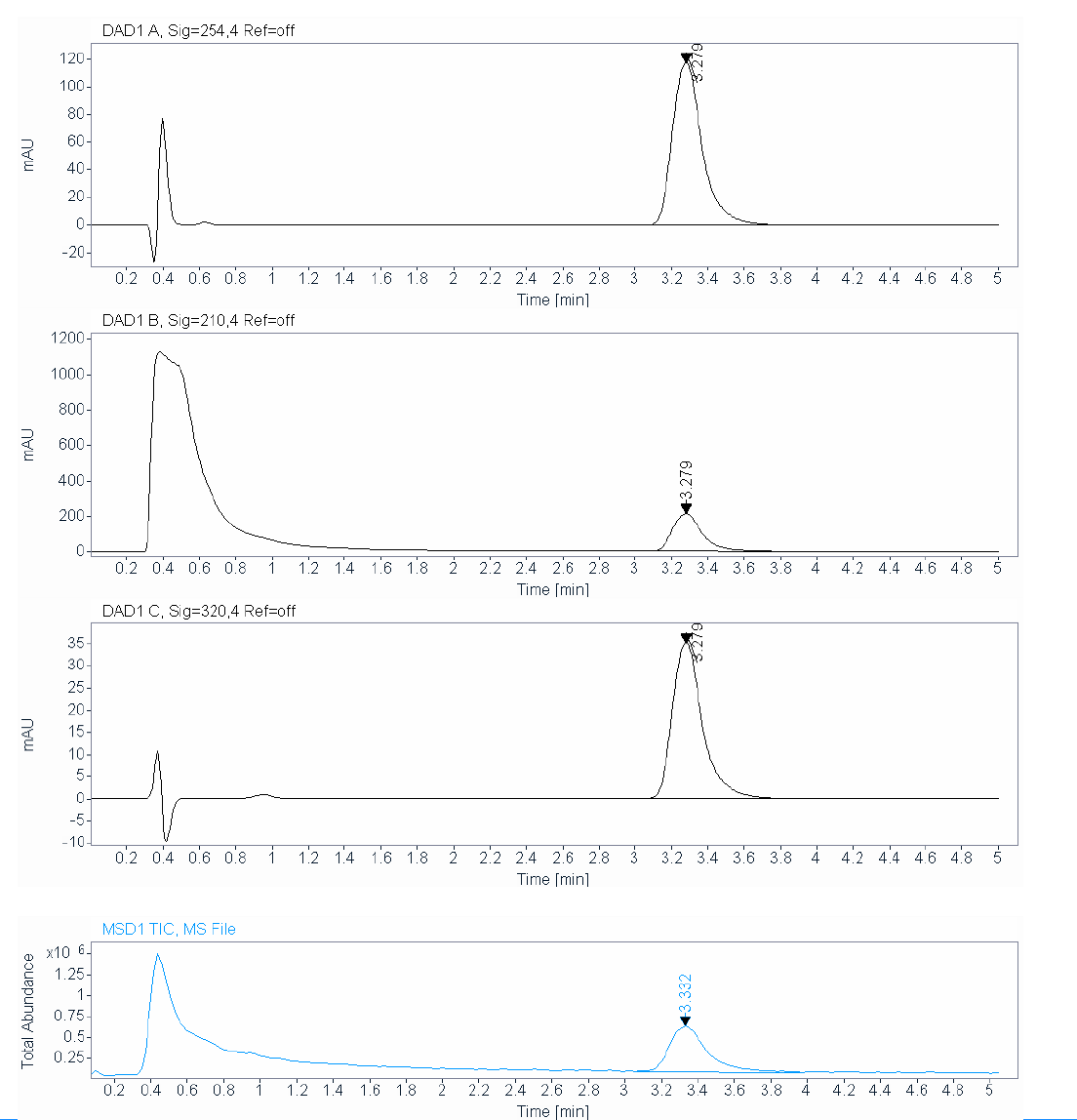


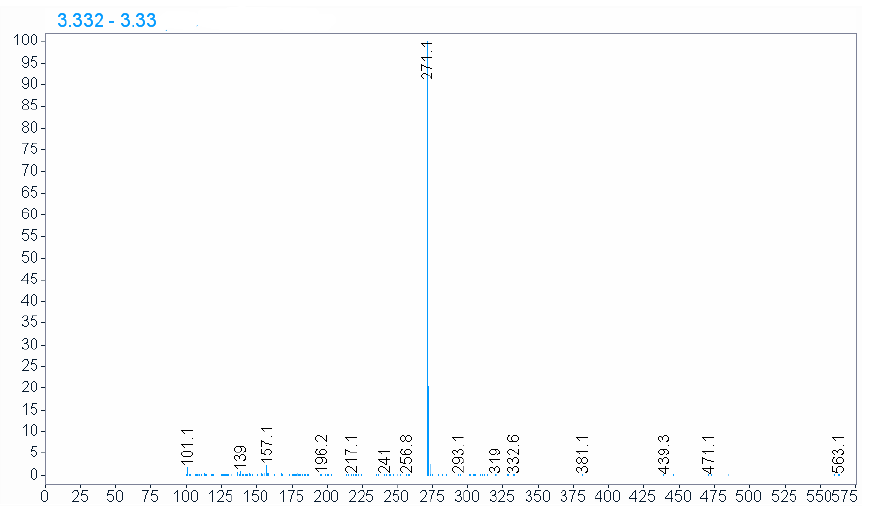

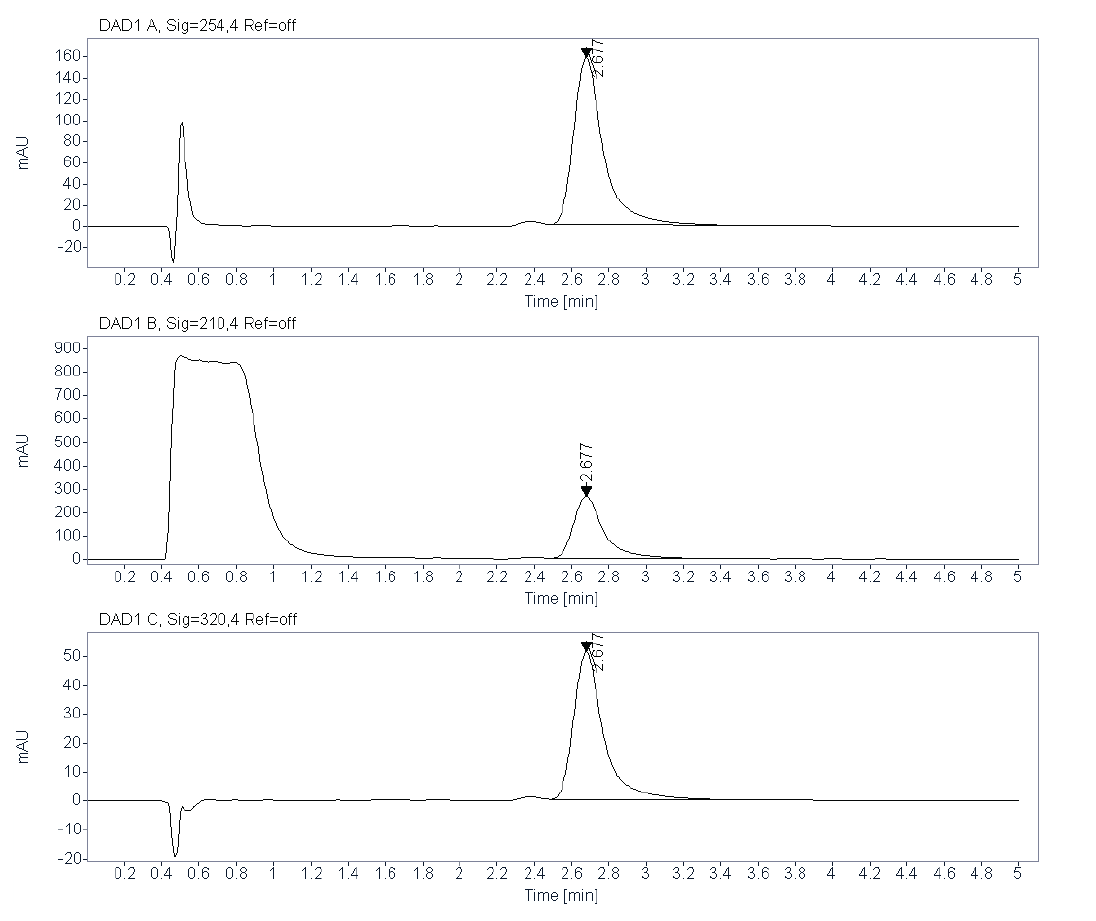


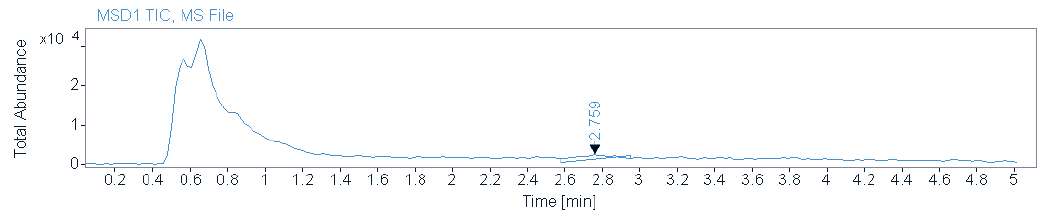


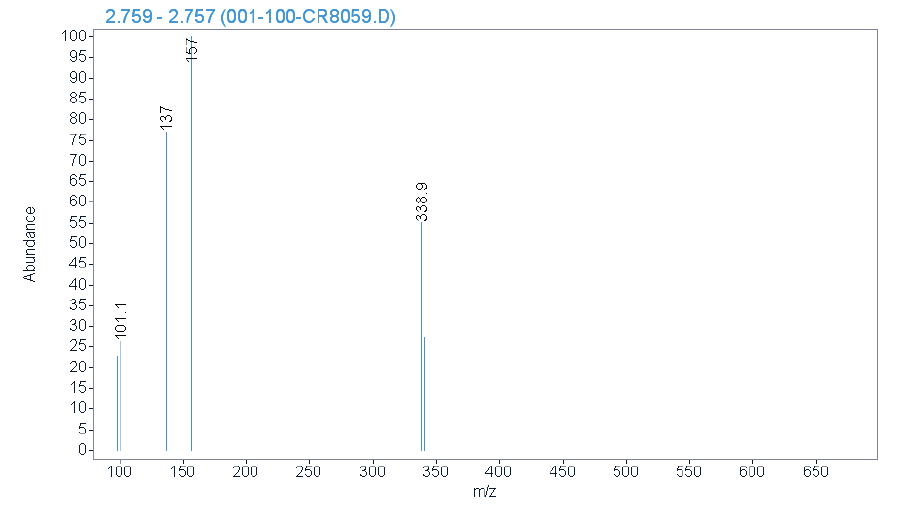

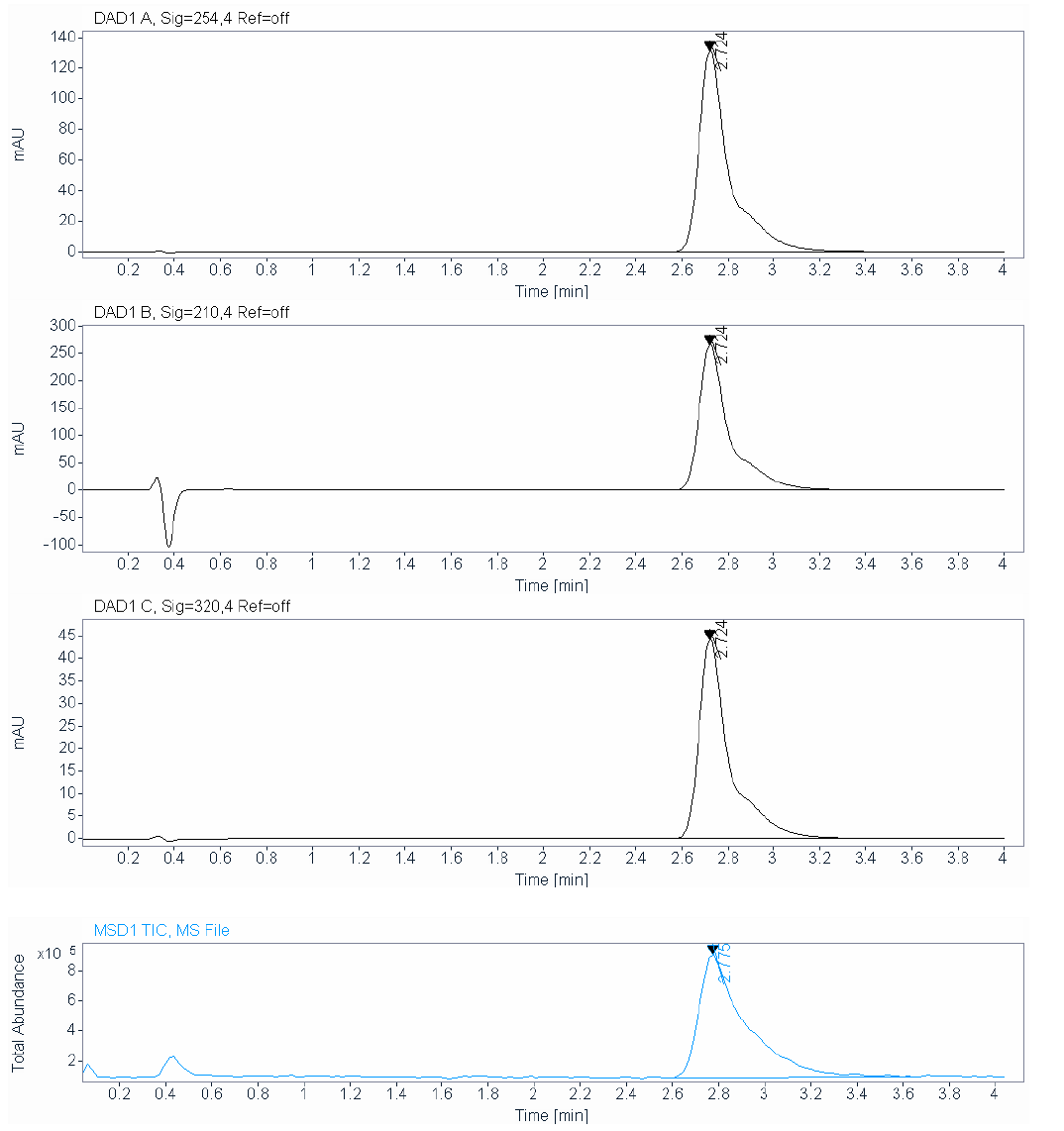


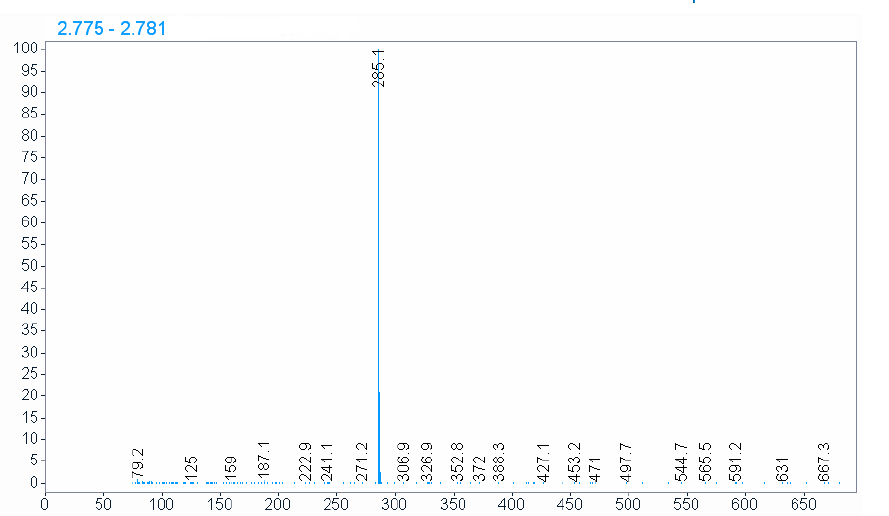

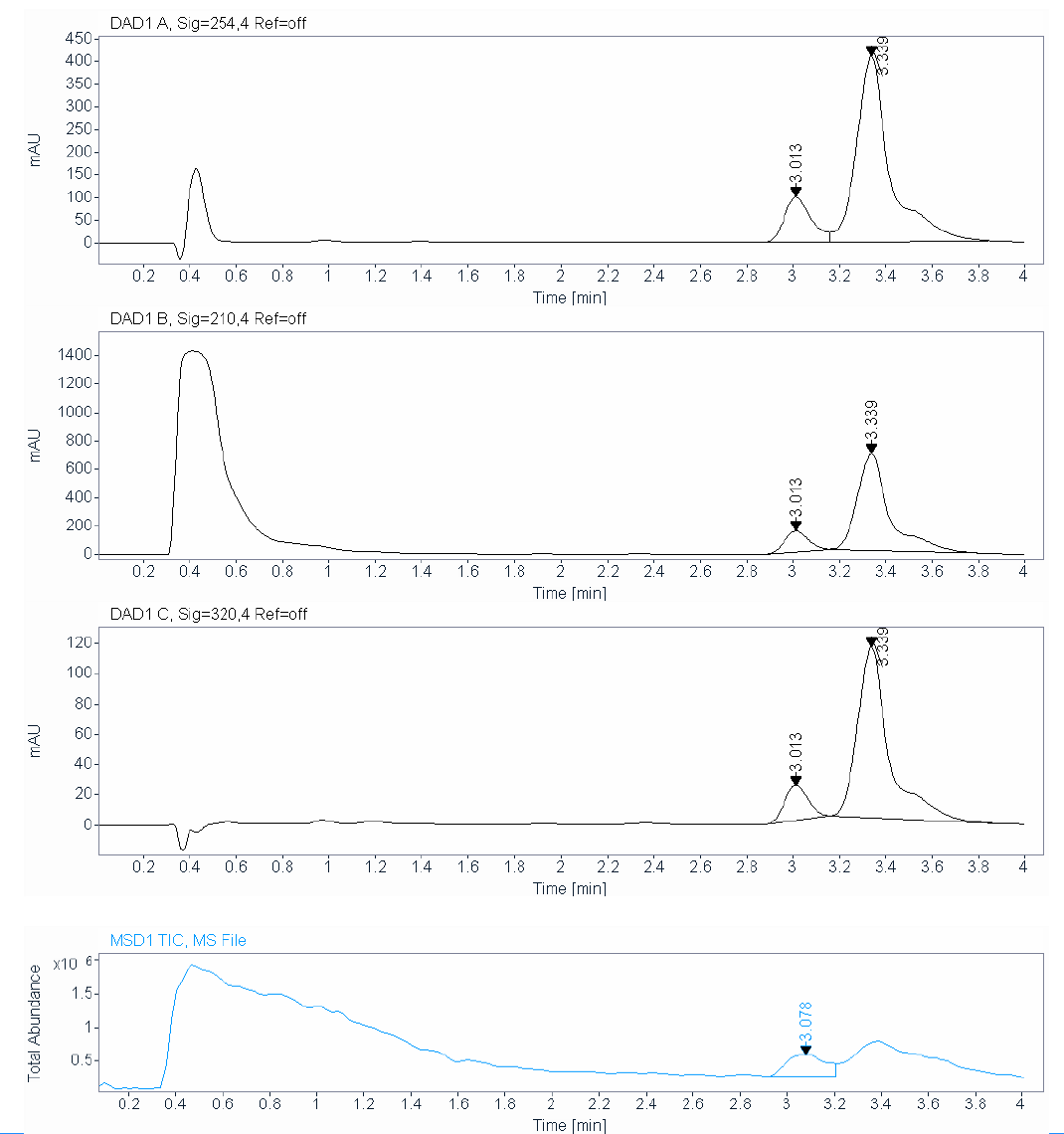


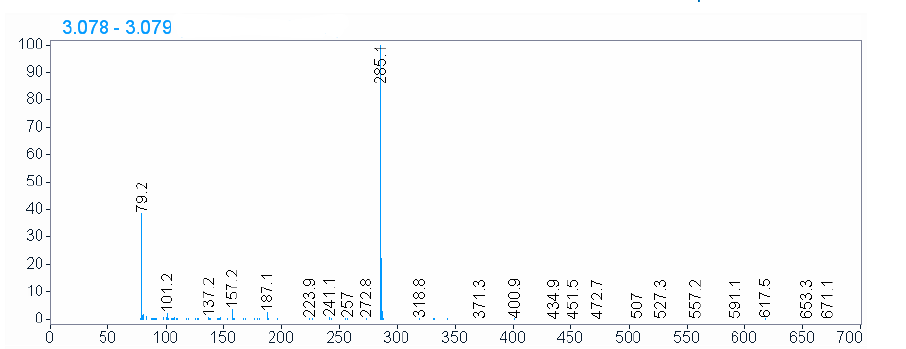

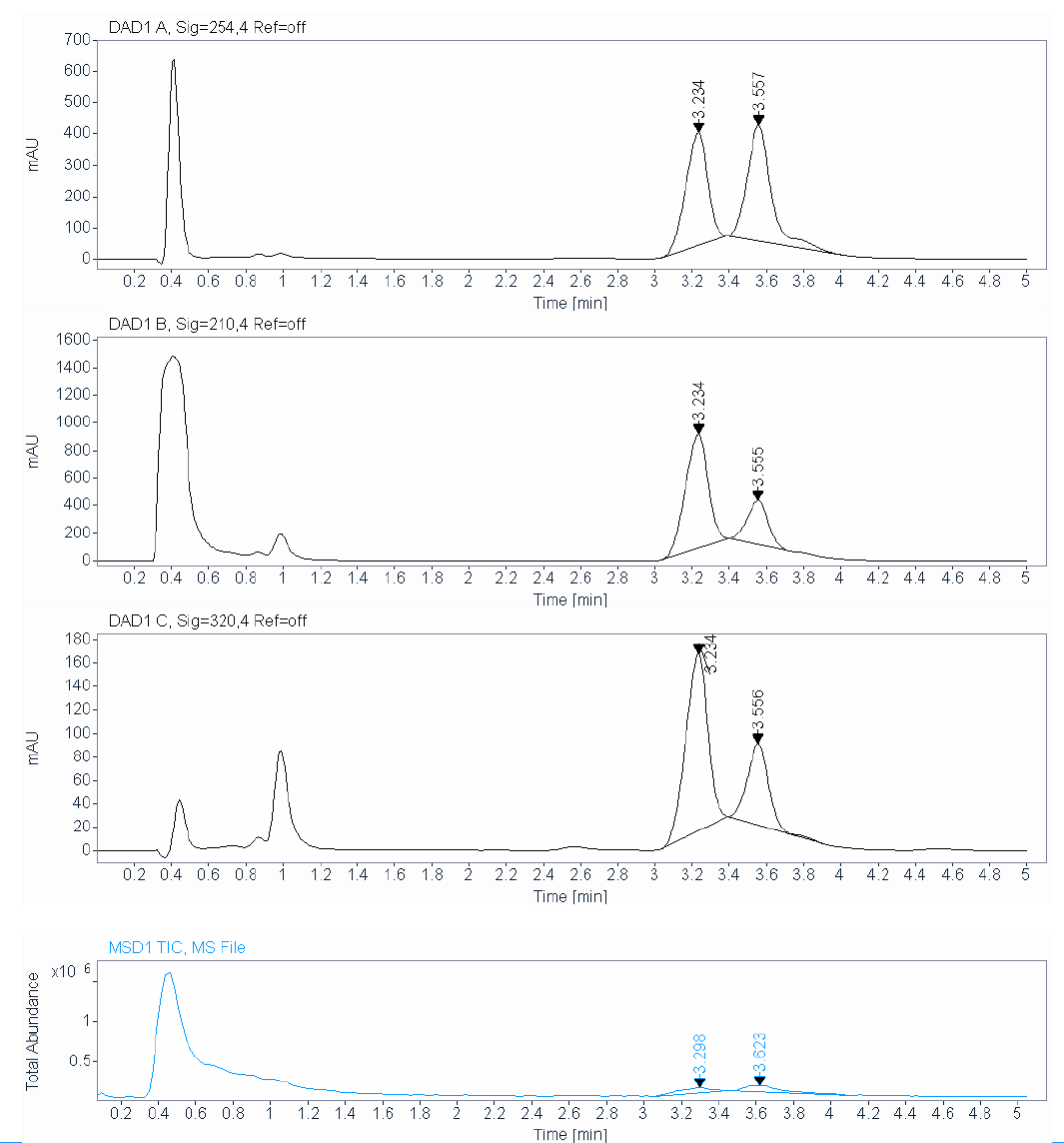


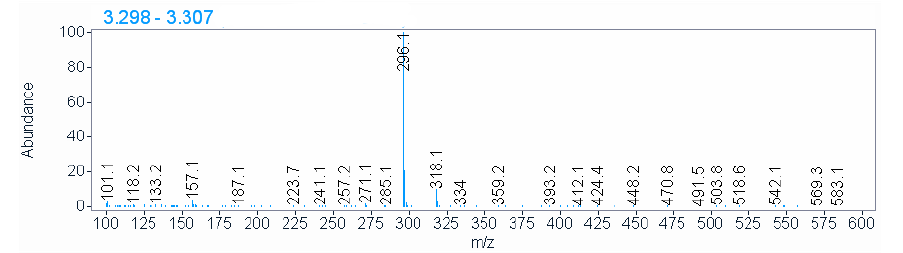

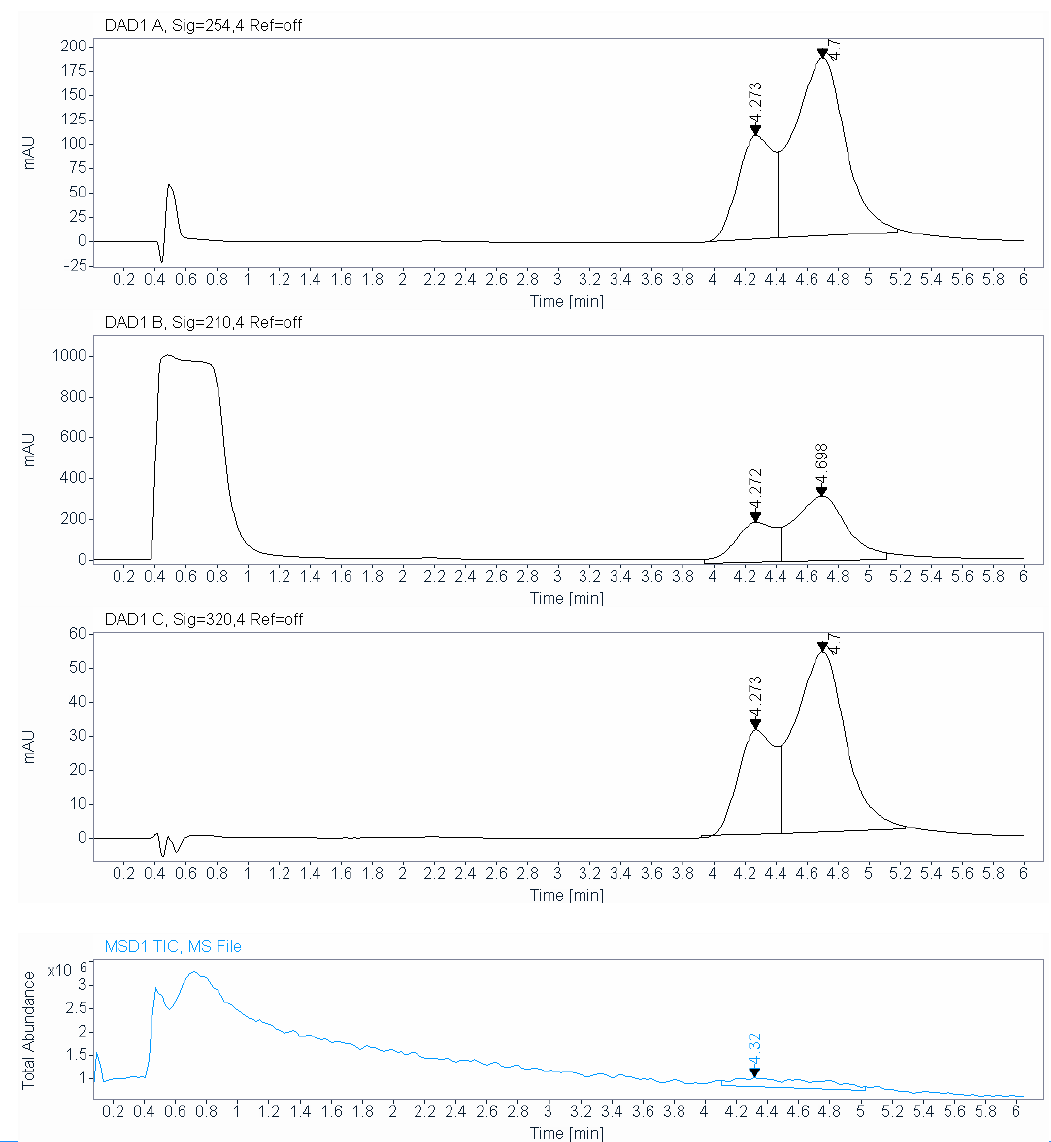


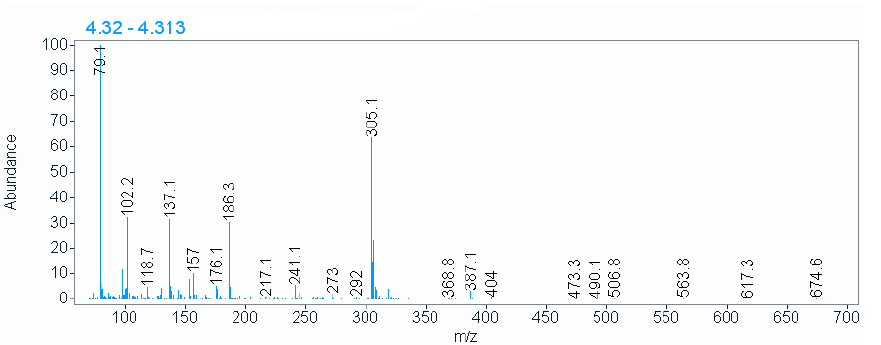

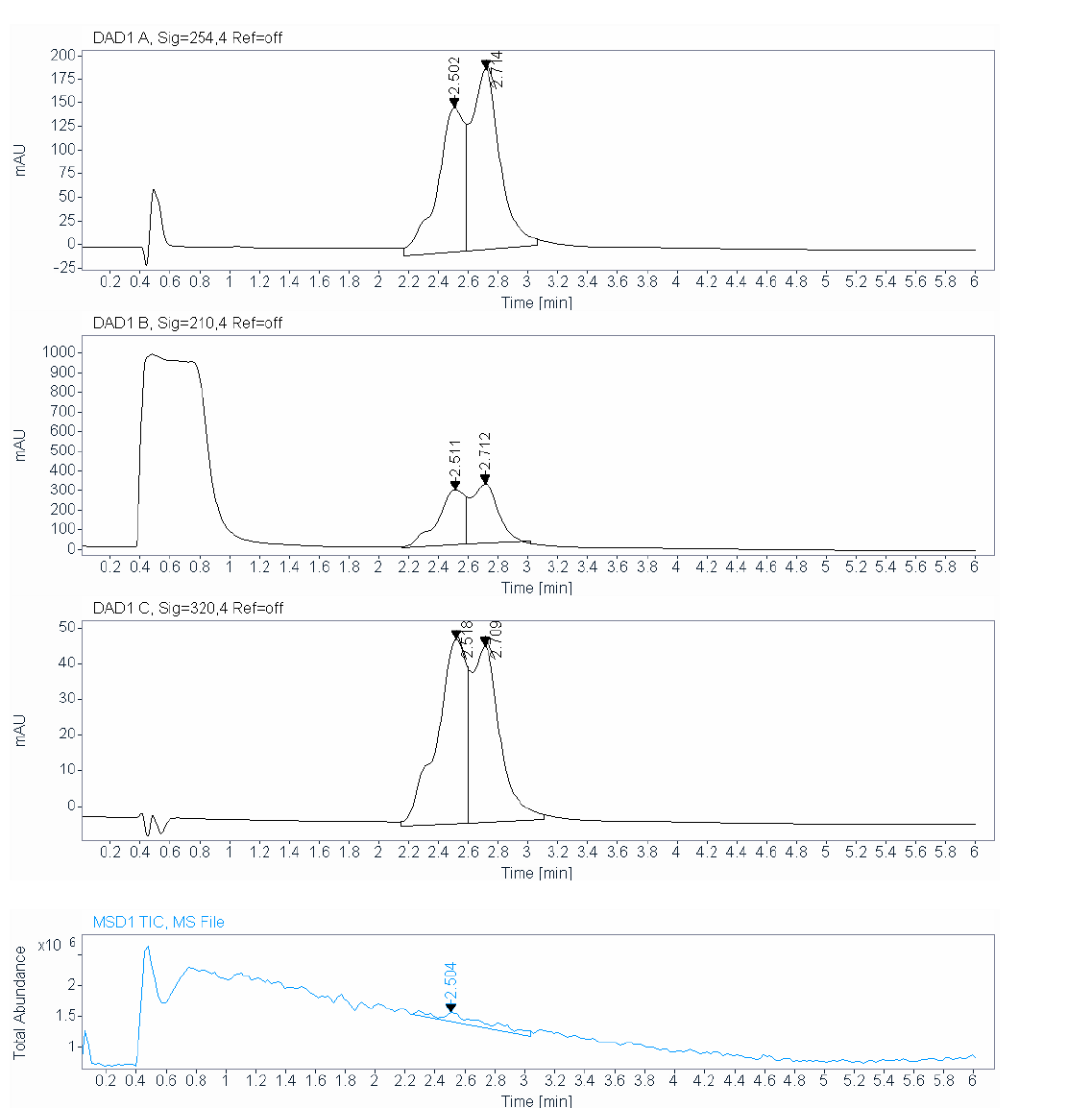


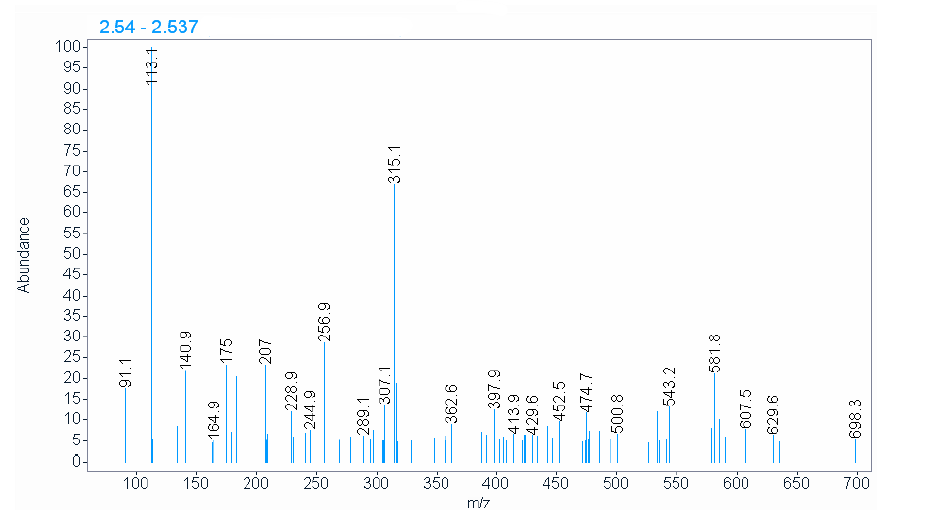

Paula, S., Baker, J. R., Zhu, X., and McCluskey, A. (2019). Molecular Docking and Molecular Dynamics. doi: 10.5772/intechopen.84818
